# Bismuth subsalicylate, a low-toxicity catalyst for the ring-opening polymerization (ROP) of l-lactide (l-LA) with aliphatic diol initiators: synthesis, characterization, and mechanism of initiation†

**DOI:** 10.1039/d0ra05413e

**Published:** 2020-08-20

**Authors:** María Guadalupe Ortiz-Aldaco, José E. Báez, J. Oscar C. Jiménez-Halla

**Affiliations:** Department of Chemistry, University of Guanajuato (UG) Noria Alta S/N 36050 Guanajuato Gto. Mexico jebaez@ugto.mx

## Abstract

The ring-opening polymerization (ROP) of l-lactide (l-LA) was induced by the catalytic action of bismuth subsalicylate (BiSS) using linear aliphatic diols [HO(CH_2_)_*n*_OH, where *n* = 2, 3, 4, 5, 6, and 8] as initiators and chain transfer agents. The theoretical and experimental degree of polymerization (DP) in all samples of α,ω-hydroxy telechelic poly(l-lactide) (HOPLLAOH) had a good agreement in all samples, an effect attributed to the interaction of BiSS with HO(CH_2_)_*n*_OH inducing a transfer reaction. HOPLLAOH was synthesized and characterized by a range of analytical techniques, confirming the insertion of methylene groups from the initiator into the main chain of the polyester. The glass-transition temperature (*T*_g_) of HOPLLAOH was found to be proportional to the number of methylene groups present in the diol. Various parameters regarding the ROP of l-LA were studied, such as temperature, time of reaction, amount of catalyst, and the nature of the diols. A kinetic study of the reaction allowed the determination of the rate constants (*k*) and activation energy (*E*_a_). A mechanism of initiation is proposed based on a computational study using density functional theory (DFT), evidencing the role of the alkyl diol as an initiator, producing an alkoxide (Bi–OROH). This species then acts as a nucleophile, attacking the carbonyl group, inducing its insertion, and ultimately completing the ring-opening of l-LA.

## Abstract 2

Bismuth subsalicylate (BiSS) acted as a catalyst in the ring-opening polymerization of l-lactide (l-LA) in the presence of alkyl diols as initiators.

## Graphical abstract

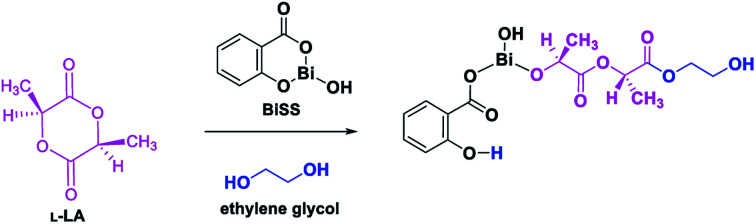

## Introduction

Aliphatic polyesters are some of the most versatile biodegradable polymers due to their moderate mechanical properties and good biodegradability and biocompatibility.^1–6^ Polylactide (PLA) and its derivatives, are frequently encountered aliphatic polyesters, having mechanical properties similar to those of polystyrene, and are characterized by a high modulus and low elongation to break.^7^ PLA is a relatively brittle plastic but possesses good strength, and as such finds utility in biomedicine, including as a controlled drug delivery carrier, tissue engineering scaffold, surgical suture, and bone fixation material.^7–10^ PLA also has stereoisomers that are interesting materials in their own right, including poly(l-lactide) (PLLA), poly(d-lactide) (PDLA), and poly(d,l-lactide) (PDLLA). PLLA has gained attention because of its excellent biocompatibility, mechanical properties,^11^ and application as a precursor for a range of polymeric materials such as scaffolds for engineered tissue, films, macrodiols, polyurethanes, and others.^12^

The conventional method for the synthesis of high molecular-weight PLLA is ring-opening polymerization (ROP).^11,13^ The three most common mechanisms used in cyclic-ester ROP are coordination–insertion, enzymatic, and anionic.^14^ Coordination–insertion ROP is initiated by an alcohol or initiator and catalyzed by metal complexes based on Lewis acidic metals such as tin, aluminum, and zinc.^15^ Studies on the ROP of cyclic esters such as l-lactide (l-LA) have been explored using different metal compounds, for example, (1) a series of binuclear zirconium salen-type complex was used as a catalyst in the ROP of l-LA and obtained a conversion >90% within 10 min, yielding high molecular weight PLLA with low dispersity, and the kinetic studies showed that the ROP of l-LA catalysed by this complex had a first-order dependency with respect to the [l-LA].^16^ (2) In another study of the ROP of l-LA, a series of Na complexes bearing ketiminate ligands used as catalyst was reported, the polymerization results revealed that all Na complexes had the highest catalytic activity at 0 °C in the presence a high concentration of BnOH with a conversion of 99%.^17^ (3) On another side, a series of *N*-arylcyano-β-diketiminate zinc complexes with cyano groups in the *ortho* position of the aromatic ring was explored as a catalyst in the polymerization of l-LA, the zinc complexes with one cyano group showed good activity in the ROP of l-LA with a conversion of 95%.^18^ Currently, tin(ii) 2-ethyl hexanoate (stannous octoate or tin octoate, SnOct_2_) is the most widely used catalyst to promote the ROP of cyclic esters despite an ongoing debate over its cytotoxicity.^19,20^

In this regard, the development of alternative, non-toxic catalytic systems is of considerable interest. The element bismuth, which is present in numerous pharmacology formulations, is considered to have very low toxicity; however, it has only been studied to a limited extent as a catalyst of ROP processes. Our interest in the use of bismuth-based catalysts is further motivated by the fact that Mexico produces the second most bismuth in the world (825 metric tonnes).^21^ A number of bismuth compounds have been reported to catalyze ROP processes, including inorganic derivatives such as BiX_3_ (X = Cl, Br, I),^22^ the homoleptic acetate,^23^ 2-ethyl hexanoate,^24^ hexanoate,^25^ triflate,^26–28^ alkoxides,^29^ the heteroleptic subsalicylate,^25,30,31^ (salen)bismuth alkoxides,^32^ and the diphenyl bismuth bromide.^33,34^ Bismuth(iii) compounds have proven to be versatile catalysts for a variety of organic transformations.^35–38^ Bismuth subsalicylate (BiSS), an antiulcer and antigastritis drug commonly marketed in oral medications (*e.g.* Pepto Bismol®),^39–41^ has recently been demonstrated to be a less toxic catalyst for the ROP of cyclic esters.^42,43^ The ROP of cyclic esters was primarily, developed by Kricheldorf *et al.*,^44^ and the same group has reported the use of bismuth(iii) complexes of carboxylic acids as useful catalysts in the ROP of ε-caprolactone (CL) and l-lactide (l-LA) to give poly(ε-caprolactones) (PCLs) and poly(l-lactides) (PLLAs).^24,45,46^ Kricheldorf has also reported that bismuth alkanoates Bi(O_2_CR)_3_ may be used in the ROP of lactones.^45^

Other bismuth species have also been demonstrated to have interesting catalytic activity, exemplified by the polymerization of l-LA at high temperatures without significant racemization initiated by bismuth(iii) acetate [Bi(OAc)_3_].^47^ These results demonstrate that Bi(OAc)_3_ is a highly efficient initiator, allowing the syntheses of di-, tri-, and tetrafunctional PLLAs. A comparison of the reactivity of Bi(OAc)_3_ with that of SnOct_2_ in the polymerization of l-LA under the same reaction conditions, showed that good conversion was achieved (>90%) with both catalysts within a rather short time.^47^

Homoleptic Bi(iii) alkoxides bearing sterically bulky alkoxide ligands (–OtBu or –OCMe_2_iPr) have also been shown to be highly active catalyst for l-LA ROP. Increasing the steric bulk of the alkoxy ligand further from –O*t*Bu to –OCMe_2_iPr decreases the coordination ability of the monomer and lowers the activity of the catalyst. These Bi(iii) alkoxides are considerably more active catalysts for l-LA ROP than the commonly-used SnOct_2_/butanol initiator combination. The simple formulation, low toxicity, and high activity of Bi(iii) alkoxides make them very attractive catalyst candidates.^48^

**Scheme 1:**
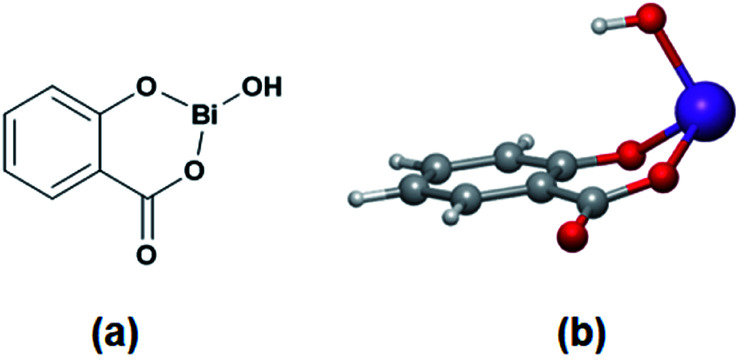
Structure of the bismuth subsalicylate (BiSS) in 2D (a) and 3D (b).

BiSS (Scheme 1) has recently been demonstrated to be a less toxic catalyst for the ROP of cyclic esters,^46–53^ and as a catalyst/initiator for homo- and copolymerizations of lactones and lactides.^53–55^ An exciting result was the discovery that BiSS favored the presence of less blocky sequences than copolyesters prepared with SnOct_2_ under identical conditions.^55^ However, neither the mechanism of the reaction nor the ROP of lactones using BiSS as a catalyst has been reported. The significant difference in toxicity between BiSS and SnOct_2_ (Table 1),^56^ suggest the possibility of replacing tin-based catalyst with BiSS in the ROP of lactones. In this work, we reported the use of BiSS as a catalyst of the ROP of l-LA in the presence of alkyl diols (HOROH) as initiators, aspects as synthesis, characterization, and mechanism of initiation are shown.

**Table 1:** Comparison of the toxicity according to the globally harmonized system (GHS) for hazard communication^56^

Compound	CAS registry number	GHS
BiSS	14882-18-9	Non-dangerous substance
Sn(Oct)_2_	301-10-0	Health hazard, corrosive, and exclamation mark (acute toxicity: oral, dermal, inhalation)

## Experimental

### Materials

All reagents, l-lactide (l-LA, purity 99%), bismuth subsalicylate (BiSS, purity 99.9%), ethylene glycol (purity 99.8%), 1,3-propanediol (purity 98%), 1,4-butanediol (purity 99%), 1,5-pentanediol (purity 96%), 1,6-hexanediol (purity 99%) and 1,8-octanediol (purity 98%) were purchased from Sigma-Aldrich and used as received.

### Synthesis of α,ω-hydroxy telechelic poly(l-lactide) (HO–PLLA_8_–OH)

The ring-opening polymerization (ROP) of l-LA to synthesize poly(l-lactide) macrodiols (HOPLLAOH) using linear aliphatic diols [HO–[CH_2_]_*m*_–OH, *m* = 2, 3, 4, 5, 6 and 8] as initiators and BiSS as catalyst, was carried out in a dried 20 mL vial. l-Lactide (l-LA, 20 mmol, 2.88 g), BiSS (0.03 mmol, 10.86 mg), and, 1,8-octanediol (2 mmol, 292 mg) were added to the flask and heated to reflux by stirring in an oil bath at 140 °C for 80 min (molar ratio l-LA/BSS = 667, l-LA/1,8-octanediol = 10). The resulting PLLA was precipitated from chloroform/methanol, recovered by filtration, and dried under vacuum. The other PLLA oligo-esters were prepared and isolated using the same methodology as described for HOPLLA_8_OH. Number-average molecular weight (*M*_n_) and conversion were monitored by ^1^H NMR spectroscopy (Fig. 3): *M*_n_ (theo) = 1590, *M*_n_ (NMR) = 1500, *M*_n_ (SEC) = 2500, *Ð*_M_ = 1.09, DP_NMR_ = 18.8 (conv. 99%). A simple and well-established method to determine the presence of hydroxyl end groups in a polymer was used, namely derivatization with trifluoroacetic anhydride (TFAA) to generate a trifluoroacetate derivative (Fig. S6†), and in some cases, this prevents the overlapping of NMR spectroscopic signals. ^1^H NMR (500 MHz, CDCl_3_, ppm, Fig. 3): *δ* 5.16 (q, 1H, [–CH(CH_3_)–O–], PLLA), 5.05 (q, 1H, [O=CH(CH_3_)–O], l-LA), 4.35 (m, 1H, [–CH(CH_3_)–OH], PLLA), 4.12 (m, 2H, [–CH_2_–O–(C=O)–], Oct), 1.68 (d, 3H, [–CH(CH_3_)–OCOCH_2_–], PLLA), 1.58 (m, 3H, [–CH(CH_3_)–O–], PLLA), 1.49 (d, 3H, [–CH(CH_3_)–OH], PLLA), 1.31 (s, 2H, [–CH_2_–], Oct). ^13^C NMR (125 MHz, CDCl_3_, ppm, Fig. S22†): HO–^*h*^CH(^*i*^CH_3_)–^*j*^CO–[O–^*k*^CH(^*l*^CH_3_)–^*m*^CO]_*n*_–O–^*n*^CH(^*o*^CH_3_)–^*p*^CO–O–^*q*^CH_2_–(^*r*^CH_2_)_6_–^*q*^CH_2_–O–^*p*^CO–^*n*^CH(^*o*^CH_3_)–O–[^*m*^CO–^*k*^CH(^*l*^CH_3_)–O]_*n*_–^*j*^CO–^*h*^CH(^*i*^CH_3_)–OH: *δ* 175.27 (j), 169.73 (m), 167.50 (p), 72.59 (n), 69.22 (k), 66.84 (h), 65.69 (q), 28.53 (r), 20.62 (i), 16.76 (l), 15.93 (o). IR (cm^−1^) (Fig. 4): 3510 (O–H, *ν*), 2995 (CH_3_, *ν*_as_), 2942 (CH_2_ and CH, *ν*_as_), 2861 (CH_3_, *ν*_s_), 1748 (C=O, *ν*), 1452 (CH_3_, *δ*_as_), 1178 (–C–(C=O)–O–, *ν*_as_), 1085 (–C–O–C–, *ν*), 753 (CH, *γ*). DSC data (Fig. 5a): *T*_g_ = 7 °C.

### Characterization

FT-IR spectra were recorded on a PerkinElmer Spectrum 100 FT-IR spectrophotometer with attenuated total reflectance spectroscopy (ATR) accessory. ^1^H and ^13^C NMR spectra were recorded at room temperature on a 500 MHz Bruker Avance III HD instrument, using CDCl_3_ as a solvent. Chemical shifts are reported as *δ* in parts per million (ppm) and referenced to the chemical shift of the residual solvent (^13^C at *δ* 77.16, and ^1^H at *δ* 7.26, for CDCl_3_). SEC was used to determine the number-average molecular weight (*M*_n_) of the HOPLLAOH using an Agilent Technologies SEC 1260 Infinity apparatus. THF was the mobile phase at a flow rate of 10 mL min^−1^ at 37 °C using a PL gel 5 μm MIXED-D column and coupled to a 1270 RID refractive index detector.

The results are reported relative to polystyrene standards. Thermograms were performed in a Differential Scanning Calorimetry (DSC) Q200 instrument. Three scans were obtained with two heating scans (25 to 170 °C and −30 to 170 °C) and one cooling scan (170 to −30 °C) between them. The rate of heating/cooling was 10 °C min^−1^ and was performed under a nitrogen purge. The glass transition temperature (*T*_g_) is given as an inflection point, and the data presented are taken from the second heating scan.

### Computational details

All calculations were performed using density functional theory (DFT) with the Gaussian 09 program.^57^ Geometries were optimized with the PBE0 hybrid functional,^58,59^ which combines the pure non-local functional PBE^60,61^ with 25% of exact HF exchange, and the Pople's split-valence basis set of double-*ζ* quality with one polarization function (for heavy atoms), 6-31G(d), for all the atoms, and the much heavier Bi atom was treated with the LANL2DZ relativistic pseudopotential.^62^ Harmonic frequency calculations were carried out to verify the nature of the stationary points found (structures which are minima in energy have zero negative – *i.e.* imaginary – eigenvalues of the Hessian, while transition states, structures which are maxima in energy, possess one and only one negative eigenvalue, which in turn defines the reaction coordinate for each species) and to obtain the energy corrections to the electronic energy (zero-point energy, thermal and entropy corrections measured at 413.15 K and 1 atm).

Finally, we have also added dispersion corrections (Grimme-D3 method)^63^ to the electronic Hamiltonians by performing single-point calculations over the optimized geometries at the D3-PBE0/[6-31G(d), LANL2DZ] level of theory.

## Results and discussion

### Synthesis and characterization

**Scheme 2:**
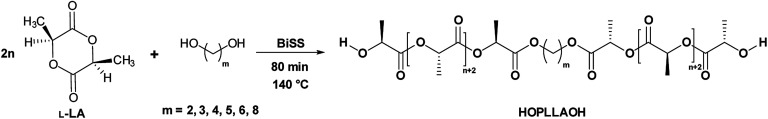
Synthesis of α,ω-hydroxy telechelic poly(l-lactide) (HOPLLAOH) by ROP of l-LA catalyzed by BiSS in the presence of an alkyl diol initiator.

In the ring-opening polymerization (ROP) of lactones, such as l-lactide (l-LA), bulk polymerization is generally preferred route as the solvent can be avoided, reducing solvent waste, complementary, in this work, only oligomers were synthesized. So, the viscosity attributed to the bulk polymerization during the polymerization reaction is not high enough due to the low *M*_n_ (oligomers) and favoring the diffusion of the monomer. Additionally, alcohols (ROH) or diols (HOROH) haves been used as initiators due to their ability to induce a transfer reaction in the presence of a catalyst, producing a new alkoxide *in situ* and controlling the degree of polymerization (DP). In our case, bismuth subsalicylate (BiSS) (Scheme 1) was tested as a catalyst for the ROP of l-LA in bulk conditions and in the presence of 1,5-pentanediol [HO(CH_2_)_5_OH] as an initiator (Scheme 2). Previously, Kricheldorf *et al.* synthesized PLLA using BiSS as catalyst in the ROP of l-LA in absent of alcohols or diols obtaining high *M*_n_.^64^

**Fig. 1:**
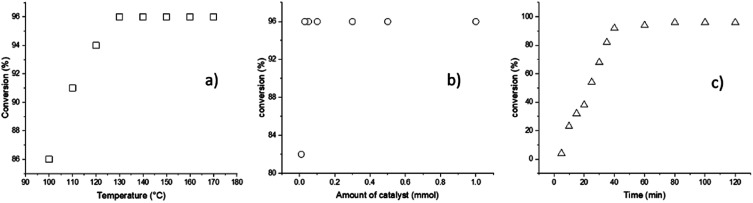
ROP of l-LA catalyzed by BiSS showing (a) the effect of temperature (2 h, 1 mmol BiSS), (b) the effect of the amount of catalyst used (140 °C, 2 h), and (c) the effect of the reaction time (140 °C, 0.03 mmol).

According to the previous descriptions, a different series of reaction parameters were tested, such as temperature, amount of catalyst, and reaction time. The factor used to quantify the optimal conditions of each parameter was the conversion, as detected by ^1^H NMR spectroscopy (Fig. 1).

The first parameter tested was the temperature, and variation of the temperature from 100 to 170 °C indicated an optimum value at 140 °C (Fig. 1a); testing of the second parameter, the amount of catalyst, is illustrated in the Fig. 1b, whereby the minimum amount of BiSS needed for full completion was 0.03 mmol. It is critical to minimize the concentration of the catalyst in the reaction medium for three reasons: (a) high catalyst concentration decreases the purity of the polyester, (b) high catalyst concentration can potentially induce side reactions, such as transesterification, and (c) low catalyst use leads to lower synthetic cost. The third parameter, reaction time, indicated that and excellent conversion was reached after 80 minutes (Fig. 1c). The optimal conditions of the ROP of l-LA in the presence of HO(CH_2_)_5_OH were ultimately found to be *T* = 140 °C, 0.03 mmol of BiSS, and 80 minutes.

**Fig. 2:**
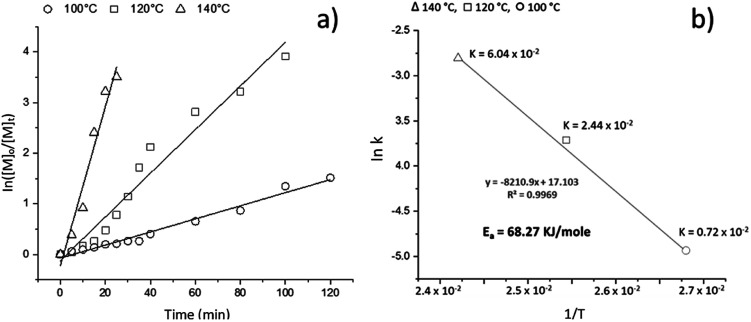
(a) Semilogarithmic plot of the ROP of l-LA catalyzed by BiSS concentration against the reaction time at different temperatures: *k* = 2.10 × 10^−4^ s^−1^ at 100 °C; *k* = 6.52 × 10^−4^ s^−1^ at 120 °C; *k* = 2.34 × 10^−3^ s^−1^ at 140 °C. (b) Graph of ln *k vs.* 1/*T* for the ROP of l-LA.

To understand the kinetics of the ROP of l-LA catalyzed by BiSS and initiated by 1,5-pentanediol, three similar experiments were undertaken, varying the temperature from 100 to 140 °C. In Fig. 2a, the profile of the kinetics indicates an excellent agreement to a linear dependency, suggesting a first-order reaction with respect to monomer. Additionally, the temperature had a proportional relationship to the consumption of l-LA: the rate constant increased from 100 to 140 °C. By calculating the constants, the activation energy (*E*_a_) was also obtained (Fig. 2b). The *E*_a_ of our reaction was found to be similar to previous reports of ROP of l-LA using stannous octoate [Sn(Oct)_2_] as catalyst (Table 2), such as the work of Eenink,^65^ Witzke^66^ and Puaux.^67^ Therefore, the BiSS has similar suitability for use as a catalyst to the commonly-employed catalyst Sn(Oct)_2_, but with the advantages of having low toxicity and environmental impact.

**Table 2:** Values of activation energy obtained in this work and other kinetic studies of the polymerization of l-lactide (l-LA)

Temperature (°C)	Activation energy (kJ mol^−1^)	Ref.
100–140	68.3	This work
103–130	80.6	54
130–220	70.9	55
170–195	79.8	56

α,ω-Hydroxy telechelic poly(l-lactide)s (HOPLLAOH) (HO–PLLA–(CH_2_)_*m*_–PLLA–OH) were prepared by ROP of l-LA catalyst by bismuth subsalicylate (BiSS) with a family of linear aliphatic diols as initiators. A gradual increase in the number of methylene groups in the diol (HO–[CH_2_]_*m*_–OH, *m* = 2, 3, 4, 5, 6, and 8) was selected and after 80 min at 140 °C (Scheme 2) an excellent conversion was obtained for all samples (*ca.* 96%, Table 3, HOPLLA_2−8_OH), which indicates that the conversion is not dependent on the number of methylene groups in the initiator. The conversion from l-LA to HOPLLAOH macrodiols in this work is comparable to the synthesis of α,ω-hydroxy telechelic PLLAs (HOPLLA–*x*–PLLAOH, where *x* = alkyl or ether groups) (yield: *ca.* 96%) previously reported using tin octoate (SnOct_2_) as catalyst.^68^

**Table 3:** Poly(l-lactide) macrodiols (HO–PLLA–[CH_2_]_*m*_–PLLA–OH) synthesized using different types of aliphatic diols [HO–(CH_2_)_*m*_–OH, where *m* = 2, 3, 4, 5, 6, and 8] as initiators in the ROP of l-LA

Sample	Initiator	Alkyl^a^ (%)	Conv.^b^ (%)	DP_NMR_^b^	*M*_n_ (theo)^c^	*M*_n_ (NMR)^b^^,^^d^	*M*_n_ (SEC)^e^	*Ð*_M_ e	Ratio^f^	*T*_g_ g
HOPLLA_2_OH	HO–[CH_2_]_2_–OH	4.6	96	17.8	1500	1340	2198	1.12	0.68	17
HOPLLA_3_OH	HO–[CH_2_]_3_–OH	5.4	96	18.7	1520	1420	2118	1.09	0.72	16
HOPLLA_4_OH	HO–[CH_2_]_4_–OH	6.4	96	18.4	1530	1410	2407	1.09	0.64	15
HOPLLA_5_OH	HO–[CH_2_]_5_–OH	7.4	96	18.0	1540	1400	2372	1.06	0.65	12
HOPLLA_6_OH	HO–[CH_2_]_6_–OH	8.4	96	18.8	1560	1470	2484	1.09	0.63	9
HOPLLA_8_OH	HO–[CH_2_]_8_–OH	9.7	96	18.8	1590	1500	2500	1.09	0.64	7
HOPDLLA_3_OH	HO–[CH_2_]_3_–OH	5.2	96	19.6	1510	1490	2541	1.09	0.59	12
HOPDLLA_8_OH	HO–[CH_2_]_8_–OH	9.6	96	19.1	1590	1520	2487	1.08	0.64	6

a Obtained from the equation alkyl (%) = (MW_initiator_/*M*_n_ (NMR)) × 100. Where MW_initiator_ is the molecular weight of initiator or alkyl diol (HOROH).

b The conversion was determined by ^1^H NMR spectroscopy in CDCl_3_.

c Calculated from the equation *M*_n_ (calcd) = (MW(M)) × (M/HOROH) + MW (HOROH), where MW is the molecular weight of the monomer (M = l-LA, 144 g mol^−1^) or initiator (HOROH).

d Calculated from the equation *M*_n_ (NMR) = (DP_PLLA_ × MW (repetitive unit)) + MW (HOROH), where MW is the molecular weight of the repeat unit (72 g mol^−1^) or aliphatic diol (HOROH).

e Determined by gel permeation chromatography (SEC) using polystyrene standards and corrected by factor 0.58.^69^

f *M*_n_ (theo)/*M*_n_ (GPC) ratio.

g Obtained by DSC analysis.

**Fig. 3:**
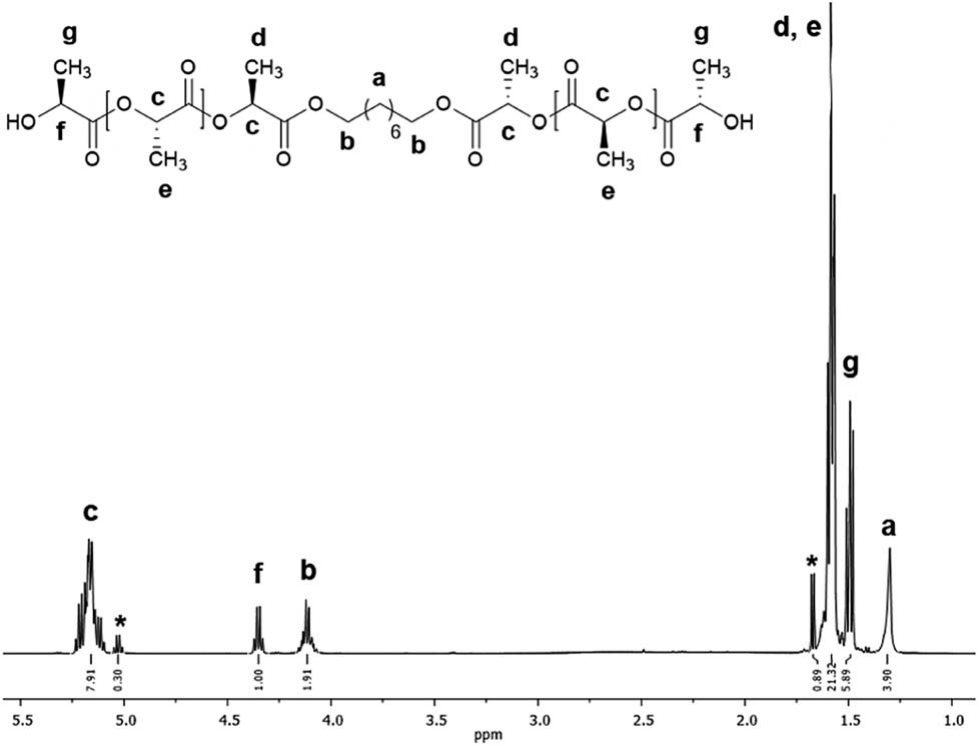
^1^H NMR spectrum at room temperature of HOPLLA_8_OH with DP_NMR_ = 18.8 (500 MHz, CDCl_3_); asterisks indicate the residual monomer (l-LA).

**Fig. 4:**
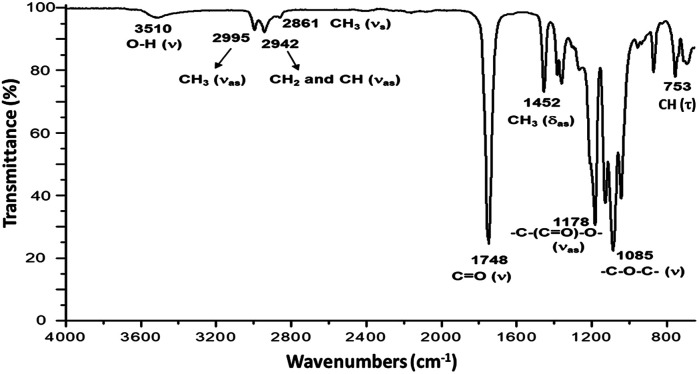
FT-IR spectrum of HOPLLA_8_OH.

**Fig. 5:**
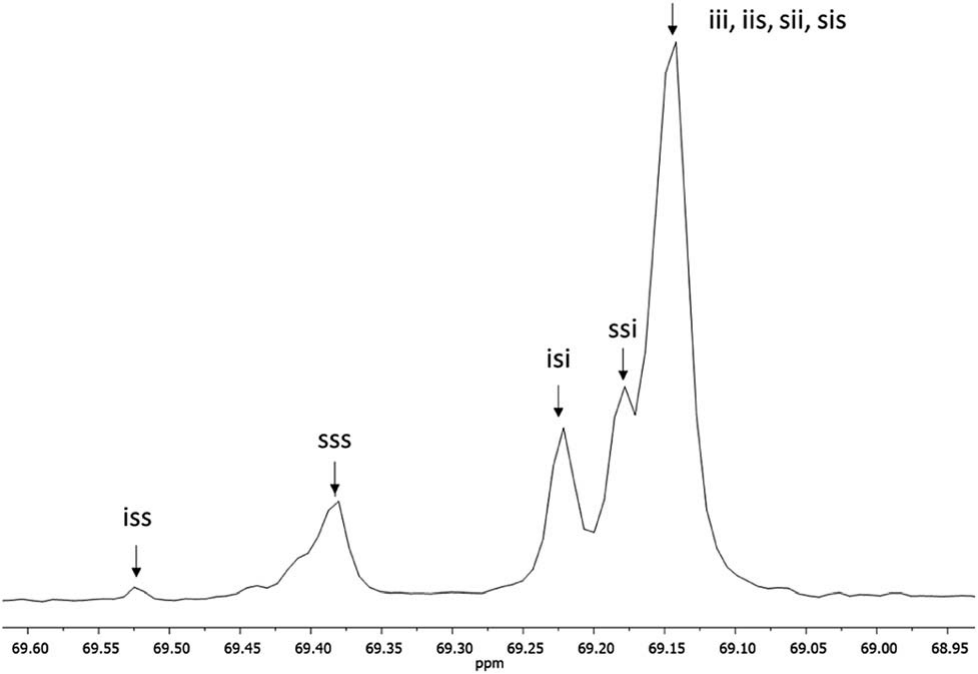
^13^C NMR spectrum of methine (CH) carbon, tetrad analysis of tacticity of HOPLLA_5_OH in CDCl_3_, where *i* and *s* correspond to isotactic and syndiotactic, respectively.

Fig. 3 shows the ^1^H NMR spectrum of HOPLLA_8_OH. The signals at 4.25 and 4.12 ppm are attributed to a methine group (CHOH) attached to the terminal hydroxyl group and the methylene group adjacent to the ester group (CH_2_OCO), evidence that the polymer is an α,ω-hydroxy telechelic polyester and that the aliphatic diol used as initiator had been inserted into the main chain. The rest of the signals are assigned to the repeat unit of the polyester and methylene groups of the initiator, the assignment of all peaks being consistent with those of reported α,ω-hydroxy telechelic PLLA samples.^70–72^ Additionally, the FT-IR spectrum (Fig. 4) evidenced a series of characteristic bands, such as the hydroxyl (O–H, 3510 cm^−1^) and carbonyl groups (C=O, 1748 cm^−1^), corroborating the chemical nature of the HOPLLAOH. However, the tacticity of HOPLLA_5_OH detected by ^13^C NMR (Fig. 5) shown a pattern of an atactic sample, this result is consistent with racemization of PLLA and PDLA previously reported by Asakura *et al.*^73^ So, during the polymerization to obtain PLLA oligomers the BiSS was not only a catalyst in the ROP of l-LA, but also induced the racemization of the polyester. The physical aspect of HOPLLA_5_OH is translucent; similar results were found for the rest of HOPLLAOH samples. Currently, more experiments to understand this effect is underway in our laboratory.

In the reactions summarized in Table 3, the feed l-LA/HOROH molar ratio applied was 10. However, as each lactone contains two ester groups, the theoretical ratio (DP_theo_) is 20. The degree of polymerization (DP_NMR_) was calculated experimentally using ^1^H-NMR spectroscopy (Fig. 3) and analysis of the terminal groups, providing a range of DP_NMR_ = 17.6–19.6 for our samples of HOPLLAOH. These values show excellent agreement with the theoretical ratio (DP_theo_ = 20), an effect that can be attributed to the transfer reaction between the aliphatic diol (HOROH) as initiator and the BiSS as a catalyst, which ultimately generates a new alkoxide (Bi–O–R–OH) that is the active species in the initiation of the ROP of l-LA. Thus, the content of HOROH (mmol) must be higher than BiSS in order to displace the equilibrium to favor a Bi–O–R–OH species, according to Le Chatelier's principle. The insertion of the alkyl group and the formation of the α,ω hydroxyl telechelic terminal groups, previously evidenced by ^1^H NMR (Fig. 3) and ^13^C NMR (Fig. S22†) confirmed the operation of this transfer reaction.

The values of the number-average molecular weight (*M*_n_) calculated by SEC are higher than the *M*_n_ values obtained by NMR analysis (Table 3). This effect is attributed to the differences in the hydrodynamic radius between the polystyrene standards and the HOPLLAOH samples. The dispersity (*Ð*_M_) of HOPLLAOH samples was found to be narrow (1.06–1.12), with a unimodal distribution (Fig. S24†), suggesting that the initiation of the ROP is faster than the propagation. In line with this, experiments using a different monomer, such as d,l-lactide (racemate) (HOPDLLA_3−8_OH, Table 3), demonstrated similar behavior to the ROP of l-LA by BiSS. The synthesis of the HOPLLAOH with high *M*_n_ catalysed by BiSS will be explored in a future contribution.

**Fig. 6:**
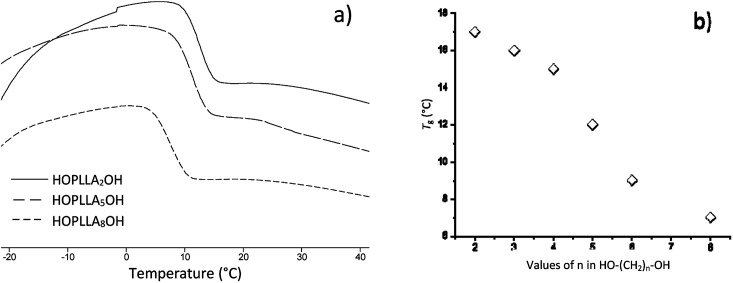
(a) DSC thermograms of the poly(l-lactide) macrodiols: HOPLLA_2_OH, HOPLLA_5_OH, HOPLLA_8_OH, and (b) the effect of multiple methylene groups (CH_2_)_*n*_ on the glass transition temperature (*T*_g_).

The thermograms of different samples of HOPLLAOH are illustrated in Fig. 6a. A melting point (*T*_m_) was not observed during the acquisition of the thermograms; therefore, all HOPLLAOH samples are expected to be amorphous. Accordingly, all polyester samples produced herein had a translucent appearance. On the other hand, the unique transition visualized in the HOPLLAOH samples was the glass transition temperature (*T*_g_), and the size of the alkyl group had a significant effect on the *T*_g_ values. For example, the value of *T*_g_ decreased from 17 to 7 °C upon going from HOPLLA_2_OH (with two methylene groups) to HOPLLA_8_OH (with eight methylene groups). Thus, the number of methylene groups was inversely proportional to the *T*_g_ values, as seen in Fig. 6b, indicating that longer alkyl groups favor flexibility in the macromolecule. Similar behavior was reported by Báez *et al.*^68^ HOPLLAOH is thus an attractive candidate for use in block copolymers, potentially allowing the modulation of the amorphous domains; experiments are currently underway in our laboratory to explore this possibility.

### Mechanism of initiation

**Scheme 3:**
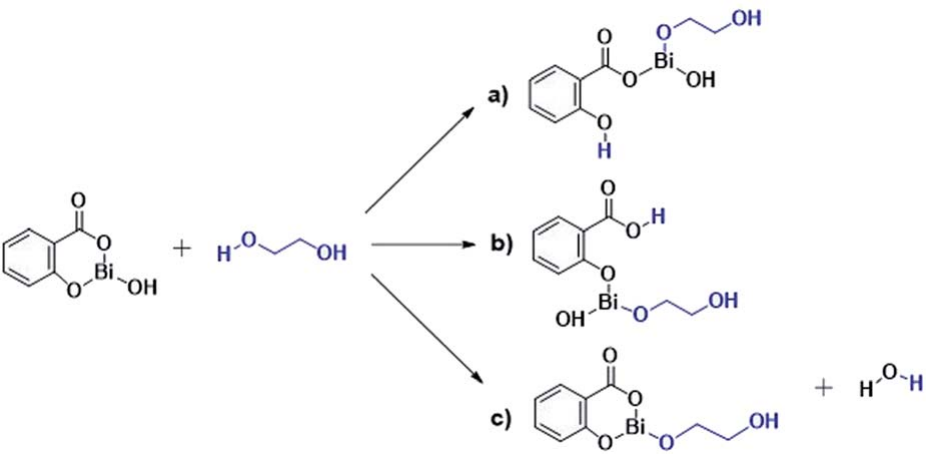
Transfer reaction between BiSS and ethylene glycol.

The results presented herein have demonstrated the abilities of BiSS as a catalyst in the ROP of l-LA, and the insertion of an alkyl diol [HO(CH_2_)_*n*_OH] into the main chain of the polyester (producing an ester group) demonstrated the role of these diols as initiators. The experimental evidence suggests that a bismuth alkoxide (Bi–OR) is formed *in situ* by a transfer reaction, and eventually, Bi–OR can act as an active species during the initiation step. In a previous report, Lahcini *et al.*^29^ used a series of bismuth(iii) alkoxide catalysts for the ROP of lactones, and found that bulkier bismuth alkoxides showed high activity. In our case, the BiSS has three possibilities to produce the Bi–OR species by a transfer reaction (Scheme 3): (1) generally, a metallic hydroxy species (Scheme 3c) such as Bi–OH is insufficiently basic in an organic medium to deprotonate an alcohol (HOR) (or a diol HOROH) and generate Bi–OR (or Bi–OROH) and water, and (2) the bidentate ligand derived from 2-hydroxy-benzoic acid (salicylic acid) bound to the Bi center has two different types of Bi–O bonds, *i.e.* to phenoxy and benzoate groups.

The difference in p*K*_b_ values between the phenoxy ion (0.4) and benzoate ion (11.03) is significant,^74^ thus it can be expected that the bond between the phenoxy group and the Bi center (PhO–Bi) can act as a Lewis base to deprotonate the alkyl diol and produce the Bi–OROH species plus a phenol group (Scheme 3a). To corroborate this idea, a series of computational calculations were conducted by DFT-based calculations at the PBE0/[LANL2DZ-6-31G(d)] level of theory (see Computational details). Table 4 lists the energy barriers to produce a transfer reaction, whereby the formation of a bismuth alkoxide and a phenol group is favored. Additionally, the effect of the numbers of methylenes (CH_2_)_*n*_ from ethylene glycol [HO(CH_2_)_2_OH] to 1,5-pentanediol [HO(CH_2_)_5_OH] on the transfer reaction (Scheme 3, Table S1†) exhibited a gradual increase in the total barrier of the alkoxide, and also the product (a) (Scheme 3a) is mainly favored concerning (c) (Scheme 3c).

**Table 4:** Key reaction energies of the possible alkoxides formed from the reaction between bismuth subsalicylate (BiSS) and ethylene glycol [HO(CH_2_)_2_OH] (transfer reaction, Scheme 3)

Alkoxides	Total energy barrier (kcal mol^−1^)	Activation energy (*E*_a_, kcal mol^−1^)
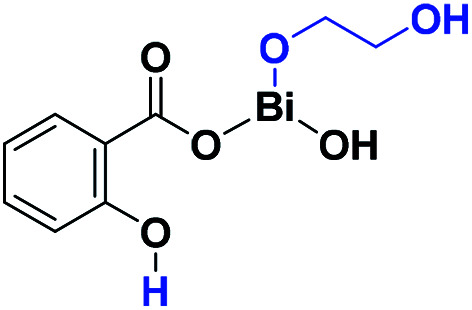	13.57	6.85
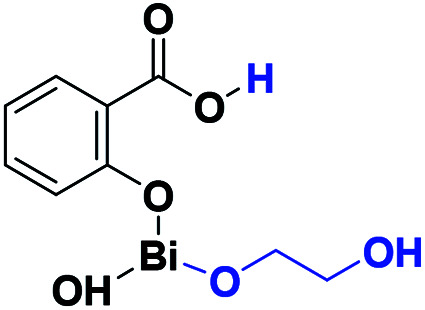	30.89	12.78
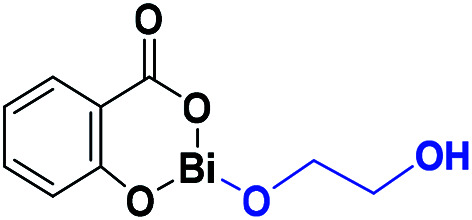	41.56	35.16

**Fig. 7:**
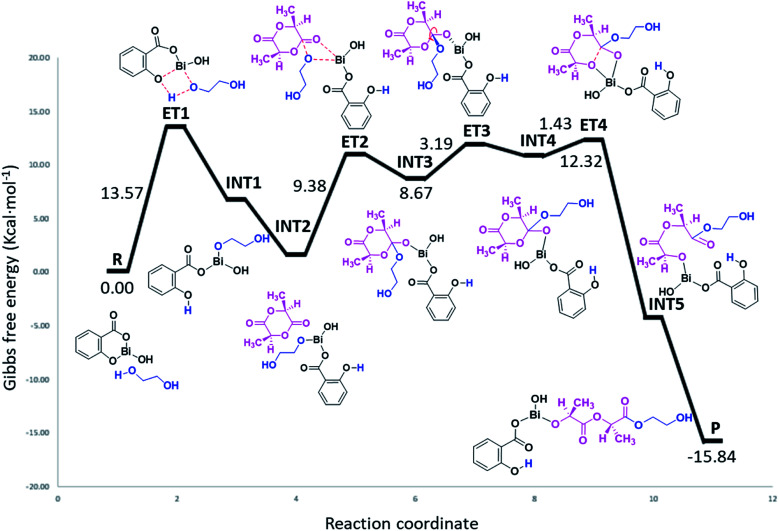
Energy profile (gas phase, 140 °C) of the mechanism of initiation of l-lactide (l-LA) initiated with ethylene glycol [HO(CH_2_)_2_OH] using bismuth subsalicylate (BiSS) as a catalyst.

As can be seen in Fig. 7, the polymerization occurs through a ring-opening mechanism. Initially, the reaction is promoted by the formation of a metal alkoxide (Bi–OROH) product of the reaction between the BiSS catalyst and the ethylene glycol initiator. This induction step is the rate-determining step of the polymerization of l-LA, with an energy barrier of 13.57 kcal mol^−1^. The reaction continues by coordination of the carbonyl group of l-LA with the Bi–O bond of the bismuth alkoxide, with an energy barrier of 9.38 kcal mol^−1^. This followed by the insertion of the alkoxide group INT3, and then a rearrangement of the intermediate INT3, favoring the opening of the ring, which consists of a rotation of the substituents (alkoxy and ethoxy) of almost 90°.

The energy barrier of this rearrangement, which leads to the formation of the intermediate INT4, is 3.19 kcal mol^−1^, and this shallow barrier suggests that both intermediates are likely in equilibrium. The next reaction step is the opening of the ring and occurs with a very small energy barrier of 1.43 kcal mol^−1^. The last step proposed in this mechanism is a rearrangement toward zigzag conformation of INT5 to obtain P due to steric issues; these occur through a transition state similar to ET3.^75^ The formation energy of P is −15.84 kcal mol^−1^ making it an exergonic (spontaneous) reaction.

## Conclusions

Bismuth subsalicylate (BiSS) acts as a catalyst in the ring-opening polymerization (ROP) of l-lactide (l-LA) in the presence of linear aliphatic diols [HO(CH_2_)_*n*_OH, where *n* = 2, 3, 4, 5, 6, and 8] as initiators, obtaining α,ω-hydroxy telechelic poly(l-lactide) (HOPLLAOH) in excellent conversion. A good agreement of the measured and theoretical degrees of polymerization (DP) in all HOPLLAOH samples was evidenced. This effect is attributed to the interaction of BiSS with HO(CH_2_)_*n*_OH, which induces a transfer reaction and, ultimately, generates an active bismuth alkoxide (Bi–OROH) species. The rate constant for the ROP of l-LA catalyzed by BiSS was found to be proportional to the temperature, and the activation energies are comparable to those of the conventionally-used catalyst tin octoate [Sn(Oct)_2_]. A computational study elucidated the behavior of the BiSS in the presence of an alkyl diol (HOROH), producing a Bi–OROH that acts as an active species and nucleophile in the ring-opening of l-LA. These results indicate that BiSS is a very promising catalyst candidate for the ROP of l-LA due to its low cost, low toxicity, and efficacy in the synthesis of macrodiols such as HOPLLAOH, which in turn are key precursors to triblock copolymers and poly(ester-urethanes).

## Conflicts of interest

There are no conflicts to declare.

## Supplementary Material

RA-010-D0RA05413E-s001
