# Supplementary material for: Bismuth subsalicylate, a low-toxicity catalyst for the ring-opening polymerization (ROP) of l-lactide (l-LA) with aliphatic diol initiators: synthesis, characterization, and mechanism of initiation
Source: RSC Adv. 2020 Aug 20;10(51):30815–24. doi: 10.1039/d0ra05413e (PMC9057703; doi:10.1039/d0ra05413e)
Supplement: RA-010-D0RA05413E-s001 [file RA-010-D0RA05413E-s001.pdf]

Electronic Supplementary Material (ESI) for RSC Advances.
This journal is © The Royal Society of Chemistry 2020

Supplementary Information

Bismuth subsalicylate, a low-toxicity catalyst for the ring-opening polymerization (ROP) of L-lactide (L-LA) with aliphatic diols initiators: Synthesis, characterization, and mechanism of initiation

María Guadalupe Ortiz-Aldaco, José E. Báez*, J. Oscar C. Jiménez-Halla

Department of Chemistry, University of Guanajuato (UG), Noria Alta S/N, 36050 Guanajuato, Gto., Mexico.

To whow correspondence should be addressed: José E. Báez, e-mail: jebaez@ugto.mx

Table of Contents

		Page
**^{1}H NMR spectra of Macrodiols (HOPLLAOH) using alkyl diols (HO-$[CH_2]_m$-OH) and L-LA.**		**5**
	**Figure S1.** ^{1}H NMR spectrum of $HOPLLA_2OH$ (500 MHz, $CDCl_3$) derivatized with TFAA.	5
	**Figure S2.** ^{1}H NMR spectrum of $HOPLLA_3OH$ (500 MHz, $CDCl_3$) derivatized with TFAA.	5
	**Figure S3.** ^{1}H NMR spectrum of $HOPLLA_4OH$ (500 MHz, $CDCl_3$) derivatized with TFAA.	6
	**Figure S4.** ^{1}H NMR spectrum of $HOPLLA_5OH$ (500 MHz, $CDCl_3$) derivatized with TFAA.	6
	**Figure S5.** ^{1}H NMR spectrum of $HOPLLA_6OH$ (500 MHz, $CDCl_3$) derivatized with TFAA.	7
	**Figure S6.** ^{1}H NMR spectrum of $HOPLLA_8OH$ (500 MHz, $CDCl_3$) derivatized with TFAA.	7
	**Figure S7.** ^{1}H NMR spectrum of $HOPDLLA_3OH$ (500 MHz, $CDCl_3$) derivatized with TFAA.	8
	**Figure S8.** ^{1}H NMR spectrum of $HOPDLLA_8OH$ (500 MHz, $CDCl_3$) derivatized with TFAA.	8
	**Figure S9.** ^{1}H NMR spectrum of $HOPLLA_2OH$ (500 MHz, $CDCl_3$).	9
	**Figure S10.** ^{1}H NMR spectrum of $HOPLLA_3OH$ (500 MHz, $CDCl_3$).	9
	**Figure S11.** ^{1}H NMR spectrum of $HOPLLA_4OH$ (500 MHz, $CDCl_3$).	10
	**Figure S12.** ^{1}H NMR spectrum of $HOPLLA_5OH$ (500 MHz, $CDCl_3$).	10
	**Figure S13.** ^{1}H NMR spectrum of $HOPLLA_6OH$ (500 MHz, $CDCl_3$).	11
	**Figure S14.** ^{1}H NMR spectrum of $HOPLLA_8OH$ (500	11

	MHz, $CDCl_3$).	
	**Figure S15.** ^{1}H NMR spectrum of $HOPDLLA_3OH$ (500 MHz, $CDCl_3$).	12
	**Figure S16.** ^{1}H NMR spectrum of $HOPDLLA_8OH$ (500 MHz, $CDCl_3$).	12
**^{13}C NMR spectra of Macrodiols (HOPLLAOH) using alkyl diols (HO-$[CH_2]_m$-OH) and L-LA.**		**13**
	**Figure S17.** ^{13}C NMR spectrum of $HOPLLA_2OH$ (500 MHz, $CDCl_3$).	13
	**Figure S18.** ^{13}C NMR spectrum of $HOPLLA_3OH$ (500 MHz, $CDCl_3$).	13
	**Figure S19.** ^{13}C NMR spectrum of $HOPLLA_4OH$ (500 MHz, $CDCl_3$).	14
	**Figure S20.** ^{13}C NMR spectrum of $HOPLLA_5OH$ (500 MHz, $CDCl_3$).	14
	**Figure S21.** ^{13}C NMR spectrum of $HOPLLA_6OH$ (500 MHz, $CDCl_3$).	15
	**Figure S22.** ^{13}C NMR spectrum of $HOPLLA_8OH$ (500 MHz, $CDCl_3$).	15
**^{1}H-^{13}C HMBC NMR**		**16**
	**Figure S23.** ^{1}H-^{13}C HMBC NMR spectrum of $HOPLLA_8OH$ (500 MHz, $CDCl_3$).	16
**SEC chromatograms**		**16**
	**Figure S24.** SEC chromatograms profile for a) $HOPLLA_2OH$, b) $HOPLLA_8OH$.	16
**DSC thermograms**		**17**
	**Figure S25.** DSC thermograms of the different poly(L-lactide) macrodiols (HOPLLAOH) (all samples).	17
**Computational Details**		**18**
	**Scheme S1**. Energy profile in gas phase at 140°C of the ROP of the L-lactide initiated with ethylene	19

	glycol using bismuth subsalicylate as catalyst.	
	**Scheme S2**. Energy profile in gas phase at 140°C of the ROP of the L-lactide initiated with 1,3-propanediol using bismuth subsalicylate as catalyst.	20
	**Scheme S3**. Energy profile in gas phase at 140°C of the ROP of the L-lactide initiated with 1,4-butanediol using bismuth subsalicylate as catalyst.	21
	**Scheme S4**. Energy profile in gas phase at 140°C of the ROP of the L-lactide initiated with 1,5-pentanediol using bismuth subsalicylate as catalyst.	22
	**Table S1.** Total energy barrier using the following diols as initiators of the ROP of L-Lactide catalyst with bismuth subsalicylate.	18
	**Table S2.** Cartesian coordinates (xyz format) of the optimized geometries for all the species involved in the energy profile of the **Scheme S1** calculated at the D3-PBE0/[6-31G(d), LANL2DZ] level.	23
	**Table S3.** Cartesian coordinates (xyz format) of the optimized geometries for all the species involved in the energy profile of the **Scheme S2** calculated at the D3-PBE0/[6-31G(d), LANL2DZ] level.	30
	**Table S4.** Cartesian coordinates (xyz format) of the optimized geometries for all the species involved in the energy profile of the **Scheme S3** calculated at the D3-PBE0/[6-31G(d), LANL2DZ] level.	38
	**Table S5.** Cartesian coordinates (xyz format) of the optimized geometries for all the species involved in the energy profile of the **Scheme S4** calculated at the D3-PBE0/[6-31G(d), LANL2DZ] level.	46
**References**		**54**

^{1}H NMR spectra of Macrodiols (HOPLLAOH) prepared using different types of linear alkyl diols as initiators in the ROP of L-LA.

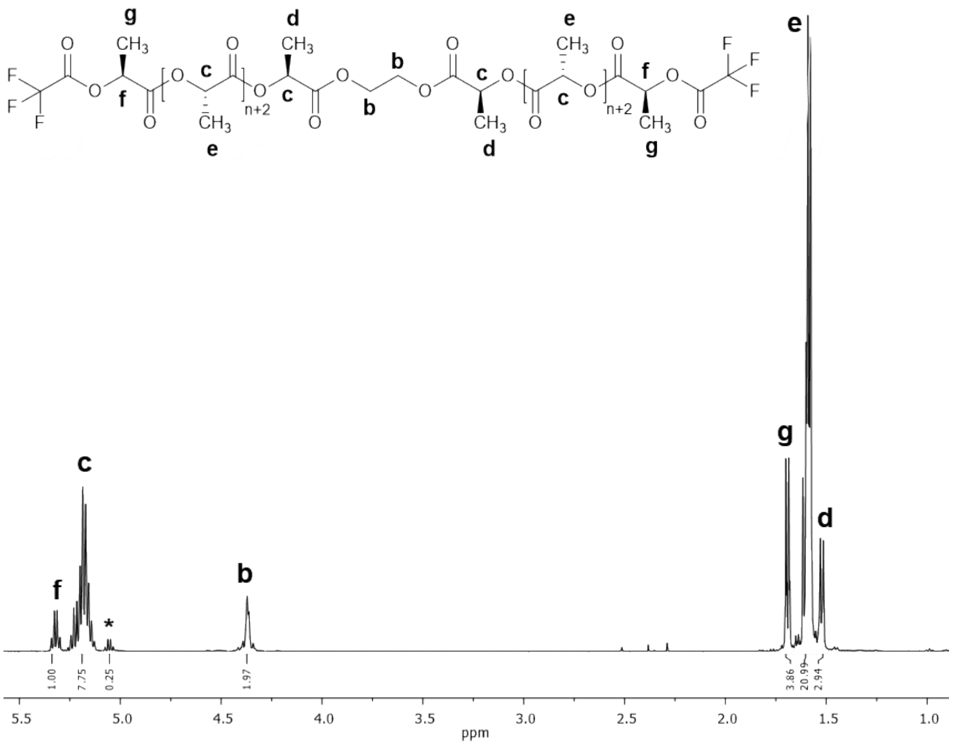

Figure S1. ^{1}H NMR spectrum of HOPLLA$_2$OH (500 MHz, $CDCl_3$) derivatized with TFAA.

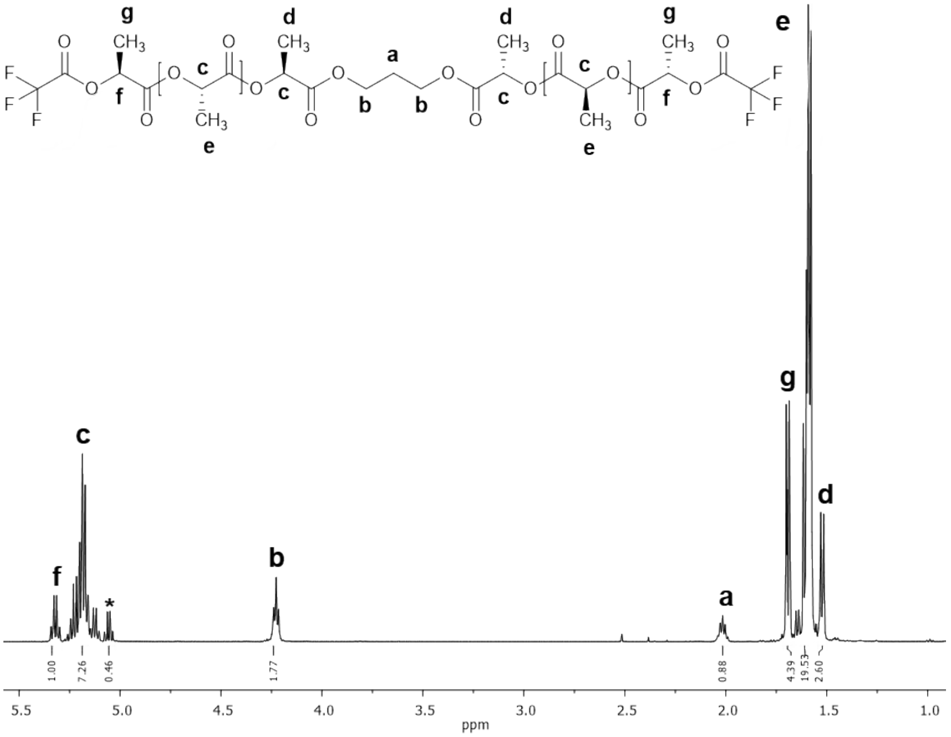

Figure S2. ^{1}H NMR spectrum of HOPLLA$_3$OH (500 MHz, $CDCl_3$) derivatized with TFAA.

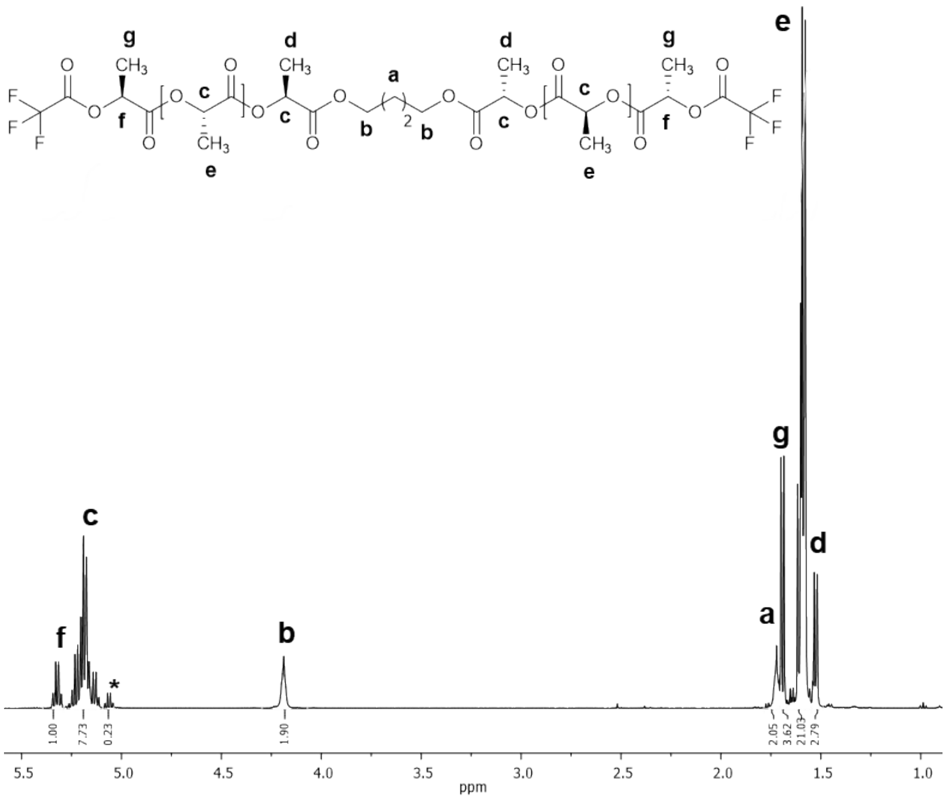

Figure S3. ^{1}H NMR spectrum of $HOPLLA_4OH$ (500 MHz, $CDCl_3$) derivatized with TFAA.

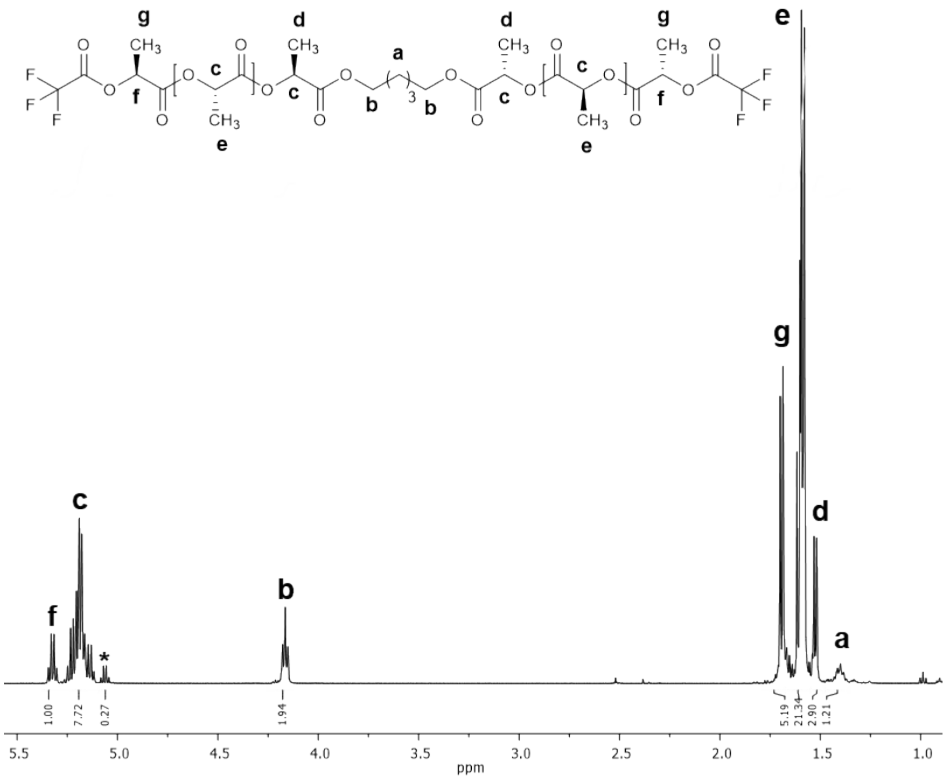

Figure S4. ^{1}H NMR spectrum of $HOPLLA_5OH$ (500 MHz, $CDCl_3$) derivatized with TFAA.

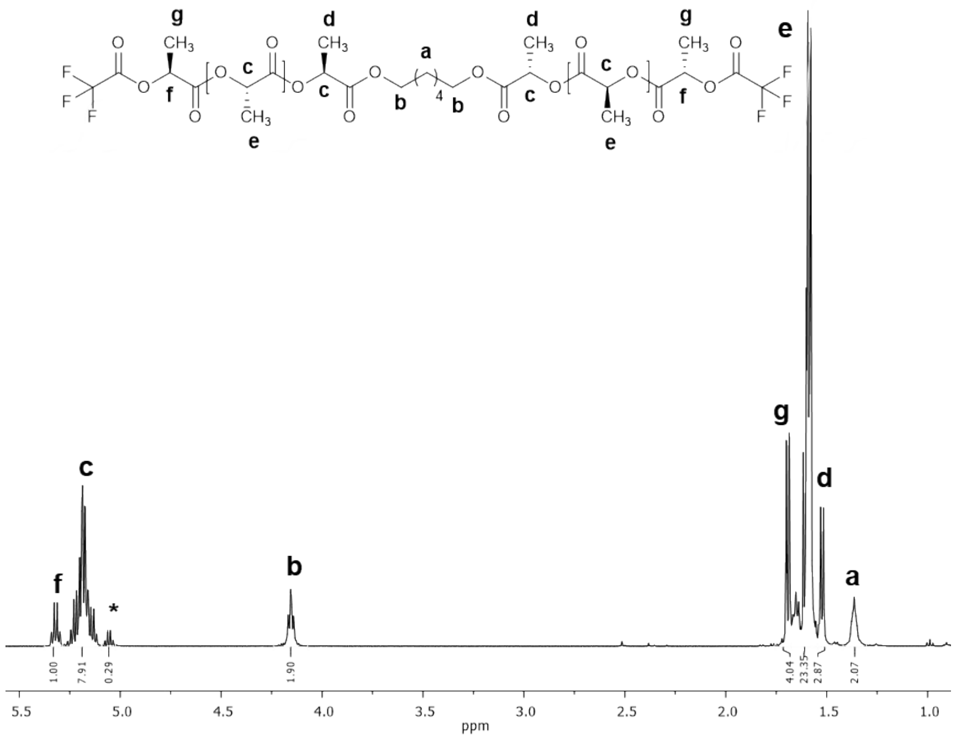

Figure S5. ^{1}H NMR spectrum of $HOPLLA_6OH$ (500 MHz, $CDCl_3$) derivatized with TFAA.

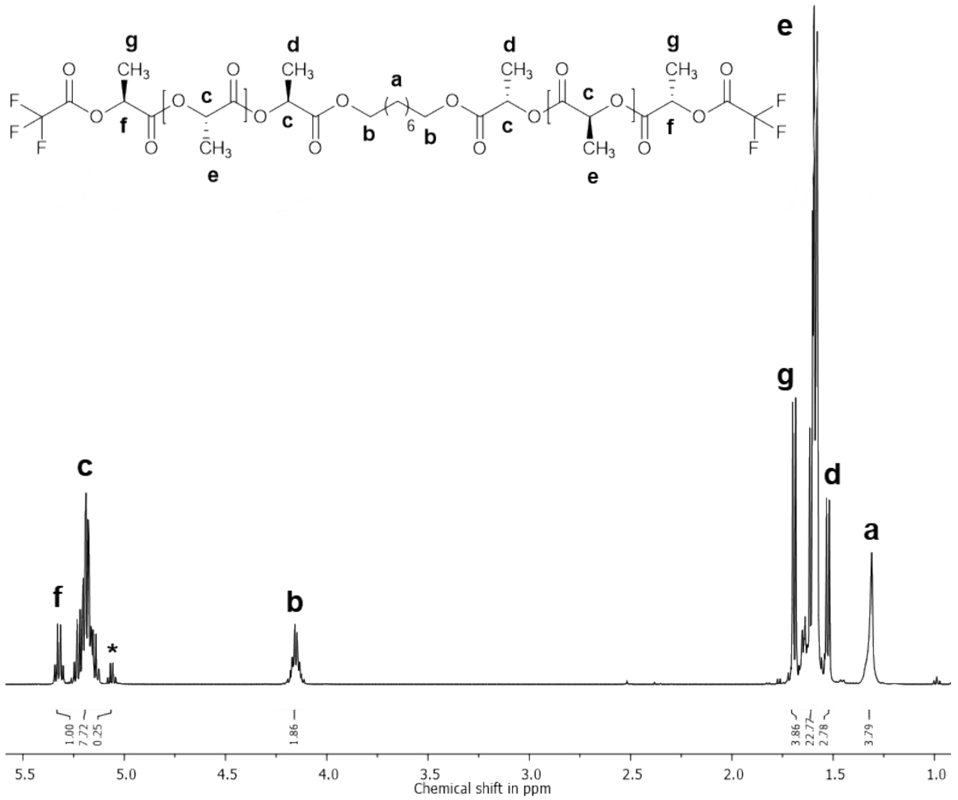

Figure S6. ^{1}H NMR spectrum of $HOPLLA_8OH$ (500 MHz, $CDCl_3$) derivatized with TFAA.

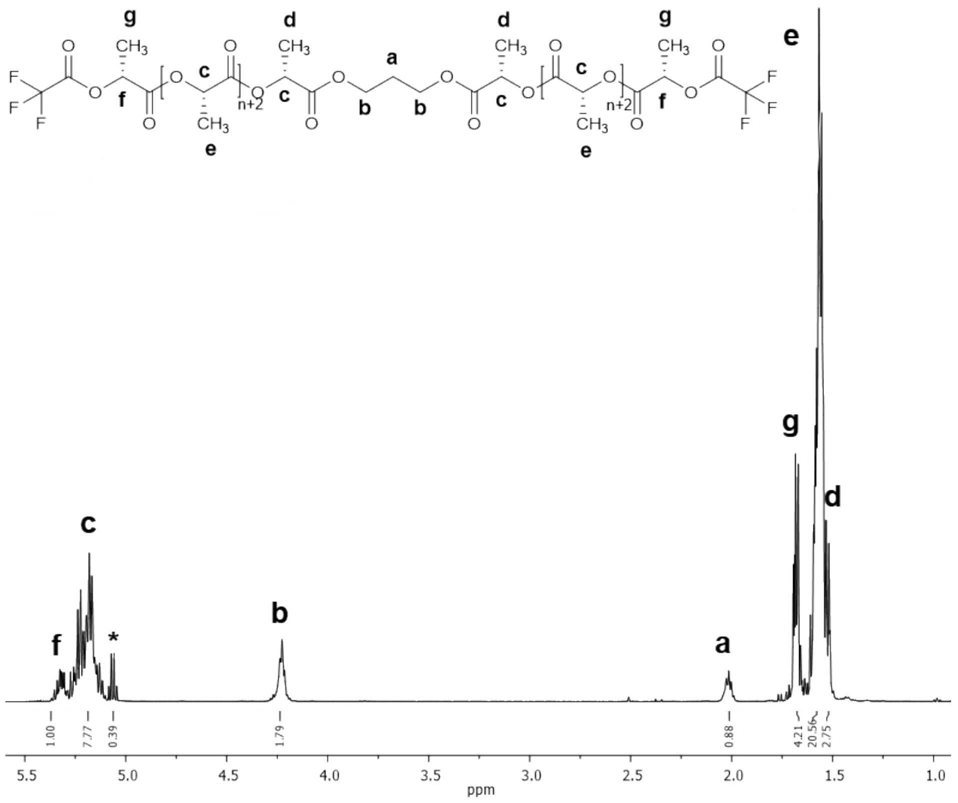

Figure S7. ^{1}H NMR spectrum of HOPDLLA$_3$OH (500 MHz, $CDCl_3$) derivatized with TFAA.

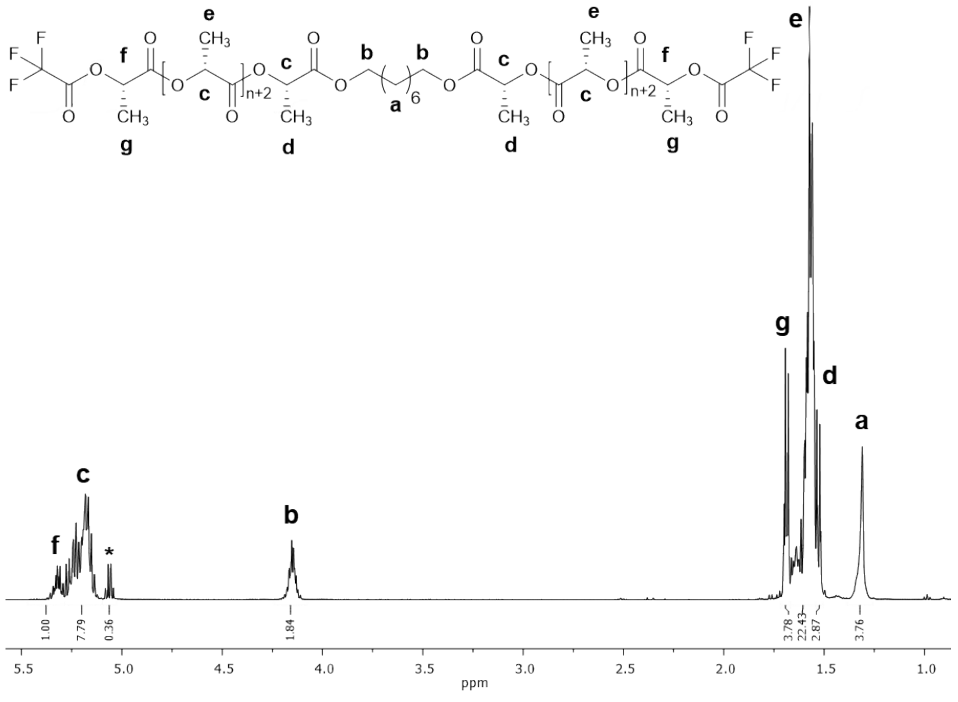

Figure S8. ^{1}H NMR spectrum of HOPDLLA$_8$OH (500 MHz, $CDCl_3$) derivatized with TFAA.

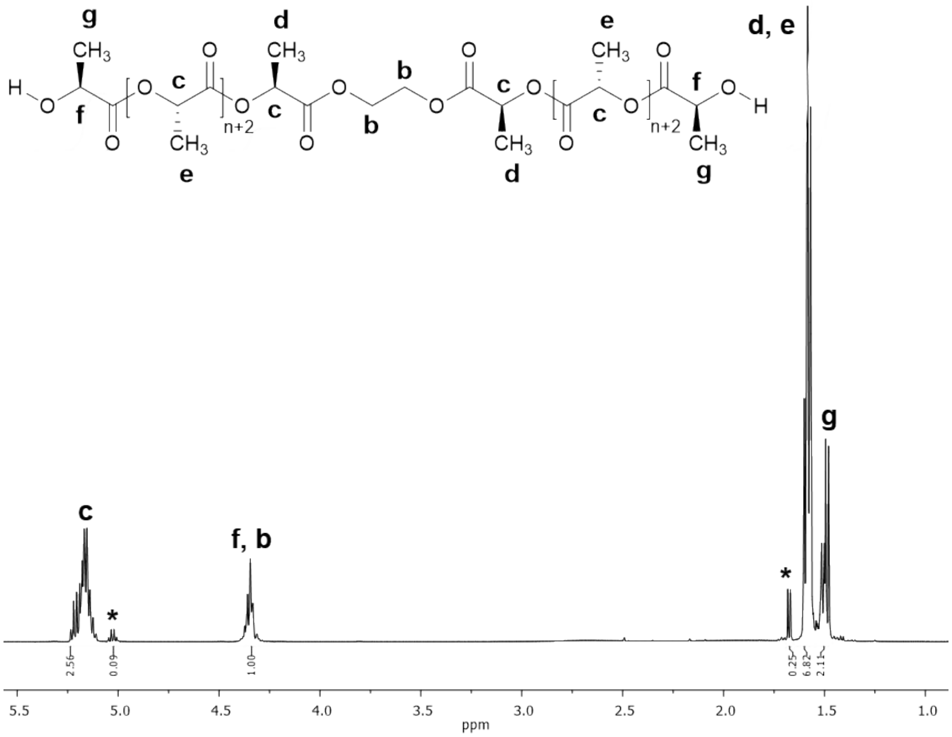

Figure S9. ^{1}H NMR spectrum of $HOPLLA_2OH$ (500 MHz, $CDCl_3$).

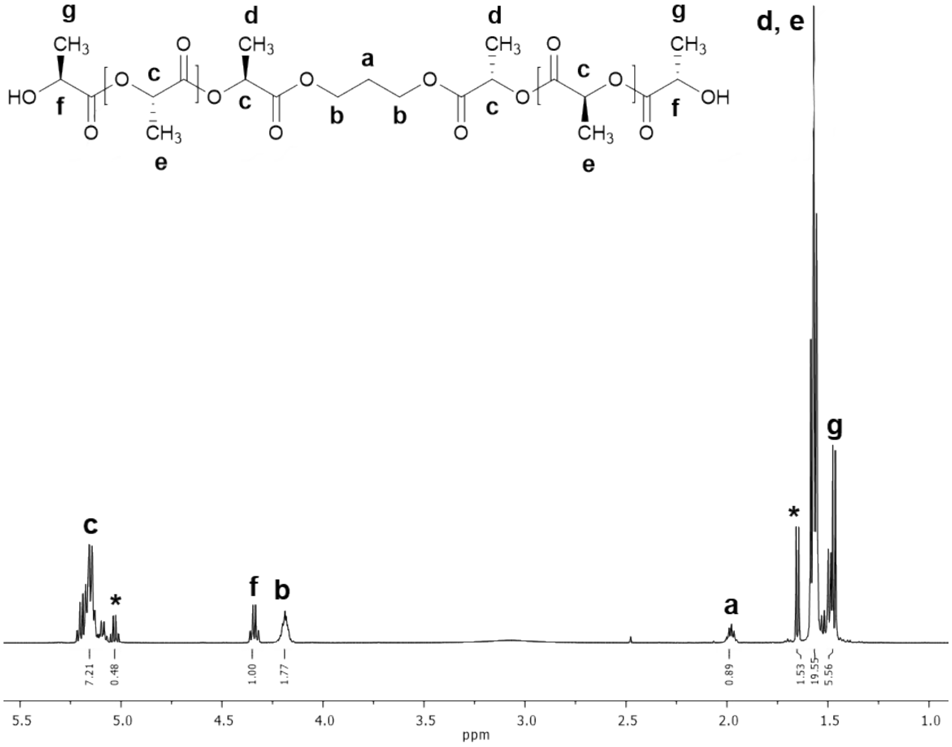

Figure S10. ^{1}H NMR spectrum of $HOPLLA_3OH$ (500 MHz, $CDCl_3$).

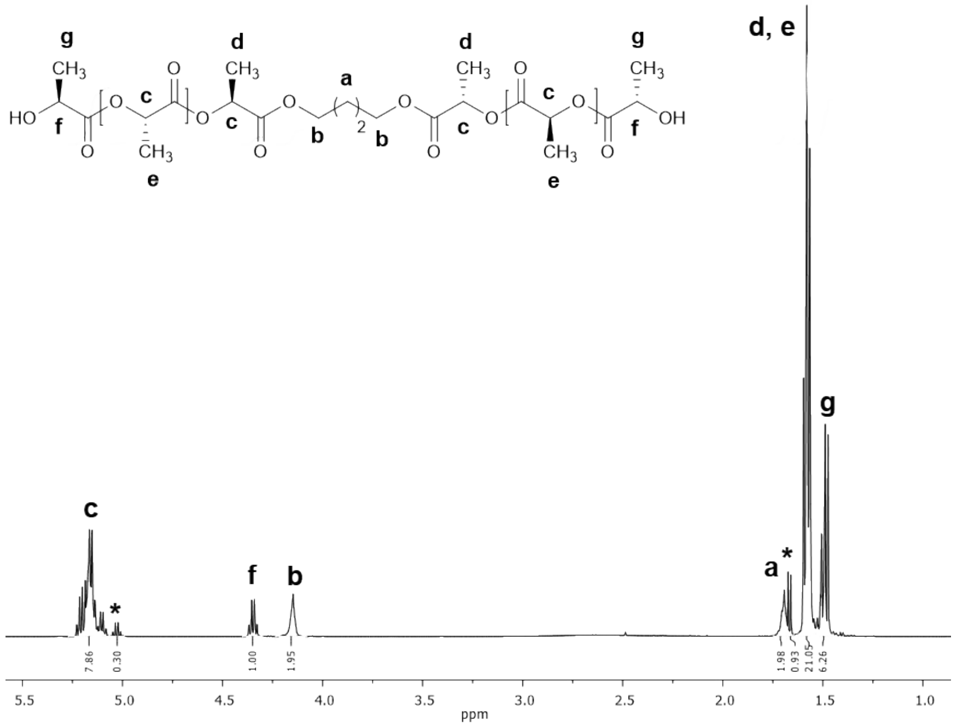

Figure S11. ^{1}H NMR spectrum of $HOPLLA_4OH$ (500 MHz, $CDCl_3$).

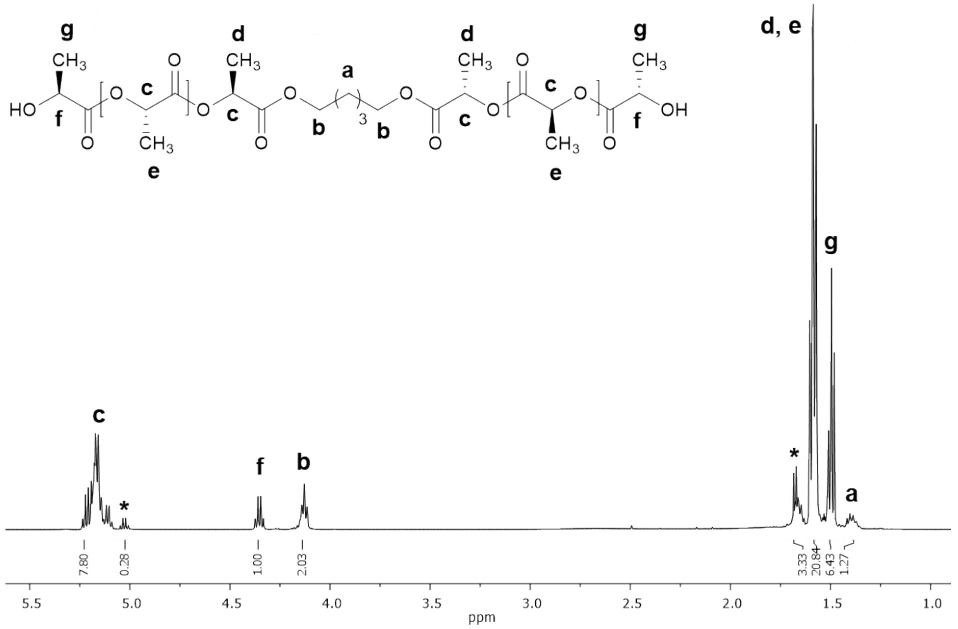

Figure S12. ^{1}H NMR spectrum of $HOPLLA_5OH$ (500 MHz, $CDCl_3$).

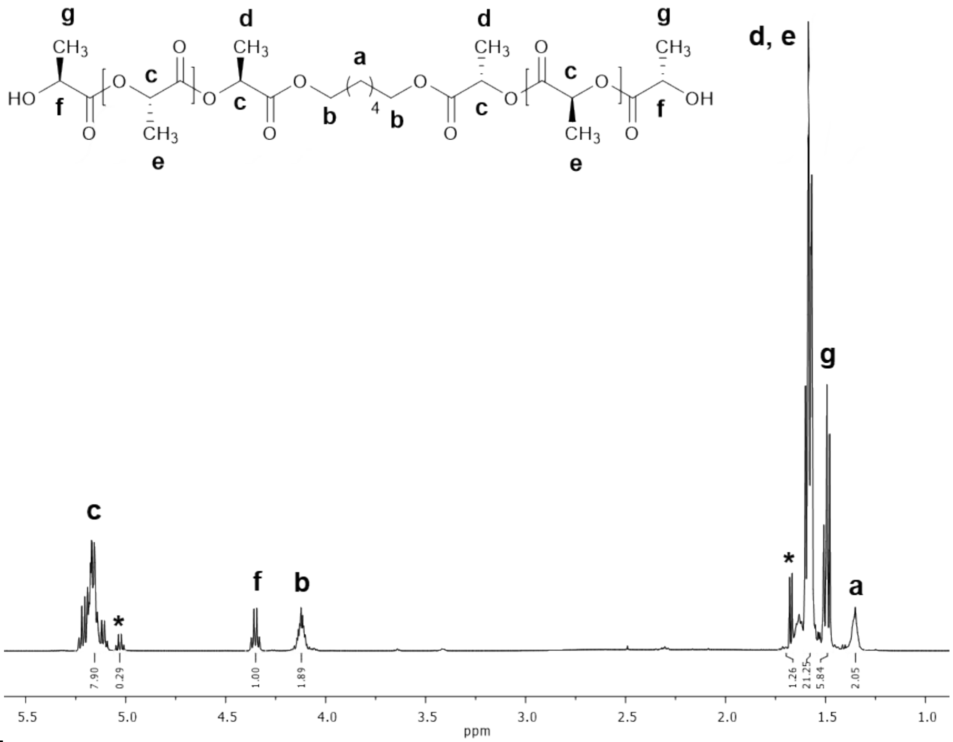

4

Figure S13. ^{1}H NMR spectrum of HOPLLA$_6$OH (500 MHz, CDCl$_3$).

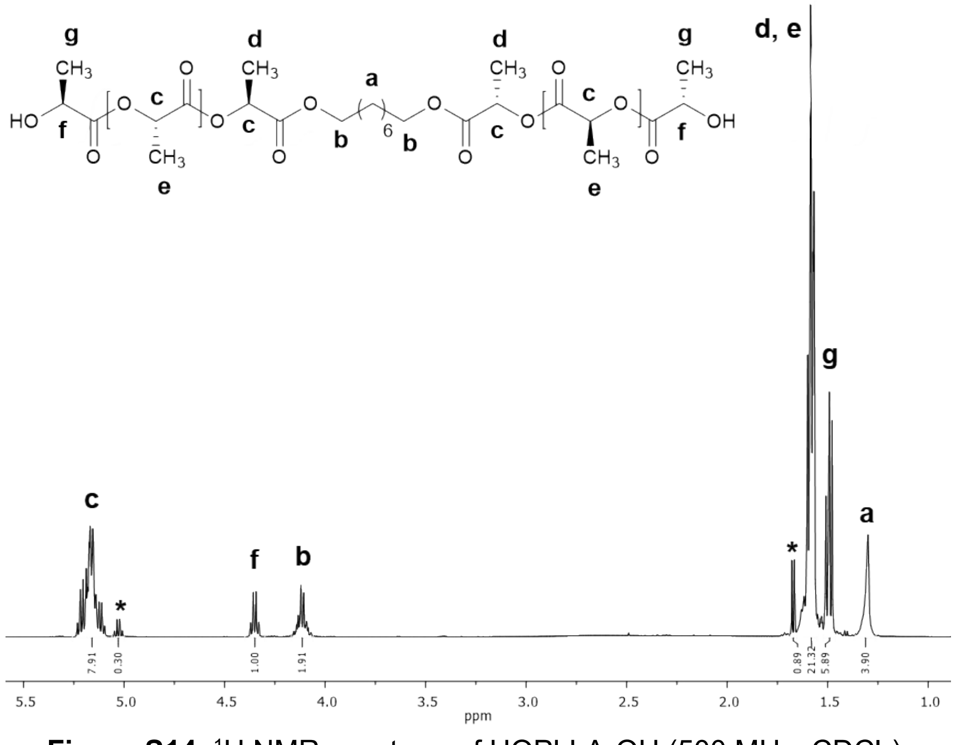

Figure S14. ^{1}H NMR spectrum of HOPLLA$_8$OH (500 MHz, CDCl$_3$).

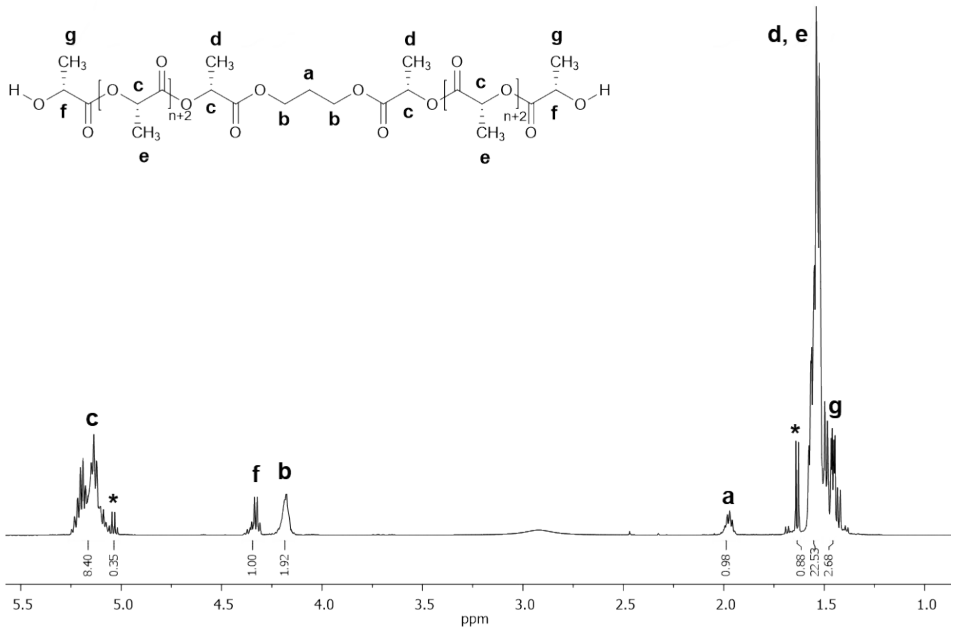

Figure S15. ^{1}H NMR spectrum of HOPDLLA$_3$OH (500 MHz, $CDCl_3$).

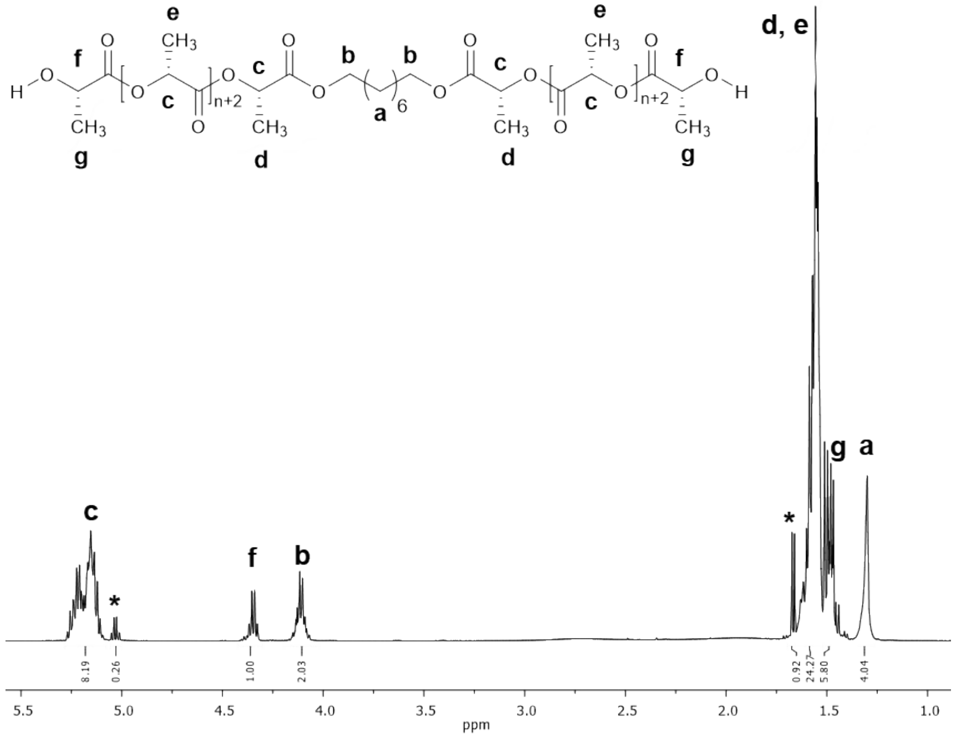

Figure S16. ^{1}H NMR spectrum of HOPDLLA$_8$OH (500 MHz, $CDCl_3$.

^{13}C NMR spectra of Macrodiols (HOPLLAOH) prepared using different types of linear alkyl diols as initiators in the ROP of L-LA.

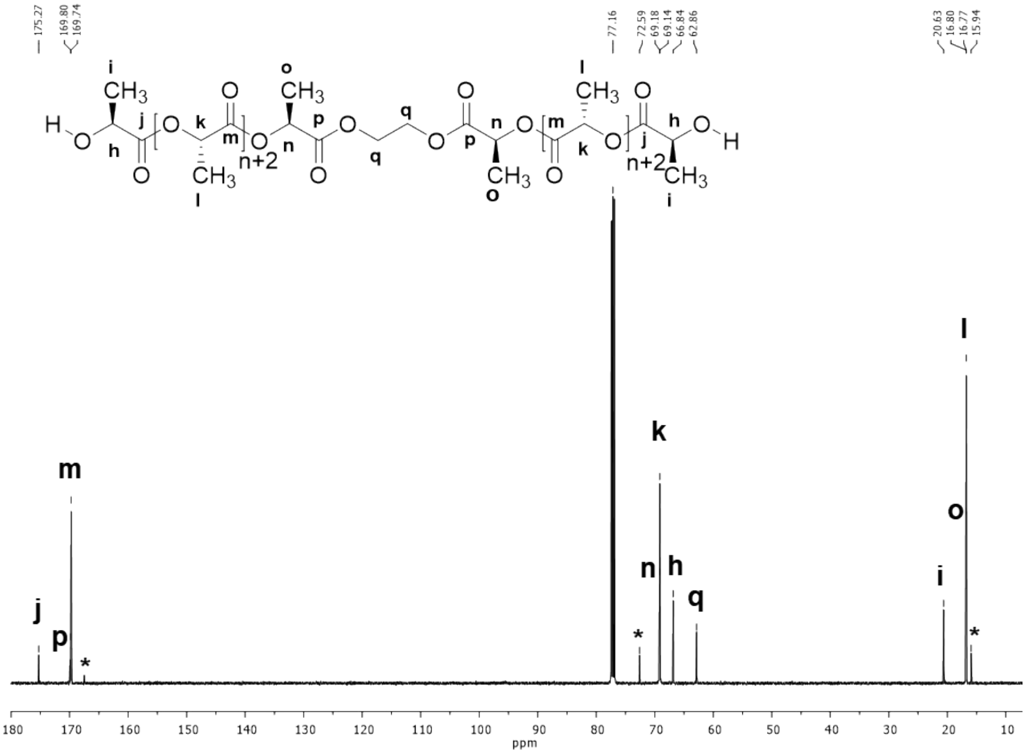

Figure S17. ^{13}C NMR spectrum of $HOPLLA_2OH$ (500 MHz, $CDCl_3$).

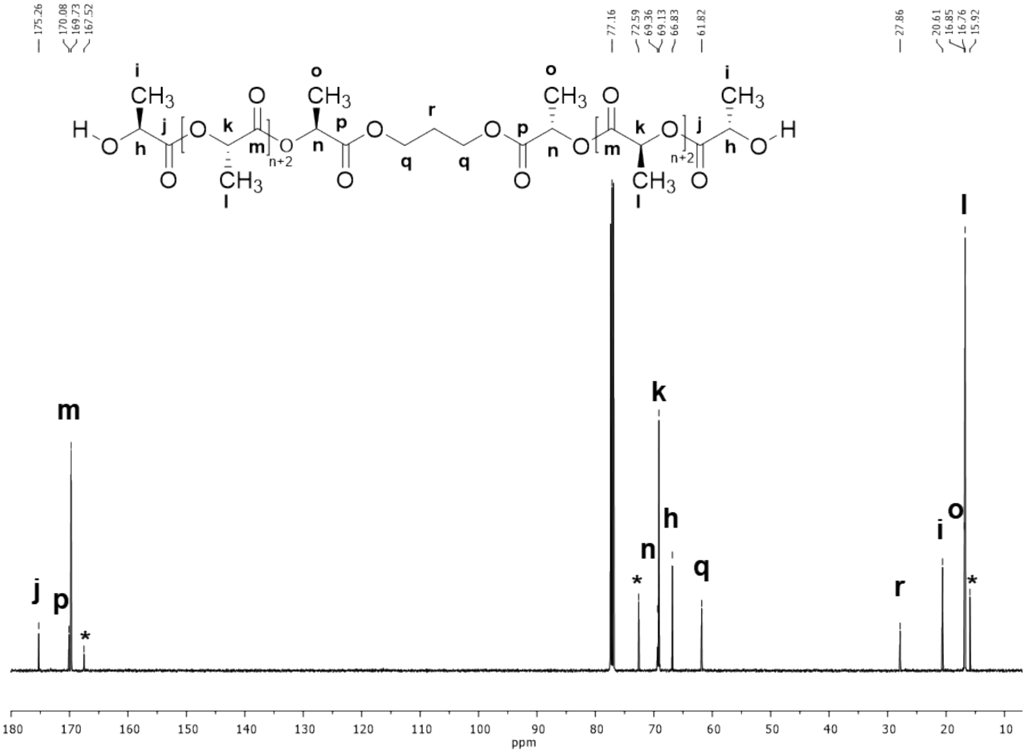

Figure S18. ^{13}C NMR spectrum of $HOPLLA_3OH$ (500 MHz, $CDCl_3$).

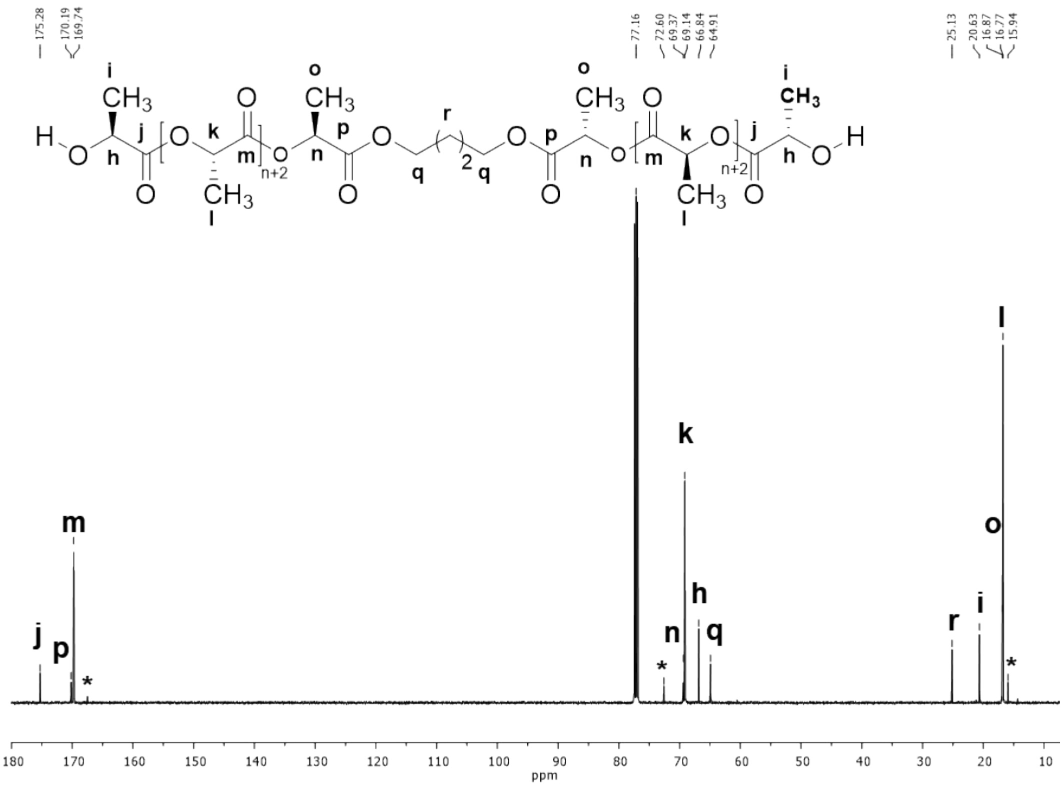

Figure S19. ^{13}C NMR spectrum of $HOPLLA_4OH$ (500 MHz, $CDCl_3$).

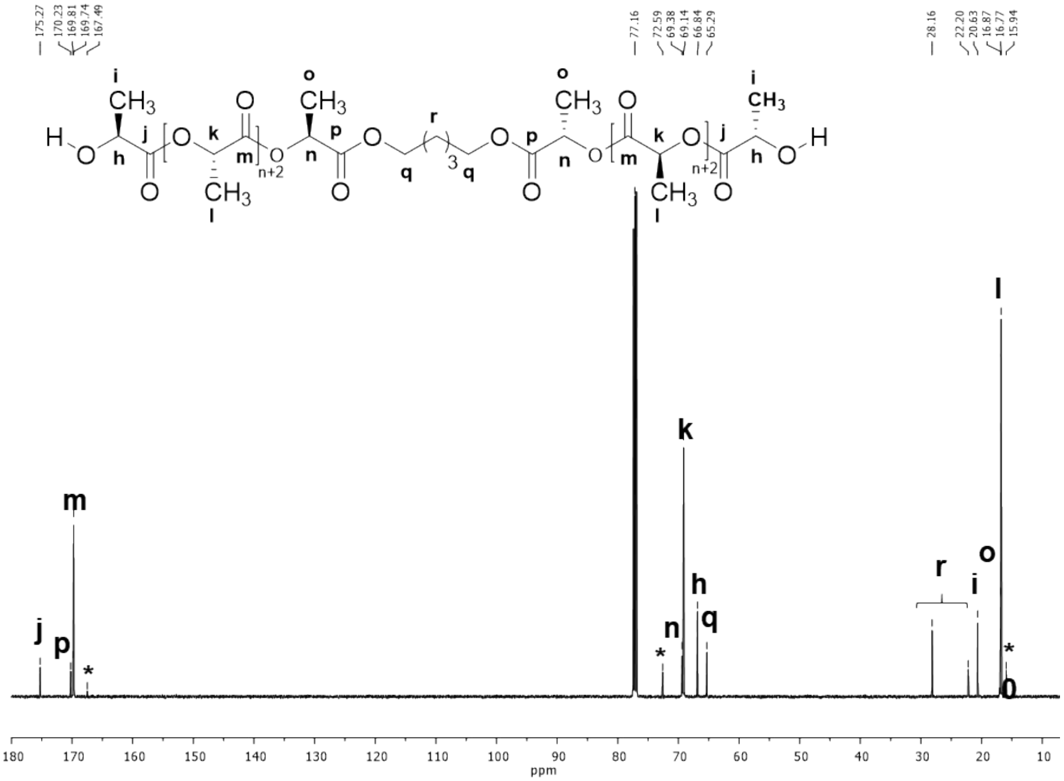

Figure S20. ^{13}C NMR spectrum of $HOPLLA_5OH$ (500 MHz, $CDCl_3$).

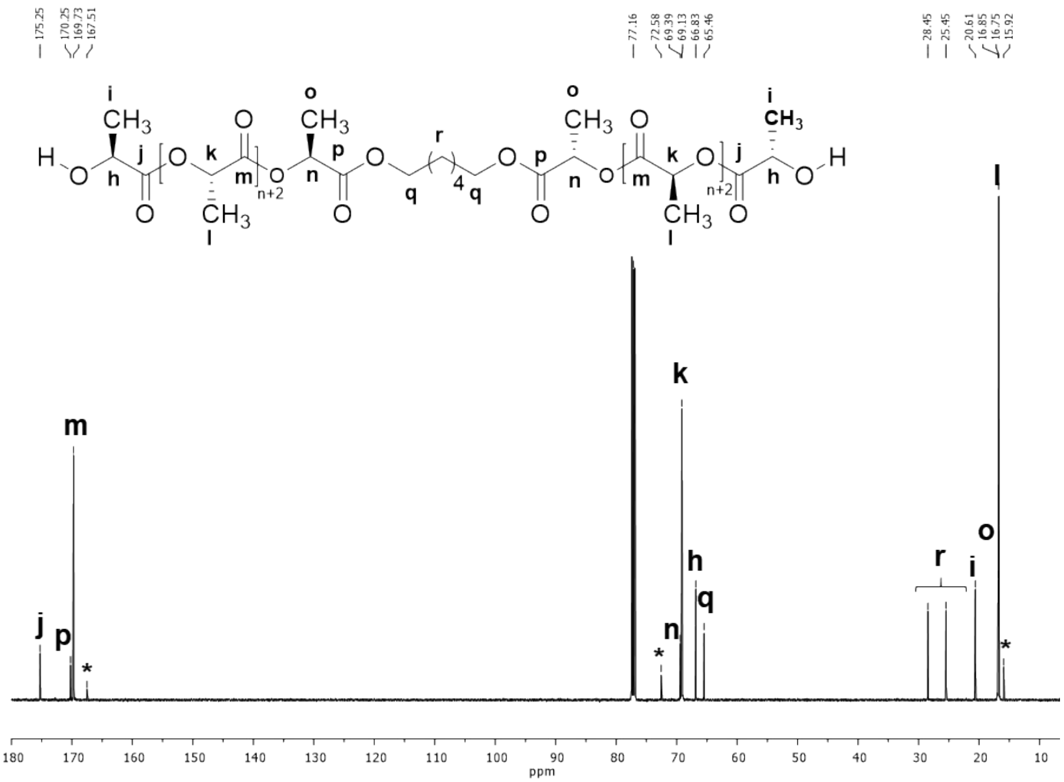

Figure S21. ^{13}C NMR spectrum of $HOPLLA_6OH$ (500 MHz, $CDCl_3$).

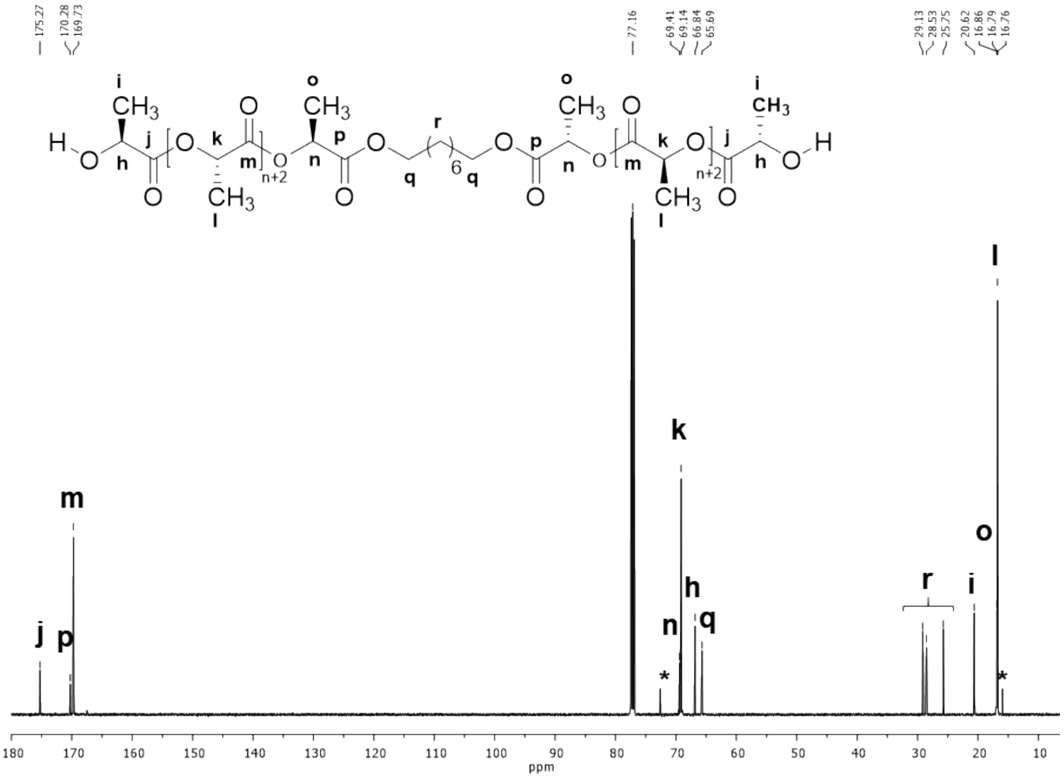

Figure S22. ^{13}C NMR spectrum of $HOPLLA_8OH$ (500 MHz, $CDCl_3$).

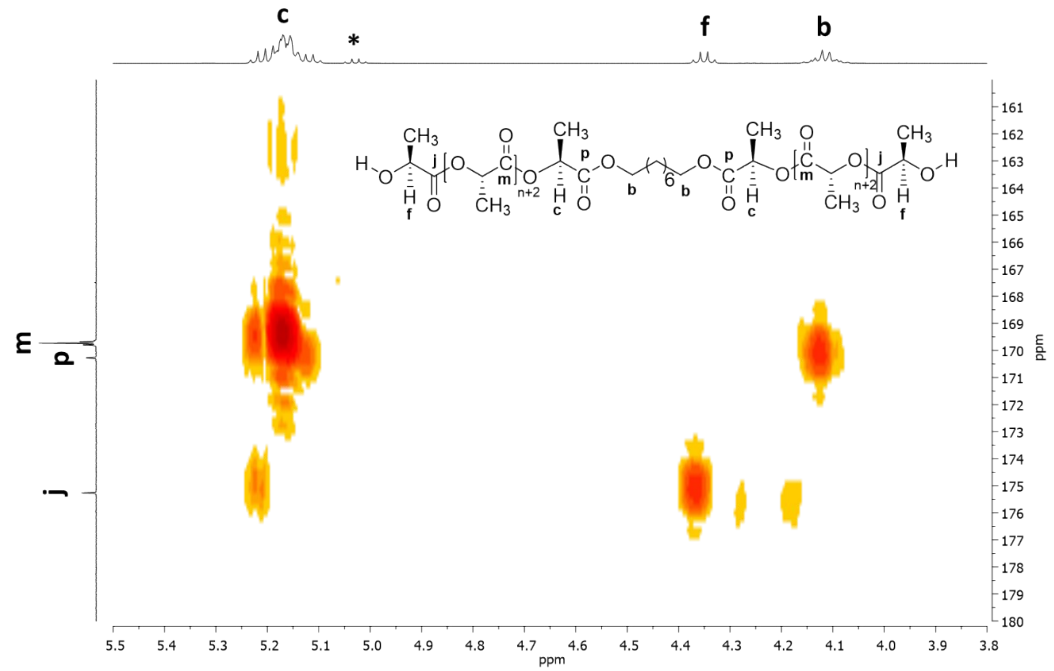

Figure S23. ^{1}H-^{13}C HMBC NMR spectrum of $HOPLLA_8OH$ (500 MHz, $CDCl_3$).

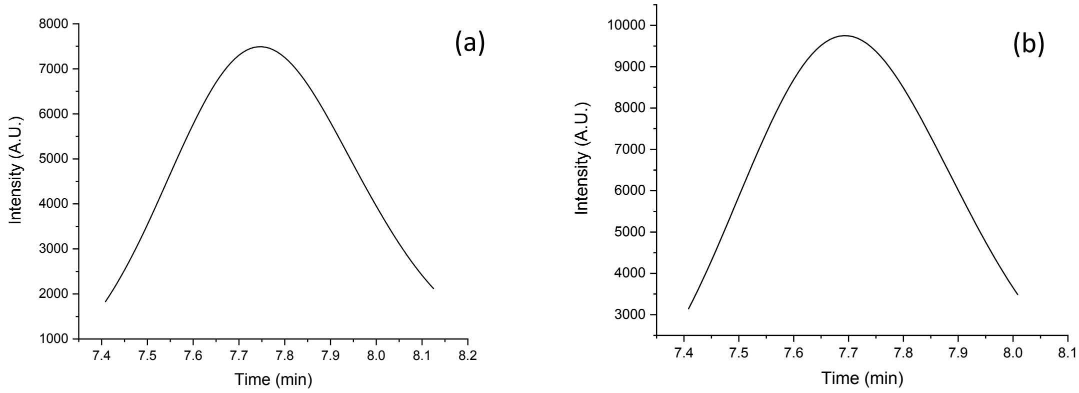

Figure S24. SEC chromatograms profile for a) $HOPLLA_2OH$, b) $HOPLLA_8OH$.

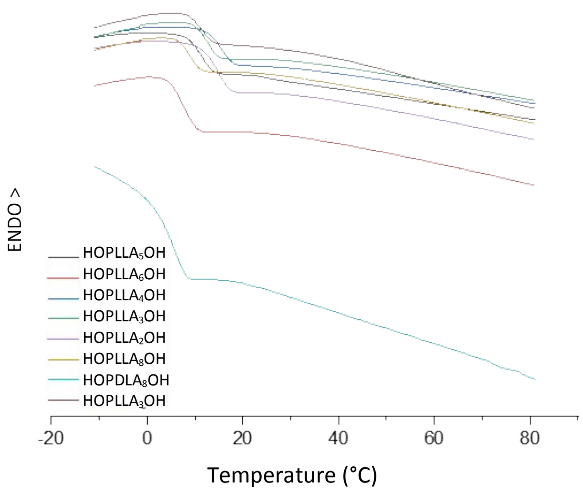

Figure S25. DSC thermograms of the different poly(L-lactide) macrodiols (HOPLLAOH) (all samples).

Computational Details

Geometry optimizations were performed in the gas phase using the hybrid PBE0 density functional which mixes 25% of HF exchange in its formulation.[1] Grimme's D3 method[2] for incorporating dispersion effects was explicitly included in the geometry optimizations. The electronic configurations of light atoms such as hydrogen and carbon were described with Pople's double-ζ 6-31G(d) basis set containing one polarization function. On the other hand, the pseudopotential LANL2DZ containing a polarization function was used for bismuth.[3] Thus, this level of theory to show the energy values can be written as D3-PBE0/[6-31G(d),LANL2DZ].

Table S1. Total energy barrier using the following diols as initiators of the ROP of L-Lactide catalyst with bismuth subsalicylate.

**Alkoxides**	**Total energy barrier (Kcal/mol) with:**			
	**ethylen glycol**	**1,3-propanediol**	**1,4-butanediol**	**1,5-pentanediol**
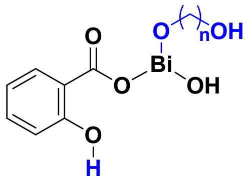 **(1)**	13.57	15.68	17.35	20.52
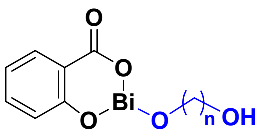 **(2)**	46.18	46.39	46.57	46.97

Note of Table S1: There is a trend, as the aliphatic chain of the diol used increases the total barrier in the two cases. However, the difference in energies obtained with alkoxide (2) is more significant. Therefore, the alkoxide (1) is the most reactive to carry out the polymerization of L-lactide.

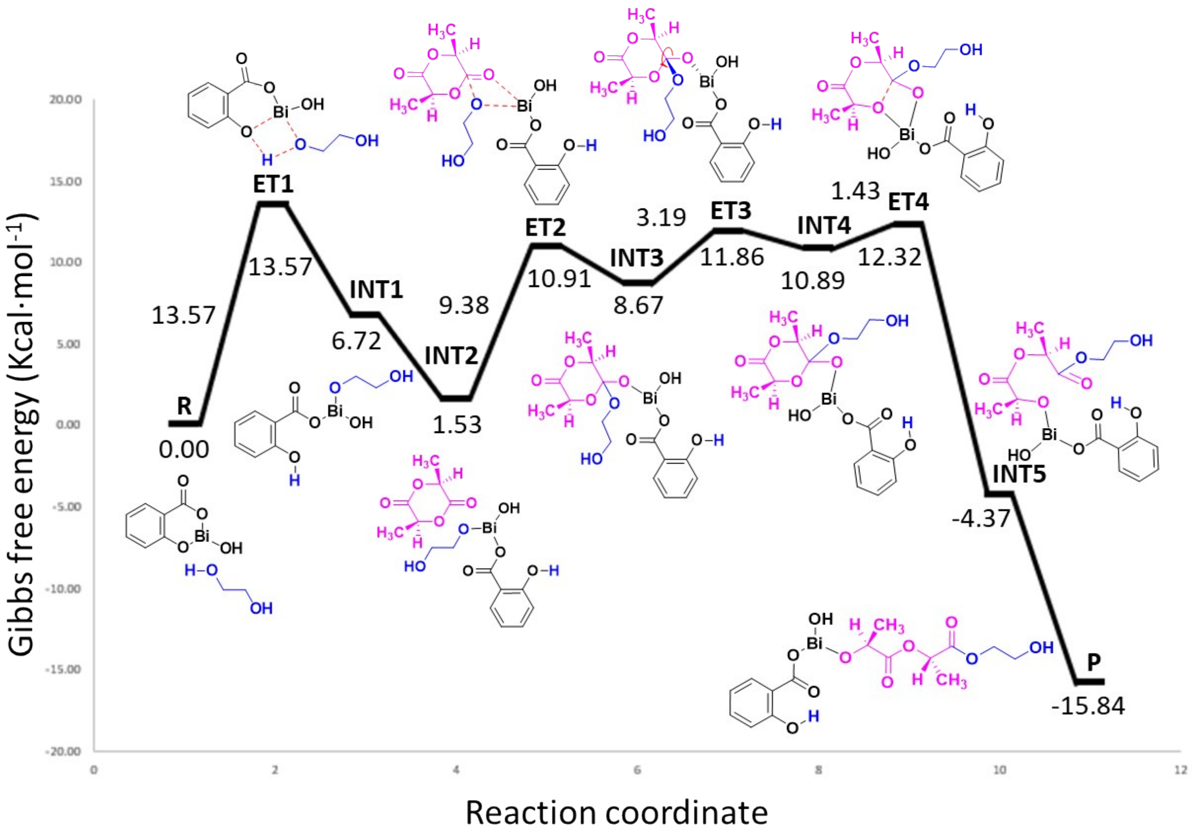

Scheme S1. Energy profile in gas phase at 140°C of the ROP of the L-lactide initiated with ethyleneglycol using bismuth subsalicylate as catalyst.

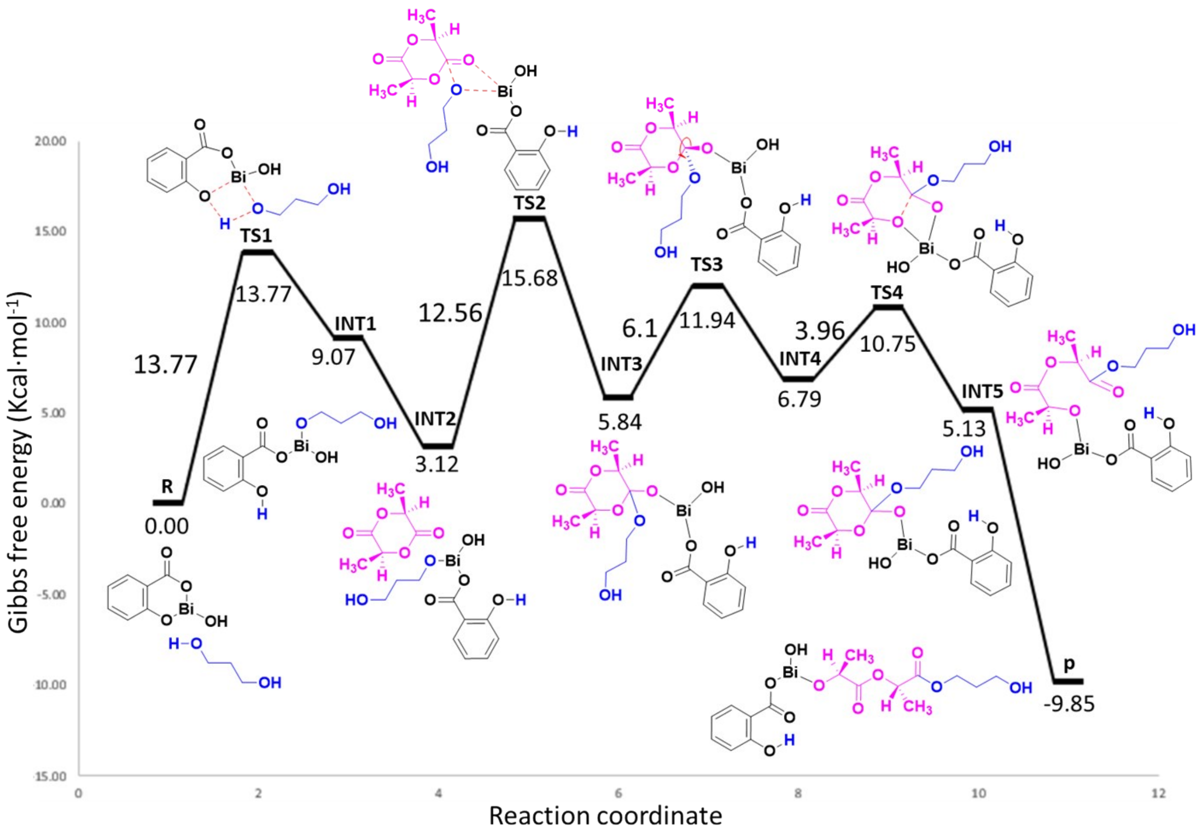

Scheme S2. Energy profile in gas phase at 140°C of the ROP of the L-lactide initiated with 1,3-propanediol using bismuth subsalicylate as catalyst.

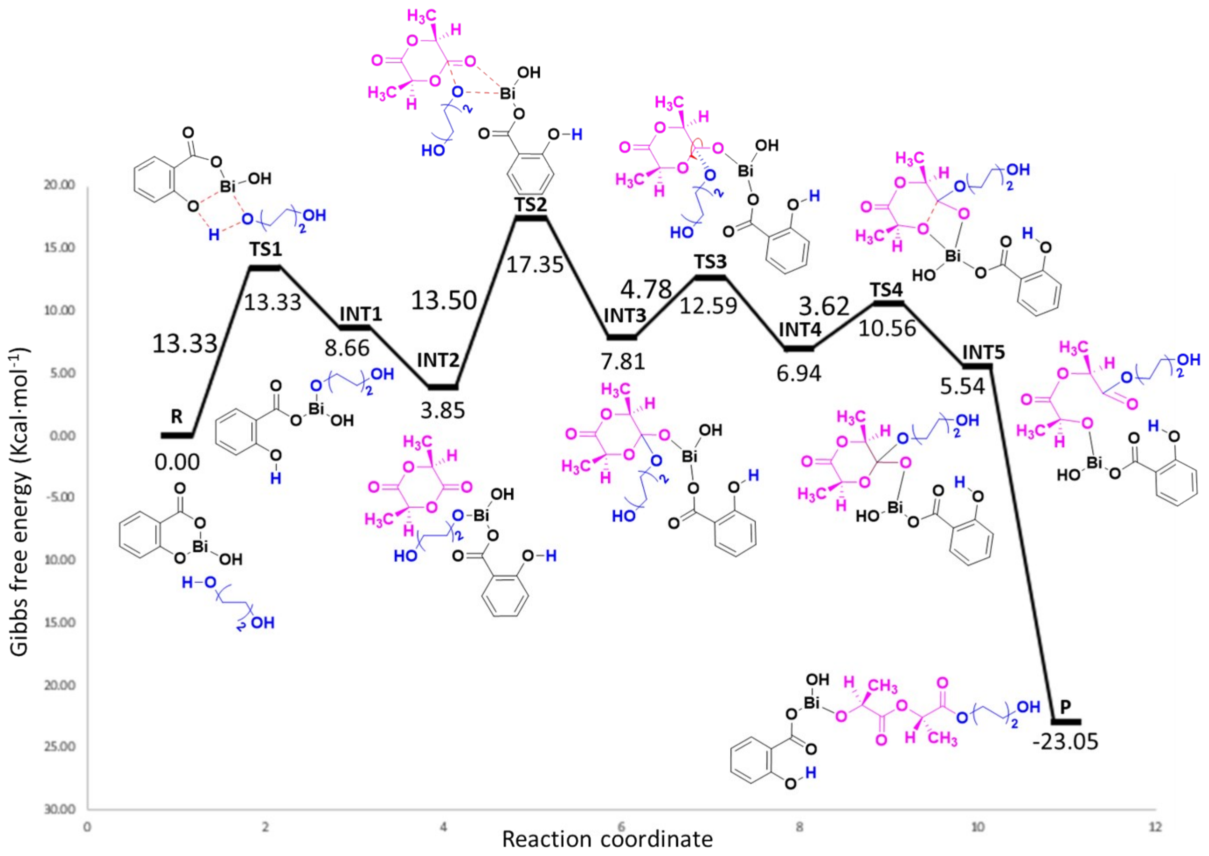

Scheme S3. Energy profile in gas phase at 140°C of the ROP of the L-lactide initiated with 1,4-butanediol using bismuth subsalicylate as catalyst.

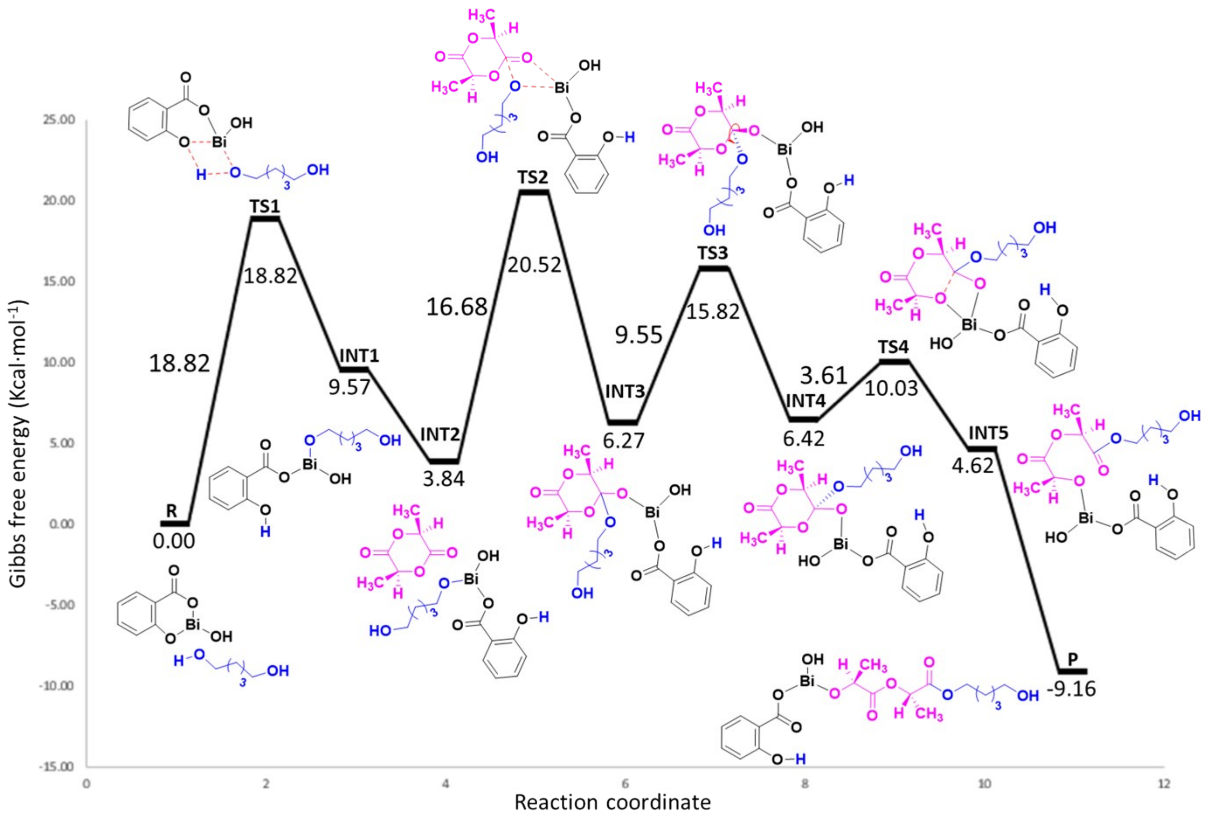

Scheme S4. Energy profile in gas phase at 140°C of the ROP of the L-lactide initiated with 1,5-pentanediol using bismuth subsalicylate as catalyst.

Table S2. Cartesian coordinates (xyz format) of the optimized geometries for all the species involved in the energy profile of the **Scheme S1** calculated at the D3-PBE0/[6-31G(d), LANL2DZ] level.

R

E(scf) = -805.501842111 a.u.

C	1.087312	1.204866	-0.876220
C	2.412341	-0.421903	0.599567
C	2.359273	0.634415	-0.318417
C	3.562087	1.191432	-0.764298
H	3.509277	2.035836	-1.444958
C	4.786172	0.670812	-0.364404
C	4.821075	-0.408311	0.516577
C	3.635913	-0.943360	1.009194
H	5.709324	1.104447	-0.737690
H	5.772634	-0.823373	0.837427
H	3.635519	-1.759602	1.725160
O	0.960047	2.400942	-1.030001
O	0.180040	0.326700	-1.297311
O	1.270789	-0.977369	1.138909
Bi	-1.050607	-0.959915	-0.226904
O	-2.763332	-0.191753	-0.905310
H	-2.746495	0.791217	-0.870434
C	-1.882080	2.499761	1.030649
C	-1.828838	1.229768	1.877836
H	-2.192322	3.339936	1.669119
H	-0.890714	2.724655	0.628608
H	-1.528659	1.508355	2.897453
H	-2.844259	0.805768	1.949245
O	-2.748089	2.418893	-0.096872
O	-0.902791	0.258482	1.449830
H	-3.658323	2.402055	0.230567
H	0.741543	-0.288384	1.611669

ET1

E(scf) = -805.490134714 a.u.

C	-1.311543	-1.538855	0.963805
C	-1.915335	0.231590	-0.827183
C	-2.251266	-0.629989	0.233102
C	-3.582821	-0.654486	0.673042
H	-3.819196	-1.341598	1.479052
C	-4.554280	0.162040	0.117490
C	-4.202616	1.028424	-0.918587
C	-2.897775	1.056840	-1.389310
H	-5.575343	0.127330	0.485341
H	-4.950599	1.674492	-1.373125
H	-2.613168	1.700818	-2.216899
O	-1.734859	-2.396290	1.706858
O	0.004784	-1.363975	0.842559
O	-0.672887	0.290809	-1.366155
Bi	1.361362	-0.539021	-0.476767
O	2.471495	-0.112228	1.151129
H	1.836498	0.149147	1.853911
C	0.271875	2.642532	1.377021
C	1.274563	2.640972	0.230651
H	0.285106	3.634528	1.854444
H	-0.739450	2.485835	0.984244
H	1.221504	3.602317	-0.298243
H	2.291495	2.522584	0.625889
O	0.491875	1.622251	2.337068
O	1.060273	1.622285	-0.727013
H	1.149789	1.954614	2.971796
H	-0.029914	1.291432	-1.110540

INT1

E(scf) = -805.514760750 a.u.

C	-2.140762	-1.572894	0.097835
C	-1.743611	0.941826	-0.428867
C	-2.527580	-0.121915	0.074993
C	-3.813150	0.166512	0.559174

INT2

E(scf) = -1339.28768843 a.u.

C	2.779911	2.329077	0.648331
C	2.819930	2.269953	-0.872205
C	3.143806	-0.081269	-0.662338
C	4.098411	0.351185	0.444052

H	-4.395301	-0.675811	0.919730
C	-4.329580	1.450477	0.564569
C	-3.552985	2.493115	0.055512
C	-2.282510	2.238955	-0.432936
H	-5.327584	1.639904	0.948342
H	-3.939901	3.509062	0.039329
H	-1.669746	3.038182	-0.841321
O	-2.970825	-2.432031	0.312072
O	-0.867350	-1.894378	-0.113390
O	-0.505578	0.813673	-0.947104
Bi	0.817061	-0.667611	-0.268630
O	0.672064	-0.054426	1.655460
H	-0.253132	-0.076614	1.944692
C	3.308882	1.285793	1.163923
C	2.982740	2.159430	-0.036169
H	4.213388	1.660086	1.664698
H	2.479856	1.282036	1.874129
H	2.713936	3.171656	0.287641
H	3.855099	2.244104	-0.697936
O	3.455078	-0.072096	0.774133
O	1.939851	1.572572	-0.806211
H	4.220058	-0.140826	0.184950
H	1.092719	2.045647	-0.735872

O	3.444882	1.329170	1.264519
O	2.537438	0.927140	-1.299379
O	2.242042	3.198205	1.282286
O	2.923934	-1.227801	-0.978785
C	1.830026	3.189182	-1.541015
H	1.993194	3.185698	-2.612757
H	0.807517	2.856395	-1.333165
H	1.963840	4.206019	-1.147985
H	3.843598	2.520540	-1.194323
C	4.525515	-0.788716	1.320668
H	5.054147	-1.546778	0.719361
H	5.192664	-0.423579	2.111806
H	3.658555	-1.277335	1.781281
H	4.972809	0.820094	-0.029588
C	-3.131844	-0.936876	-0.080435
C	-2.330831	1.525177	-0.412996
C	-3.301915	0.563640	-0.095407
C	-4.580851	1.027648	0.269345
H	-5.318538	0.269545	0.530650
C	-4.886064	2.380189	0.306946
C	-3.912369	3.320033	-0.019226
C	-2.634493	2.887688	-0.369801
H	-5.892036	2.703542	0.579085
H	-4.141264	4.389946	-0.003914
H	-1.863603	3.603744	-0.631877
O	-3.974140	-1.641457	0.463714
O	-2.118701	-1.456735	-0.752796
O	-1.048968	1.172309	-0.782654
Bi	-0.009137	-1.296379	-0.691870
O	0.287334	-2.918712	0.464379
H	-0.335201	-2.837293	1.209775
C	0.625018	-0.208777	2.179941
C	-0.772473	-0.332521	2.785695
H	1.175278	0.594509	2.690780
H	1.174383	-1.152162	2.332253
H	-0.704944	-0.563142	3.864092
H	-1.322412	0.612542	2.661218
O	0.555258	0.115787	0.808418
O	-1.387779	-1.384352	2.059442
H	-0.457068	1.200731	0.020944
H	-2.363593	-1.445705	2.235114

ET2					INT3		
E(scf) = -1339.27719490 a.u.				E(scf) = -1339.28647826 a.u.			
C	2.122911	3.201694	0.491516	C	1.819987	-0.221655	-0.406675
C	0.889124	3.101561	-0.403590	C	3.186637	-0.227730	-1.136282
C	1.638918	0.852138	-0.960784	C	3.444177	2.114596	-0.932053
C	3.057094	1.321125	-0.683016	C	1.934209	2.086819	-1.131575
O	3.115262	2.312993	0.356754	O	1.381965	1.132673	-0.217434
O	0.791958	1.914404	-1.205127	O	3.999731	0.896693	-0.771370
O	2.206249	4.072562	1.317418	O	0.959005	-0.906766	-1.204425
O	1.507763	-0.092372	-1.806019	O	4.109213	3.113627	-0.879544
C	-0.376575	3.278821	0.418974	C	3.985634	-1.480753	-0.872090
H	-1.247024	3.347968	-0.244168	H	3.382084	-2.356970	-1.123071
H	-0.518522	2.429342	1.087206	H	4.890053	-1.481750	-1.486487
H	-0.301937	4.192018	1.008023	H	4.276069	-1.544182	0.179549
H	0.981940	3.926560	-1.117315	H	2.957367	-0.155385	-2.206311
C	4.041959	0.200497	-0.371648	C	1.307007	3.433349	-0.855528
H	4.642827	-0.051365	-1.290829	H	1.767612	4.190058	-1.494393
H	4.745939	0.556379	0.437489	H	0.232421	3.402931	-1.057935
H	3.513215	-0.725705	-0.031485	H	1.470925	3.720963	0.186463
H	3.344600	1.818442	-1.617966	H	1.713883	1.772143	-2.164206
C	-2.202787	-0.806188	1.165984	C	-1.911634	0.404518	1.648308
C	-2.513730	0.672766	-0.892544	C	-2.385036	1.336893	-0.695577
C	-2.952388	0.197817	0.350673	C	-2.763024	1.111171	0.635117
C	-4.128345	0.731616	0.889868	C	-4.004060	1.595210	1.065105
H	-4.436950	0.375635	1.867829	H	-4.264124	1.446009	2.108531
C	-4.867900	1.687427	0.208256	C	-4.869321	2.235658	0.190024
C	-4.421221	2.144533	-1.030642	C	-4.489463	2.423225	-1.138827
C	-3.247095	1.640867	-1.577941	C	-3.246623	1.983851	-1.578344
H	-5.783493	2.079409	0.640602	H	-5.833893	2.590931	0.540000
H	-4.987985	2.894005	-1.576179	H	-5.156814	2.926085	-1.833292
H	-2.891655	1.982622	-2.546703	H	-2.916753	2.139924	-2.600893
O	-2.313784	-0.796801	2.389918	O	-2.039699	0.636971	2.831746
O	-1.493245	-1.703604	0.539251	O	-1.015881	-0.475667	1.217329
O	-1.34973	0.200421	-1.447655	O	-1.184959	0.896833	-1.208691
Bi	0.249196	-1.712061	-0.678043	Bi	-0.859089	-1.653458	-0.513356
O	1.374759	-2.735920	0.643371	O	-0.023787	-3.117574	0.566617
H	1.068714	-2.550530	1.558377	H	0.725117	-2.748976	1.064120
C	1.645217	0.028748	1.723036	C	1.522917	0.455816	2.884330
C	0.723871	-0.253697	2.892603	C	2.511104	-0.179414	1.924349

H	2.162562	0.969637	1.911932
H	2.390641	-0.770159	1.660565
H	1.308134	-0.062507	3.804511
H	-0.123006	0.444116	2.887900
O	0.946471	0.154143	0.469861
O	0.272769	-1.591676	2.914908
H	-0.763692	0.944854	-1.691746
H	-0.709671	-1.571205	2.823643

ET3

E(scf) = -1339.27719490 a.u.

C	-1.385728	0.522987	0.556044
C	-1.596330	1.682447	1.561992
C	-2.355677	3.137478	-0.145228
C	-1.337924	2.363110	-0.969528
O	-1.610971	0.973354	-0.755088
O	-2.572840	2.613420	1.086723
O	-0.100153	0.069929	0.720245
O	-2.972892	4.096674	-0.519592
C	-2.024760	1.218817	2.933707
H	-1.296150	0.504226	3.326662
H	-2.084757	2.074345	3.611816
H	-3.002195	0.731650	2.895200
H	-0.631843	2.200879	1.633791
C	-1.439715	2.645850	-2.447446
H	-1.332504	3.718903	-2.621749
H	-0.637926	2.117489	-2.969293
H	-2.414299	2.331959	-2.832510
H	-0.317332	2.590272	-0.628254
C	2.676569	1.132516	1.371080
C	2.566935	-1.123900	2.556403
C	3.053859	0.187135	2.471206
C	3.963608	0.628255	3.435887
H	4.307870	1.656805	3.380833
C	4.431626	-0.230973	4.422745
C	3.972064	-1.545412	4.467113
C	3.028670	-1.986657	3.544337
H	5.154029	0.122069	5.152784
H	4.332719	-2.225796	5.233611
H	2.627046	-2.994716	3.581730
O	2.456325	2.305658	1.587070

H	0.759206	-0.280713	3.166255
H	1.006080	1.287480	2.386395
H	3.074450	-0.956097	2.452032
H	3.224697	0.562281	1.557030
O	2.289178	0.890716	3.988171
O	1.835206	-0.828358	0.843596
H	1.677156	1.250893	4.641615
H	-0.415965	1.128841	-0.636470

INT4

E(scf) = -1339.28282663 a.u.

C	-1.405269	0.525532	0.556053
C	-1.615871	1.684992	1.562001
C	-2.375218	3.140023	-0.145219
C	-1.357465	2.365655	-0.969519
O	-1.630512	0.975899	-0.755079
O	-2.592381	2.615965	1.086732
O	-0.119694	0.072474	0.720254
O	-2.992433	4.099219	-0.519583
C	-2.044301	1.221362	2.933716
H	-1.315691	0.506771	3.326671
H	-2.104298	2.076890	3.611825
H	-3.021736	0.734195	2.895209
H	-0.651384	2.203424	1.633800
C	-1.459256	2.648395	-2.447437
H	-1.352045	3.721448	-2.621740
H	-0.657467	2.120034	-2.969284
H	-2.433840	2.334504	-2.832501
H	-0.336873	2.592817	-0.628245
C	2.203459	0.673319	-1.151860
C	3.314783	0.047541	1.057616
C	3.315464	0.757245	-0.150476
C	4.420376	1.555551	-0.458234
H	4.395506	2.128587	-1.380445
C	5.528055	1.596677	0.379954
C	5.530858	0.849745	1.555965
C	4.418766	0.088390	1.902077
H	6.386989	2.206581	0.115925
H	6.392410	0.873626	2.217532
H	4.381921	-0.471879	2.831392
O	1.805526	1.653545	-1.745347

O	2.721442	0.607725	0.147667
O	1.600686	-1.596392	1.686289
Bi	1.238899	-0.469429	-0.817951
O	0.847784	0.963779	-2.163220
H	0.559184	1.783220	-1.727018
C	-4.284895	-1.687408	0.915235
C	-3.557699	-0.500037	0.320506
H	-4.215002	-1.645682	2.014289
H	-3.786092	-2.611783	0.583091
H	-3.600874	-0.544113	-0.771281
H	-4.025375	0.433194	0.654183
O	-5.615347	-1.607993	0.463023
O	-2.207700	-0.585705	0.768502
H	-6.093014	-2.377005	0.796527
H	0.779176	-1.079012	1.821082

ET4

E(scf) = -1339.28676053 a.u.

C	-1.418318	1.166956	0.601006
C	-1.703184	1.865134	-0.719332
C	-3.693390	0.695562	-1.422386
C	-2.942490	-0.604297	-1.148677
O	-2.215000	-0.528978	0.067756
O	-3.081740	1.858309	-1.067687
O	-0.206907	0.747298	0.772791
O	-4.785877	0.747200	-1.916565
C	-1.231766	3.323981	-0.674504
H	-0.572134	3.510366	0.222500
H	-0.648520	3.572792	-1.608829
H	-2.140854	3.988478	-0.622867
H	-1.127182	1.294520	-1.456081
C	-3.906920	-1.777493	-1.107738
H	-4.507240	-1.813043	-2.019629
H	-3.346945	-2.713863	-1.016683
H	-4.579193	-1.686916	-0.248638
H	-2.237491	-0.730217	-1.987442
C	2.744910	-0.034945	-0.687291
C	4.712732	0.109252	0.921013
C	4.115815	0.387977	-0.328995
C	4.848192	1.102420	-1.289819

O	1.783371	-0.563317	-1.413424
O	2.218259	-0.692048	1.463360
Bi	0.379177	-1.666860	-0.364117
O	-0.998645	-1.599770	-1.818047
H	-1.247757	-0.682371	-2.021437
C	-4.304436	-1.684863	0.915244
C	-3.577240	-0.497492	0.320515
H	-4.234543	-1.643137	2.014298
H	-3.805633	-2.609238	0.583100
H	-3.620415	-0.541568	-0.771272
H	-4.044916	0.435739	0.654192
O	-5.634888	-1.605448	0.463032
O	-2.227241	-0.583160	0.768511
H	-6.112555	-2.374460	0.796536
H	1.463484	-0.081935	1.600500

INT5

E(scf) = -1339.31061838 a.u.

C	-3.099904	0.830145	-0.349082
C	-3.941947	-0.365192	-0.798309
C	-2.820481	-2.375590	0.012496
C	-1.524987	-1.907997	-0.659145
O	-1.129781	-0.769682	0.062912
O	-3.873897	-1.505177	0.046917
O	-2.420382	1.409999	-1.199237
O	-2.949003	-3.432982	0.564738
C	-5.407409	0.025782	-0.920850
H	-5.512216	0.876086	-1.601698
H	-5.973035	-0.820035	-1.320904
H	-5.833028	0.299303	0.048373
H	-3.558448	-0.611181	-1.792548
C	-0.485916	-3.013599	-0.627259
H	-0.846086	-3.908384	-1.143331
H	0.433770	-2.671465	-1.112139
H	-0.262200	-3.278799	0.408824
H	-1.755247	-1.670745	-1.717295
C	2.693215	0.412757	-0.181760
C	5.059737	1.040602	0.126867
C	4.075255	0.038767	0.118794
C	4.437470	-1.298421	0.403357

H	4.362871	1.299894	-2.240486	H	2.676352	-1.940756	0.227553
C	6.135723	1.537656	-1.036717	C	5.778817	-1.592677	0.684242
C	6.715858	1.257517	0.205845	C	6.729818	-0.587616	0.682195
C	6.017340	0.554916	1.172249	C	6.379167	0.739925	0.403395
H	6.688657	2.088181	-1.791535	H	6.039843	-2.623255	0.902689
H	7.727502	1.593003	0.419969	H	7.764450	-0.837374	0.903066
H	6.454304	0.329997	2.140137	H	7.133311	1.520490	0.405883
O	2.244405	0.256373	-1.773775	O	1.787532	-0.524713	-0.232495
O	2.109269	-0.752842	0.229417	O	2.338197	1.596459	-0.396324
O	4.094896	-0.567660	1.898797	O	3.565346	-2.311309	0.424817
Bi	-0.020310	-1.288441	0.128295	Bi	0.014885	0.746971	-0.796206
O	-0.084071	-1.042519	-1.853013	O	-0.127464	1.679809	0.992162
H	0.753810	-0.573235	-2.096932	H	0.723116	2.112131	1.166546
C	-4.431161	0.967465	1.742980	C	-2.566393	0.231894	2.897748
C	-3.332376	2.029088	1.872854	C	-3.721491	0.697932	2.025185
H	-5.292594	1.317349	2.323068	H	-2.998960	-0.186944	3.815543
H	-4.769770	0.889161	0.702732	H	-2.027248	-0.573692	2.379788
H	-3.301281	2.418437	2.891860	H	-4.276530	1.493637	2.527200
H	-3.483639	2.855437	1.172927	H	-4.387334	-0.123692	1.757984
O	-4.045798	-0.273250	2.260425	O	-1.723870	1.284635	3.254260
O	-2.018618	1.459309	1.728221	O	-3.174006	1.340979	0.850539
H	-3.371225	-0.611032	1.642241	H	-1.173343	1.485215	2.469459
H	3.206436	-0.801761	1.551351	H	4.746332	2.056480	-0.093411

p

E(scf) = -1339.32358607 a.u.

C	3.926179	0.565060	0.504665
C	2.828248	1.251215	1.309466
C	1.266653	-0.490750	1.211037
C	0.530482	-1.574400	1.978966
O	-0.267605	-2.340894	1.149804
O	2.059456	0.253113	1.977677
O	4.206021	-0.604297	0.569286
O	1.121526	-0.294422	0.015593
C	3.395678	2.170655	2.375405
H	3.992985	2.957053	1.907249
H	2.580723	2.633011	2.938946
H	4.027482	1.609082	3.070152
H	2.185487	1.800706	0.614404
C	1.527102	-2.470300	2.714061

H	2.104059	-1.903019	3.449533
H	0.968662	-3.260592	3.221972
H	2.217360	-2.931524	2.001298
H	-0.083579	-1.041566	2.729187
C	-2.671698	0.524085	-0.559214
C	-4.401875	2.157095	-1.220451
C	-3.410145	1.767525	-0.307161
C	-3.146460	2.577026	0.820976
H	-1.814186	1.424169	1.472105
C	-3.883534	3.755156	1.000240
C	-4.855850	4.117316	0.084046
C	-5.124591	3.320867	-1.035796
H	-3.669126	4.363918	1.872932
H	-5.417484	5.034604	0.241883
H	-5.889736	3.612775	-1.748251
O	-1.740331	0.180172	0.293068
O	-2.884908	-0.206225	-1.546565
O	-2.220008	2.279698	1.740271
Bi	-1.030581	-1.713183	-0.652259
O	-2.669165	-2.805969	-0.187644
H	-3.419042	-2.463509	-0.696140
C	6.140591	2.104736	-1.892625
C	5.623407	0.949329	-1.063952
H	5.324240	2.494113	-2.520586
H	6.463166	2.916995	-1.222297
H	6.409179	0.548238	-0.416734
H	5.259602	0.138666	-1.701664
O	7.204275	1.590027	-2.657139
O	4.548795	1.461444	-0.267611
H	7.546908	2.300495	-3.212889
H	-4.578059	1.511678	-2.075524

Table S3. Cartesian coordinates (xyz format) of the optimized geometries for all the species involved in the energy profile of the **Scheme S2** calculated at the D3-PBE0/[6-31G(d), LANL2DZ] level.

R

E(scf) = -844.776731998 a.u.

C	-2.538121	-1.298298	0.680305
C	-2.077701	1.095903	-0.231338
C	-2.893342	0.128890	0.396281
C	-4.181345	0.512351	0.804786
H	-4.782557	-0.252474	1.285804
C	-4.671023	1.791010	0.609240
C	-3.857664	2.736856	-0.017512
C	-2.582505	2.390966	-0.429979
H	-5.672671	2.051659	0.937475
H	-4.218791	3.748345	-0.185907
H	-1.936780	3.113047	-0.921575
O	-3.313565	-2.033815	1.253737
O	-1.351965	-1.763799	0.281119
O	-0.820936	0.896013	-0.678451
Bi	0.329292	-0.835999	0.516418
O	1.079826	-0.617812	1.350361
H	0.419134	-0.915289	1.995069
C	4.937063	1.242313	1.037274
C	3.867899	0.791924	0.059438
H	4.536214	1.209001	2.063678
H	5.218307	2.288857	0.828054
C	2.636229	1.667827	0.115577
H	4.284182	0.801816	-0.954673
H	3.592228	-0.240545	0.303281
H	2.168610	1.614332	1.104757
H	2.889760	2.711864	-0.110949
O	6.037977	0.374902	0.884012
O	1.708767	1.208177	-0.879139
H	6.707770	0.624700	1.532791
H	0.905552	1.764831	-0.852627

ET1

E(scf) = -844.752929915 a.u.

C	-1.287728	0.462857	1.573434
C	-2.248863	0.758410	-0.807228
C	-2.320127	0.919660	0.582579
C	-3.451520	1.548811	1.116548
H	-3.480368	1.702222	2.190692
C	-4.504494	1.951315	0.306876
C	-4.431124	1.748851	-1.069931
C	-3.299059	1.164679	-1.625398
H	-5.378954	2.421514	0.746883
H	-5.247943	2.059928	-1.715325
H	-3.200243	1.022911	-2.697193
O	-1.119424	1.071893	2.607418
O	-0.651480	-0.685131	1.346603
O	-1.152960	0.194615	-1.428495
Bi	0.390049	-1.535814	-0.238698
O	1.754217	-2.097749	1.141867
H	1.597858	-1.607970	1.965358
C	3.864941	2.761694	0.308363
C	2.863930	2.037548	-0.571924
H	4.642875	2.055773	0.645996
H	3.358460	3.143428	1.210817
C	2.197886	0.876180	0.142257
H	2.101172	2.752659	-0.902869
H	3.373881	1.669803	-1.469752
H	2.948785	0.141863	0.466372
H	1.678222	1.230922	1.046652
O	4.421310	3.812862	-0.451791
O	1.274911	0.263905	-0.745731
H	5.046267	4.287148	0.110777
H	-0.329676	0.729181	-1.278901

INT1

E(scf) = -844.761349720 a.u.

C	-1.287728	0.462857	1.573434

INT2

E(scf) = -1378.54792441 a.u.

C	3.072326	2.350215	0.686509

C	-2.248863	0.758410	-0.807228
C	-2.320127	0.919660	0.582579
C	-3.451520	1.548811	1.116548
H	-3.480368	1.702222	2.190692
C	-4.504494	1.951315	0.306876
C	-4.431124	1.748851	-1.069931
C	-3.299059	1.164679	-1.625398
H	-5.378954	2.421514	0.746883
H	-5.247943	2.059928	-1.715325
H	-3.200243	1.022911	-2.697193
O	-1.119424	1.071893	2.607418
O	-0.651480	-0.685131	1.346603
O	-1.152960	0.194615	-1.428495
Bi	0.390049	-1.535814	-0.238698
O	1.754217	-2.097749	1.141867
H	1.597858	-1.607970	1.965358
C	3.864941	2.761694	0.308363
C	2.863930	2.037548	-0.571924
H	4.642875	2.055773	0.645996
H	3.358460	3.143428	1.210817
C	2.197886	0.876180	0.142257
H	2.101172	2.752659	-0.902869
H	3.373881	1.669803	-1.469752
H	2.948785	0.141863	0.466372
H	1.678222	1.230922	1.046652
O	4.421310	3.812862	-0.451791
O	1.274911	0.263905	-0.745731
H	5.046267	4.287148	0.110777
H	-0.329676	0.729181	-1.278901

C	2.512836	2.631281	-0.702572
C	2.707474	0.301877	-1.172675
C	3.995386	0.422969	-0.381915
O	3.756813	1.194222	0.802320
O	1.998331	1.420188	-1.296465
O	2.952078	3.096310	1.616595
O	2.313026	-0.726924	-1.678715
C	1.396129	3.645482	-0.690540
H	1.102798	3.891062	-1.714514
H	0.528454	3.242976	-0.161478
H	1.736045	4.550486	-0.182783
H	3.345923	2.980203	-1.331919
C	4.548617	-0.917100	0.036435
H	4.761719	-1.523485	-0.846780
H	5.468072	-0.772144	0.608690
H	3.821108	-1.449010	0.654346
H	4.725152	0.959378	-1.009531
C	-2.911111	-1.049362	0.529035
C	-2.615412	1.498996	0.276782
C	-3.374886	0.376034	0.635497
C	-4.677387	0.576370	1.106527
H	-5.239039	-0.298480	1.419576
C	-5.241232	1.844003	1.156573
C	-4.488498	2.945403	0.753797
C	-3.176139	2.773371	0.330476
H	-6.260786	1.973383	1.507698
H	-4.915322	3.944170	0.788895
H	-2.557268	3.620450	0.049923
O	-3.245565	-1.869635	1.359675
O	-2.236208	-1.387891	-0.561746
O	-1.306312	1.406853	-0.129403
Bi	-0.382612	-0.862316	-1.405875
O	0.017811	-2.840882	-1.350292
H	-0.699246	-3.253466	-0.840675
C	1.274376	-2.032667	3.755873
C	1.096359	-0.943731	2.714452
H	1.901543	-2.842707	3.344044
H	0.294066	-2.474096	3.999826
C	0.490936	-1.478984	1.429148
H	0.451035	-0.159699	3.128486
H	2.06899	-0.481437	2.506579

H	1.133422	-2.25724	0.99292
H	-0.48426	-1.944234	1.638548
O	1.874229	-1.449528	4.892673
O	0.344746	-0.409806	0.503993
H	1.92927	-2.126791	5.578395
H	-0.765123	0.864655	0.507916

ET2

E(scf) = 1378.52715869 a.u.

C	3.116032	2.369008	-0.204409
C	2.406709	2.159751	-1.535553
C	1.980352	-0.122446	-0.870352
C	3.373401	0.014199	-0.285363
O	3.510184	1.247323	0.430916
O	1.550854	1.007229	-1.533718
O	3.346242	3.452798	0.260979
O	1.643114	-1.217945	-1.417301
C	1.571593	3.360561	-1.930009
H	1.125580	3.193804	-2.910488
H	0.780389	3.534246	-1.192728
H	2.206061	4.250590	-1.969981
H	3.191395	1.992137	-2.288712
C	3.762018	-1.128253	0.623887
H	4.744549	-0.927702	1.058188
H	3.029818	-1.272778	1.418417
H	3.807047	-2.050862	0.039588
H	4.054606	0.037815	-1.150637
C	-2.496533	-0.293456	1.134220
C	-2.218248	0.598886	-1.296089
C	-2.895749	0.516746	-0.074472
C	-4.037205	1.324707	0.085832
H	-4.539589	1.274940	1.047285
C	-4.489441	2.165174	-0.916394
C	-3.788125	2.235995	-2.118547
C	-2.656612	1.455795	-2.305335
H	-5.376221	2.772120	-0.757046
H	-4.128445	2.898080	-2.914533
H	-2.107838	1.483448	-3.244597
O	-2.915638	0.036212	2.224289
O	-1.746672	-1.367105	0.992862

INT3

E(scf) = -1378.54831271 a.u.

C	3.432044	1.076717	-2.014769
C	1.912174	1.105587	-2.115821
C	1.702558	-0.633823	-0.448121
C	3.004621	-1.086519	-1.157469
O	3.919014	0.000848	-1.362423
O	1.383320	0.707950	-0.846879
O	4.164669	1.935229	-2.427100
O	0.738211	-1.505079	-0.842508
C	1.396935	2.488350	-2.440213
H	0.309648	2.471088	-2.557735
H	1.663074	3.189023	-1.644130
H	1.849832	2.840443	-3.369760
H	1.586456	0.390982	-2.888427
C	3.734484	-2.175312	-0.408931
H	4.580375	-2.532481	-1.002567
H	4.109393	-1.810840	0.550703
H	3.050486	-3.007728	-0.223937
H	2.693643	-1.463343	-2.139024
C	-1.923569	1.022322	1.372591
C	-2.411677	1.045593	-1.154387
C	-2.751703	1.366331	0.167908
C	-3.936570	2.077380	0.393270
H	-4.165644	2.347597	1.419448
C	-4.782907	2.420062	-0.650871
C	-4.442399	2.066738	-1.956376
C	-3.256408	1.388229	-2.207845
H	-5.702607	2.961877	-0.451450
H	-5.095591	2.330464	-2.783677
H	-2.957470	1.119263	-3.216391
O	-2.062897	1.629728	2.410748
O	-1.028121	0.043252	1.263014

O	-1.097201	-0.168392	-1.541410
Bi	-0.152583	-2.110209	-0.183200
O	0.630583	-2.976658	1.473375
H	0.280893	-2.570046	2.281612
C	-0.352330	2.479936	2.860840
C	0.176198	1.925956	1.546935
H	-1.200689	1.866030	3.191268
H	0.436643	2.421413	3.634244
C	0.754666	0.528493	1.720213
H	-0.650163	1.894025	0.823887
H	0.929053	2.620763	1.163298
H	1.724960	0.566919	2.226463
H	0.080333	-0.073245	2.351565
O	-0.736471	3.819508	2.627917
O	0.882471	-0.188840	0.505696
H	-1.160062	4.146431	3.434922
H	-0.344045	0.389835	-1.837696

ET3

E(scf) = -1378.54772519 a.u.

C	-1.810699	3.462210	-0.088099
C	-0.853450	2.623932	-0.922406
C	-1.131740	0.734994	0.514356
C	-1.245509	1.864870	1.568043
O	-2.108947	2.913422	1.115510
O	-1.279856	1.264547	-0.779574
O	-2.325778	4.489575	-0.436205
O	0.096126	0.145124	0.678334
C	-0.880786	2.980346	-2.387629
H	-0.127328	2.388440	-2.913395
H	-1.872203	2.793282	-2.810355
H	-0.651161	4.041274	-2.510589
H	0.175066	2.725560	-0.546092
C	-1.753785	1.389096	2.907763
H	-1.745909	2.216318	3.622610
H	-2.772575	1.002531	2.826220
H	-1.110383	0.588677	3.283473
H	-0.234912	2.276499	1.682672
C	2.792837	-0.510877	1.831802
C	1.967243	-2.727452	0.878639

O	-1.275163	0.337364	-1.479755
Bi	-1.067344	-1.74077	0.167438
O	-0.245284	-2.715128	1.713658
H	0.535136	-2.220729	2.013849
C	2.747205	2.389678	2.976304
C	1.852453	1.516801	2.114507
H	3.664398	2.651051	2.418697
H	3.064281	1.827827	3.872035
C	2.611316	0.307103	1.598579
H	0.976902	1.197448	2.689112
H	1.483629	2.111057	1.273499
H	3.426573	0.607382	0.931924
H	3.058833	-0.246403	2.433261
O	2.017142	3.543913	3.324806
O	1.765792	-0.644721	0.939462
H	2.572833	4.084735	3.899826
H	-0.459457	0.716937	-1.076960

INT4

E(scf) = -1378.54909419 a.u.

C	-1.810699	3.462210	-0.088099
C	-0.853450	2.623932	-0.922406
C	-1.131740	0.734994	0.514356
C	-1.245509	1.864870	1.568043
O	-2.108947	2.913422	1.115510
O	-1.279856	1.264547	-0.779574
O	-2.325778	4.489575	-0.436205
O	0.096126	0.145124	0.678334
C	-0.880786	2.980346	-2.387629
H	-0.127328	2.388440	-2.913395
H	-1.872203	2.793282	-2.810355
H	-0.651161	4.041274	-2.510589
H	0.175066	2.725560	-0.546092
C	-1.753785	1.389096	2.907763
H	-1.745909	2.216318	3.622610
H	-2.772575	1.002531	2.826220
H	-1.110383	0.588677	3.283473
H	-0.234912	2.276499	1.682672
C	2.497802	0.488623	-1.095655
C	3.471471	-0.303258	1.124305

C	2.711681	-2.006928	1.822696
C	3.444830	-2.712874	2.780117
H	3.989848	-2.144177	3.527811
C	3.493888	-4.101477	2.761412
C	2.784722	-4.803166	1.788867
C	2.009887	-4.117342	0.858475
H	4.084885	-4.634653	3.500293
H	2.817512	-5.888925	1.764418
H	1.418601	-4.643143	0.114980
O	2.765955	0.125108	2.864492
O	3.010700	0.034987	0.635863
O	1.151321	-2.092389	-0.039727
Bi	1.579503	0.576987	-0.759432
O	1.807387	2.548034	-0.466837
H	1.611032	2.782021	0.456021
C	-5.704529	-1.041446	0.252458
C	-4.267577	-1.168052	0.721959
H	-5.738993	-1.063917	-0.849694
H	-6.116759	-0.069103	0.571391
C	-3.389739	-0.060139	0.179012
H	-4.248324	-1.159223	1.818193
H	-3.873970	-2.138547	0.397575
H	-3.379332	-0.066480	-0.916047
H	-3.741976	0.923861	0.513652
O	-6.428204	-2.115091	0.812353
O	-2.065671	-0.292910	0.662333
H	-7.343320	-2.045503	0.512769
H	0.443469	-1.622842	0.448877

ET4

E(scf) = -1378.54880646 a.u.

C	-1.287728	0.462857	1.573434
C	-2.248863	0.758410	-0.807228
C	-2.320127	0.919660	0.582579
C	-3.451520	1.548811	1.116548
H	-3.480368	1.702222	2.190692
C	-4.504494	1.951315	0.306876
C	-4.431124	1.748851	-1.069931
C	-3.299059	1.164679	-1.625398
H	-5.378954	2.421514	0.746883

C	3.581734	0.433882	-0.062731
C	4.770859	1.121997	-0.317685
H	4.832223	1.717875	-1.223594
C	5.851379	1.027424	0.550939
C	5.742537	0.254148	1.704769
C	4.548712	-0.397542	1.998767
H	6.775761	1.552131	0.328002
H	6.581905	0.172279	2.389827
H	4.427173	-0.974819	2.910277
O	2.215935	1.517368	-1.673480
O	1.965608	-0.695269	-1.397106
O	2.293417	-0.934501	1.479481
Bi	0.378853	-1.621365	-0.441338
O	-0.915630	-1.312553	-1.941844
H	-1.019792	-0.361462	-2.113742
C	-5.704529	-1.041446	0.252458
C	-4.267577	-1.168052	0.721959
H	-5.738993	-1.063917	-0.849694
H	-6.116759	-0.069103	0.571391
C	-3.389739	-0.060139	0.179012
H	-4.248324	-1.159223	1.818193
H	-3.87397	-2.138547	0.397575
H	-3.379332	-0.066480	-0.916047
H	-3.741976	0.923861	0.513652
O	-6.428204	-2.115091	0.812353
O	-2.065671	-0.292910	0.662333
H	-7.343320	-2.045503	0.512769
H	1.603955	-0.250580	1.610467

INT5

E(scf) = -1378.55283654 a.u.

C	3.264607	-1.487046	1.293219
C	2.236687	-2.306697	0.518551
C	1.424495	0.851190	0.188159
C	1.564321	0.229012	1.568895
O	2.865503	-0.275594	1.798690
O	1.709861	-1.489521	-0.498005
O	4.403593	-1.813521	1.492542
O	0.322360	0.806093	-0.400966
C	2.885430	-3.553823	-0.056083

H	-5.247943	2.059928	-1.715325
C	3.342399	-1.366475	1.476548
C	2.427269	-2.017104	0.441159
C	1.372468	0.685280	0.427208
C	1.539669	0.193569	1.854108
O	2.855577	-0.275840	2.126435
O	1.861931	-1.015869	-0.383123
O	4.444203	-1.753915	1.767027
O	0.158079	0.757406	-0.014788
C	3.177891	-3.051283	-0.400914
H	3.275596	-2.654538	-1.445228
H	4.194616	-3.228670	0.020875
H	2.621540	-4.024012	-0.431120
H	1.633203	-2.507644	1.022679
C	1.229850	1.300506	2.843243
H	1.313948	0.913833	3.864198
H	1.925770	2.140922	2.731446
H	0.211138	1.665817	2.681935
H	0.813764	-0.618930	1.967785
C	-3.035794	-0.238823	0.439256
C	-4.686590	1.369104	-0.641300
C	-4.262874	0.588414	0.457592
C	-5.031254	0.591231	1.631810
H	-4.679635	-0.018733	2.458113
C	-6.188793	1.340852	1.733579
C	-6.597791	2.111202	0.639137
C	-5.859893	2.127432	-0.531774
H	-6.771746	1.331152	2.649393
H	-7.505565	2.705976	0.703497
H	-6.164585	2.720287	-1.388576
O	-2.678047	-0.882069	1.426321
O	-2.369726	-0.245360	-0.706271
O	-4.020634	1.429178	-1.802629
Bi	-0.352959	-1.092536	-0.978330
O	-0.550153	-2.282083	0.617722
H	-1.355434	-1.965607	1.095347
C	5.651557	2.894950	-0.478413
C	4.151018	2.757008	-0.662641
H	6.152461	1.962285	-0.788080
H	5.884479	3.049058	0.588823
C	3.593226	1.609521	0.143908
H	3.667826	-3.278842	-0.768646
H	3.333460	-4.166560	0.731162
H	2.126330	-4.147781	-0.575188
H	1.452330	-2.605894	1.234072
C	1.252830	1.264413	2.644120
H	1.311208	0.778596	3.621689
H	1.957431	2.100856	2.631913
H	0.239730	1.653253	2.505391
H	0.812769	-0.565227	1.628571
C	-3.127293	-0.201496	0.442767
C	-4.420199	1.746860	-0.555668
C	-4.261501	0.753316	0.436263
C	-5.208497	0.661108	1.466362
H	-5.057779	-0.112005	2.213566
C	-6.291916	1.519337	1.528165
C	-6.439175	2.499651	0.540620
C	-5.518307	2.615195	-0.486746
H	-7.017362	1.432914	2.331377
H	-7.285121	3.181799	0.575332
H	-5.619169	3.371329	-1.259232
O	-2.996236	-1.020753	1.360053
O	-2.301981	-0.117164	-0.575455
O	-3.563162	1.910442	-1.572232
Bi	-0.379682	-1.29178	-0.916386
O	-0.690761	-2.173871	0.843719
H	-1.589072	-1.884754	1.173789
C	5.673858	3.275341	-0.18631
C	4.243836	2.979699	-0.604361
H	6.306879	2.392232	-0.374663
H	5.713334	3.478281	0.897498
C	3.676036	1.803906	0.157245
H	3.630098	3.871125	-0.432569
H	4.222253	2.770613	-1.679453
H	4.253733	0.890423	-0.003433
H	3.627735	1.993645	1.230886
O	6.100729	4.38858	-0.935715
O	2.343764	1.580902	-0.368334
H	7.016052	4.579179	-0.694812
H	-2.86967	1.220036	-1.452777

H	3.666512	3.696656	-0.360773
H	3.932409	2.605457	-1.725395
H	4.028960	0.650531	-0.163994
H	3.764222	1.737733	1.215085
O	6.069461	3.989995	-1.260865
O	2.183604	1.555662	-0.129663
H	7.023963	4.086940	-1.153147
H	-3.238526	0.842800	-1.697866

p

E(scf) = -1378.59748690 a.u.

C	-0.851512	-0.786487	-1.311740
C	0.006172	-1.888527	-1.908514
C	-3.632232	0.117773	-0.930048
C	-2.513488	0.818671	-1.693925
O	-1.621518	-0.172121	-2.202862
O	0.751576	-2.528966	-0.932552
O	-0.815326	-0.472010	-0.132542
O	-3.793626	-1.075578	-0.879679
C	-0.868767	-2.901093	-2.645907
H	-1.596502	-3.342135	-1.958227
H	-1.404593	-2.432967	-3.476073
H	-0.227158	-3.696929	-3.033104
H	0.664722	-1.383626	-2.638733
C	-3.037668	1.607231	-2.879544
H	-2.207015	2.086662	-3.404555
H	-3.559911	0.947806	-3.579393
H	-3.730231	2.378066	-2.533049
H	-1.975922	1.466513	-0.994183
C	2.677062	0.777175	0.391639
C	4.011976	2.840782	0.806347
C	3.321629	1.998915	-0.096583
C	3.262221	2.339594	-1.456277
H	2.729940	1.669948	-2.124626
C	3.866941	3.489581	-1.928355
C	4.546836	4.319600	-1.027574
C	4.621250	4.005417	0.318140
H	3.818628	3.744963	-2.982351
H	5.027341	5.226136	-1.387063
H	5.147793	4.640829	1.023187

O	2.048731	0.020920	-0.445291
O	2.719083	0.451572	1.608637
O	4.112235	2.578246	2.110844
Bi	1.333632	-1.617013	0.834605
O	3.021445	-2.713606	0.664364
H	3.073664	-3.032024	-0.250456
C	-7.485416	1.216971	1.793849
C	-6.292323	1.666646	0.970272
H	-7.143367	0.584305	2.630229
H	-8.156407	0.599679	1.172511
C	-5.520854	0.494246	0.407301
H	-6.646271	2.306904	0.154144
H	-5.635411	2.277112	1.600122
H	-5.128274	-0.159295	1.194232
H	-6.133493	-0.123247	-0.259025
O	-8.135762	2.378078	2.257533
O	-4.415292	1.024281	-0.340912
H	-8.891101	2.104572	2.792993
H	3.638822	1.723293	2.266404

Table S4. Cartesian coordinates (xyz format) of the optimized geometries for all the species involved in the energy profile of the **Scheme S3** calculated at the D3-PBE0/[6-31G(d), LANL2DZ] level.

R

E(scf) = -884.042376469 a.u.

C	-2.823199	-1.153539	0.881366
C	-2.313136	1.133812	-0.250784
C	-3.117424	0.271685	0.526505
C	-4.331078	0.770104	1.027818
H	-4.926494	0.083670	1.621177
C	-4.758833	2.062505	0.783173
C	-3.958446	2.903381	0.007695
C	-2.756015	2.442579	-0.499933
H	-5.703458	2.414012	1.187055
H	-4.272640	3.922738	-0.202186
H	-2.122012	3.081928	-1.107671
O	-3.597472	-1.804370	1.550700
O	-1.691581	-1.711397	0.443773
O	-1.125386	0.815058	-0.805670
Bi	-0.029061	-0.937921	-0.536669
O	0.847846	-0.586986	1.252835
H	0.216120	-0.780034	1.962896
C	5.933725	0.237498	0.598528
H	5.634129	-0.741076	1.010184
H	6.293534	0.062033	-0.429917
C	4.731423	1.161247	0.565115
C	3.594835	0.607936	-0.286947
H	4.391244	1.321342	1.596040
H	5.058072	2.137322	0.183160
C	2.395640	1.530197	-0.289509
H	3.281625	-0.368552	0.105033
H	3.930190	0.459660	-1.322207
H	2.677245	2.527511	-0.651368
H	1.977904	1.614880	0.719409
O	1.402561	0.995937	-1.180484
O	6.924887	0.850381	1.393128
H	0.620960	1.583214	-1.177309
H	7.683840	0.255406	1.436841

ET1

E(scf) = -884.018808297 a.u.

C	-1.962220	0.140711	1.700453
C	-2.422405	0.911682	-0.734251
C	-2.716619	0.883676	0.643698
C	-3.809857	1.633222	1.107065
H	-4.016711	1.594080	2.171525
C	-4.587503	2.397755	0.254358
C	-4.280436	2.425381	-1.106875
C	-3.211891	1.687921	-1.592126
H	-5.425287	2.968820	0.643202
H	-4.879493	3.018856	-1.792889
H	-2.963783	1.681765	-2.649380
O	-2.294585	0.201658	2.863863
O	-0.873604	-0.578138	1.382423
O	-1.418790	0.199289	-1.296516
Bi	-0.021754	-1.446047	-0.283798
O	1.547181	-1.685346	0.979324
H	1.335374	-1.249777	1.820939
C	5.540384	1.412273	0.049057
H	5.693362	0.397387	0.452843
H	5.817498	1.383126	-1.018707
C	4.078994	1.794086	0.182811
C	3.155890	0.831259	-0.555666
H	3.827961	1.822829	1.251232
H	3.954206	2.816731	-0.197320
C	1.698199	1.213628	-0.395957
H	3.283601	-0.189532	-0.175127
H	3.395414	0.817953	-1.627201
H	1.520437	2.230924	-0.765958
H	1.403500	1.187498	0.662307
O	0.864149	0.342678	-1.166524
O	6.303561	2.368425	0.752504
H	-0.292071	0.646165	-1.340706
H	7.233080	2.116943	0.684463

INT1

E(scf) = -884.026651081 a.u.

C	-1.521845	0.694539	1.643561
C	-2.175741	1.312298	-0.794741
C	-2.323549	1.418023	0.594970
C	-3.314111	2.281738	1.082213
H	-3.399931	2.379946	2.159503
C	-4.155877	2.979873	0.228858
C	-4.009022	2.839733	-1.149822
C	-3.014386	2.013579	-1.657347
H	-4.923293	3.631328	0.636395
H	-4.660497	3.380326	-1.830911
H	-2.861749	1.898022	-2.725973
O	-1.444205	1.154013	2.762721
O	-0.964578	-0.478252	1.364379
O	-1.229817	0.498349	-1.384756
Bi	-0.331007	-1.628777	-0.253198
O	0.792726	-2.607789	1.109468
H	0.834931	-2.088099	1.928351
C	5.364203	2.197866	-0.425547
H	5.684653	1.778821	-1.394823
H	4.849753	3.150560	-0.638163
C	4.398969	1.240514	0.245914
C	3.171175	0.951907	-0.610232
H	4.936139	0.309696	0.469784
H	4.101003	1.670983	1.210846
C	2.203368	0.003399	0.074420
H	3.468777	0.510908	-1.570843
H	2.642955	1.886891	-0.843120
H	1.864540	0.436930	1.028707
H	2.705435	-0.944963	0.311203
O	1.098161	-0.228925	-0.784341
O	6.462094	2.387895	0.440739
H	-0.296319	0.780230	-1.201915
H	7.073024	3.003766	0.017252

INT2

E(scf) = -1417.81369917 a.u.

C	-2.889951	-0.876745	2.403264
C	-2.104114	-2.165795	2.197835
C	-2.349088	-1.845210	-0.153004
C	-3.741084	-1.377978	0.226335
O	-3.655777	-0.502121	1.358625
O	-1.572868	-2.226621	0.857432
O	-2.870517	-0.236406	3.416560
O	-1.931961	-1.887890	-1.290378
C	-0.946388	-2.305477	3.154896
H	-0.482530	-3.288461	3.039188
H	-0.196480	-1.536051	2.953513
H	-1.308079	-2.192822	4.179048
H	-2.806946	-3.005040	2.314732
C	-4.430398	-0.634291	-0.890830
H	-4.535038	-1.285788	-1.761373
H	-5.420023	-0.309550	-0.560268
H	-3.843547	0.239889	-1.183408
H	-4.326672	-2.265817	0.513944
C	2.784627	1.215499	-0.797551
C	2.762288	0.024125	1.486768
C	3.342892	0.904650	0.562197
C	4.549384	1.526842	0.901106
H	4.965219	2.237697	0.193489
C	5.204710	1.234148	2.089613
C	4.638916	0.320575	2.976901
C	3.415303	-0.268855	2.682462
H	6.150636	1.714053	2.322940
H	5.139467	0.081295	3.911345
H	2.935164	-0.951977	3.376821
O	2.874415	2.333504	-1.262037
O	2.313192	0.193158	-1.500944
O	1.544495	-0.573026	1.273528
Bi	0.661870	-1.091177	-1.321389
O	0.093613	-0.394389	-3.128419
H	0.673011	0.356597	-3.338835
C	-0.736273	1.555110	-0.807597
C	-1.519241	2.400387	0.181701
H	-1.353977	1.319958	-1.686119

H	0.137975	2.117701	-1.166912
C	-2.014731	3.700999	-0.440877
H	-2.366386	1.813245	0.560620
H	-0.876379	2.616568	1.046117
C	-2.782746	4.559590	0.544934
H	-1.171243	4.286366	-0.828584
H	-2.669627	3.490032	-1.296943
H	-3.630768	3.983975	0.953936
H	-2.128366	4.819511	1.393907
O	-0.330173	0.347841	-0.172496
O	-3.226812	5.714613	-0.135573
H	0.858471	0.094727	0.997157
H	-3.684603	6.276854	0.501715

ET2

E(scf) = -1417.792871800 a.u.

C	1.806552	3.227392	0.641905
C	1.364879	2.621620	1.970209
C	-0.634401	1.868847	0.859465
C	-0.520053	3.222424	0.182540
O	0.826302	3.459451	-0.249054
O	0.307049	1.667420	1.831888
O	2.947119	3.518963	0.396792
O	-1.797235	1.434145	1.159209
C	2.506918	1.947572	2.694160
H	2.167987	1.591564	3.672102
H	2.866585	1.091853	2.114787
H	3.328596	2.650878	2.830509
H	0.981765	3.460146	2.578748
C	-1.462572	3.416279	-0.980893
H	-2.485188	3.489199	-0.602476
H	-1.215126	4.348965	-1.495789
H	-1.426691	2.584876	-1.687171
H	-0.759199	3.958455	0.966503
C	-0.043933	-2.668509	-0.769096
C	0.224023	-1.937577	1.726782
C	0.605630	-2.670936	0.596147
C	1.760979	-3.461092	0.704583
H	2.049216	-4.017934	-0.181296
C	2.512708	-3.516153	1.866616

INT3

E(scf) = -1417.81629945 a.u.

C	-3.113825	-0.885839	2.511724
C	-1.616974	-0.603433	2.534322
C	-1.398103	-1.279496	0.213999
C	-2.492489	-2.265565	0.693239
O	-3.520831	-1.607437	1.448180
O	-1.212972	-0.253000	1.206225
O	-3.898871	-0.484730	3.328059
O	-0.266735	-2.014437	0.057549
C	-1.276168	0.543621	3.456927
H	-0.193123	0.692417	3.497774
H	-1.754045	1.463727	3.109912
H	-1.643171	0.327405	4.462674
H	-1.082894	-1.513406	2.851375
C	-3.155084	-3.003928	-0.444179
H	-2.391563	-3.499814	-1.049252
H	-3.842574	-3.756731	-0.048959
H	-3.716464	-2.316955	-1.082407
H	-1.986123	-2.981616	1.351506
C	1.526299	1.793576	-0.630310
C	2.447414	0.628981	1.466989
C	2.487762	1.657006	0.515170
C	3.472648	2.643156	0.646080
H	3.466997	3.452778	-0.077121
C	4.420988	2.584851	1.657272

C	2.122485	-2.760445	2.970670	C	4.383262	1.537260	2.577108
C	0.979692	-1.978899	2.899829	C	3.393128	0.566537	2.487475
H	3.401246	-4.140199	1.912516	H	5.185137	3.352783	1.731836
H	2.699167	-2.786673	3.891929	H	5.118826	1.482048	3.374863
H	0.648807	-1.399333	3.758916	H	3.327067	-0.246845	3.203627
O	0.502676	-3.259930	-1.682583	O	1.309145	2.880818	-1.122419
O	-1.198261	-2.053686	-0.955279	O	0.923965	0.703497	-1.088343
O	-0.923416	-1.178769	1.725294	O	1.518755	-0.387598	1.417512
Bi	-2.257239	-0.373286	-0.269150	Bi	1.375127	-1.345348	-1.040881
O	-2.496271	0.290794	-2.182804	O	0.463774	-1.571481	-2.809983
H	-1.912878	-0.170573	-2.803072	H	-0.431153	-1.198281	-2.746863
C	0.666673	0.664782	-1.460847	C	-2.710685	0.355451	-1.028318
C	2.085910	0.257850	-1.102615	C	-2.134033	1.758275	-1.045832
H	0.653249	1.645737	-1.950441	H	-3.405018	0.217533	-0.193679
H	0.255964	-0.062072	-2.174479	H	-3.265824	0.159164	-1.953305
C	2.925155	0.028470	-2.357028	C	-3.209602	2.810536	-1.289120
H	2.555785	1.031989	-0.484701	H	-1.627010	1.945071	-0.092698
H	2.050359	-0.658177	-0.502787	H	-1.358651	1.815170	-1.817883
C	4.366967	-0.311271	-2.029322	C	-2.634071	4.213498	-1.303093
H	2.498376	-0.793496	-2.946201	H	-3.711458	2.634900	-2.250283
H	2.918222	0.921200	-2.994577	H	-3.987930	2.761197	-0.515539
H	4.812800	0.508145	-1.440712	H	-2.148203	4.421156	-0.335030
H	4.395979	-1.221053	-1.403475	H	-1.851374	4.282059	-2.075820
O	-0.213875	0.663191	-0.342563	O	-1.680974	-0.645545	-0.987382
O	5.054779	-0.501412	-3.248538	O	-3.691879	5.116657	-1.552848
H	-0.780885	-0.352527	2.222091	H	0.591554	-0.057035	1.341068
H	5.970067	-0.721719	-3.039022	H	-3.318363	6.006297	-1.581755

ET3

E(scf) = 1478.816071610 a.u.

C	-1.333483	3.627545	-0.079596
C	-0.435782	2.731118	-0.919405
C	-0.848641	0.855284	0.505817
C	-0.880082	1.984353	1.565622
O	-1.668417	3.093169	1.121057
O	-0.950778	1.401843	-0.785248
O	-1.774589	4.691058	-0.420072
O	0.331563	0.173425	0.670579
C	-0.440099	3.097590	-2.382465
H	0.270518	2.459107	-2.913374

INT4

E(scf) = -1417.81418429 a.u.

C	-1.333483	3.627545	-0.079596
C	-0.435782	2.731118	-0.919405
C	-0.848641	0.855284	0.505817
C	-0.880082	1.984353	1.565622
O	-1.668417	3.093169	1.121057
O	-0.950778	1.401843	-0.785248
O	-1.774589	4.691058	-0.420072
O	0.331563	0.173425	0.670579
C	-0.440099	3.097590	-2.382465
H	0.270518	2.459107	-2.913374

H	-1.442264	2.981382	-2.805261	H	-1.442264	2.981382	-2.805261
H	-0.138727	4.141118	-2.499090	H	-0.138727	4.141118	-2.499090
H	0.597306	2.762673	-0.543364	H	0.597306	2.762673	-0.543364
C	-1.417586	1.538044	2.904106	C	-1.417586	1.538044	2.904106
H	-0.830220	0.693222	3.274440	H	-0.830220	0.693222	3.274440
H	-1.351078	2.359183	3.622932	H	-1.351078	2.359183	3.622932
H	-2.461100	1.224081	2.822867	H	-2.461100	1.224081	2.822867
H	0.157384	2.322804	1.680142	H	0.157384	2.322804	1.680142
C	2.893487	-0.729357	1.936166	C	2.764693	0.367553	-1.067919
C	2.610241	-2.291853	-0.057171	C	3.661922	-0.521254	1.145680
C	3.075013	-2.044748	1.241923	C	3.835795	0.221329	-0.030355
C	3.774356	-3.054192	1.908330	C	5.075019	0.820962	-0.268934
H	4.099468	-2.864380	2.927076	H	5.187835	1.422719	-1.166002
C	4.067136	-4.256975	1.276533	C	6.139788	0.631931	0.604055
C	3.638787	-4.470502	-0.031835	C	5.965265	-0.147389	1.745591
C	2.897422	-3.495393	-0.692043	C	4.723381	-0.709928	2.024172
H	4.629800	-5.023647	1.801054	H	7.102792	1.087822	0.393613
H	3.863969	-5.406640	-0.535314	H	6.791777	-0.302758	2.433552
H	2.519517	-3.654068	-1.697483	H	4.552495	-1.288656	2.926819
O	2.596551	-0.658512	3.109913	O	2.552445	1.422520	-1.627264
O	3.196766	0.330193	1.186281	O	2.159781	-0.774991	-1.392923
O	1.836316	-1.370674	-0.738213	O	2.437312	-1.065416	1.485633
Bi	1.913123	1.352014	-0.077619	Bi	0.498245	-1.594927	-0.467557
O	1.751745	2.967855	1.098073	O	-0.765131	-1.183020	-1.968706
H	1.369510	2.729395	1.959664	H	-0.816626	-0.224211	-2.121482
C	-3.163425	0.237042	0.170563	C	-3.163425	0.237042	0.170563
C	-4.116808	-0.828641	0.670496	C	-4.116808	-0.828641	0.670496
H	-3.155931	0.270139	-0.923684	H	-3.155931	0.270139	-0.923684
H	-3.453252	1.227773	0.542414	H	-3.453252	1.227773	0.542414
C	-5.544466	-0.582663	0.196623	C	-5.544466	-0.582663	0.196623
H	-3.763055	-1.805652	0.314380	H	-3.763055	-1.805652	0.314380
H	-4.078207	-0.858069	1.767878	H	-4.078207	-0.858069	1.767878
C	-6.508633	-1.648535	0.680389	C	-6.508633	-1.648535	0.680389
H	-5.903949	0.392722	0.549494	H	-5.903949	0.392722	0.549494
H	-5.583633	-0.554146	-0.899825	H	-5.583633	-0.554146	-0.899825
H	-6.178015	-2.636525	0.316939	H	-6.178015	-2.636525	0.316939
H	-6.493815	-1.686431	1.783218	H	-6.493815	-1.686431	1.783218
O	-1.857263	-0.100236	0.643790	O	-1.857263	-0.100236	0.643790
O	-7.791797	-1.323201	0.193399	O	-7.791797	-1.323201	0.193399
H	1.001153	-1.241183	-0.241920	H	1.796811	-0.333670	1.607608
H	-8.407566	-2.002967	0.494418	H	-8.407566	-2.002967	0.494418

ET4

E(scf) = -1417.81506078 a.u.

C	2.879029	-2.177520	1.290617
C	2.072027	-2.331054	0.006946
C	1.193066	0.293131	0.848995
C	1.140515	-0.679728	2.017917
O	2.379758	-1.340010	2.234619
O	1.658735	-1.055411	-0.452978
O	3.915599	-2.746476	1.515048
O	0.051007	0.641457	0.343474
C	2.892532	-3.030755	-1.061250
H	2.280862	-3.184250	-1.957739
H	3.768056	-2.427843	-1.331928
H	3.242443	-4.003046	-0.703609
H	1.192193	-2.945329	0.258554
C	0.747385	0.022829	3.305113
H	-0.223742	0.508742	3.178273
H	0.673098	-0.714381	4.110342
H	1.487479	0.774428	3.595693
H	0.362609	-1.407949	1.747483
C	-3.265638	-0.235589	0.180635
C	-4.716545	1.783350	-0.360708
C	-4.451247	0.621628	0.392562
C	-5.354817	0.243167	1.402828
H	-5.127093	-0.653612	1.963092
C	-6.484895	0.992204	1.675223
C	-6.732137	2.146399	0.923537
C	-5.862868	2.538969	-0.079702
H	-7.170531	0.688358	2.460227
H	-7.616419	2.745967	1.124888
H	-6.041879	3.431535	-0.671105
O	-3.057122	-1.228427	0.879580
O	-2.467670	0.129235	-0.811506
O	-3.916949	2.221659	-1.344966
Bi	-0.467179	-0.735829	-1.220361
O	-0.896708	-2.384182	-0.168770
H	-1.752351	-2.194971	0.301935
C	3.449493	1.079252	1.266393
C	4.208103	2.372052	0.985288
H	3.936754	0.218127	0.782109

INT5

E(scf) = -1417.81898164 a.u.

C	2.716758	-2.050380	1.298784
C	1.560926	-2.839019	0.683186
C	1.172939	0.418932	0.015696
C	1.234139	-0.130127	1.432418
O	2.480155	-0.752288	1.681055
O	1.098146	-2.133343	-0.441682
O	3.825147	-2.476829	1.477911
O	0.110051	0.377331	-0.634006
C	2.028232	-4.232385	0.300031
H	1.182437	-4.796005	-0.106491
H	2.812051	-4.172434	-0.459750
H	2.426124	-4.769775	1.165587
H	0.772958	-2.923152	1.450895
C	1.004818	0.979729	2.451848
H	-0.007080	1.376781	2.329075
H	1.086271	0.543531	3.451211
H	1.729640	1.793986	2.370178
H	0.409351	-0.838998	1.536202
C	-2.844197	0.512891	0.579462
C	-4.290733	2.148797	-0.718853
C	-3.749969	1.682107	0.500296
C	-4.074829	2.350011	1.689947
H	-3.647207	1.963760	2.610121
C	-4.906864	3.455113	1.691949
C	-5.432912	3.911223	0.477917
C	-5.131268	3.270377	-0.711547
H	-5.149747	3.959951	2.622004
H	-6.088825	4.778207	0.462867
H	-5.533898	3.612033	-1.660093
O	-2.351789	0.158333	1.659205
O	-2.606975	-0.112600	-0.550107
O	-4.039027	1.568879	-1.899514
Bi	-0.937519	-1.641792	-0.808705
O	-1.347117	-2.259172	1.054639
H	-1.857469	-1.530026	1.486129
C	3.478388	1.250104	0.034180
C	4.129843	2.359528	-0.760545
H	4.026328	0.308623	-0.045680

H	3.376106	0.867141	2.342781	H	3.389319	1.505182	1.091093
C	5.227958	2.239437	-0.155746	C	5.537886	2.649443	-0.251387
H	3.466113	3.158586	0.761461	H	4.157453	2.068533	-1.818209
H	4.732682	2.691775	1.907182	H	3.506476	3.260602	-0.694197
C	5.979203	3.532212	-0.413522	C	6.218949	3.752075	-1.040133
H	5.969942	1.458477	0.089830	H	5.511899	2.948270	0.804512
H	4.724345	1.921099	-1.090711	H	6.161262	1.748016	-0.307622
H	5.266513	4.311717	-0.751557	H	6.282110	3.460375	-2.102155
H	6.424644	3.888811	0.535147	H	5.610220	4.671009	-0.990635
O	2.121098	1.215335	0.721157	O	2.162445	1.047039	-0.545276
O	6.964262	3.272494	-1.385125	O	7.497363	3.948221	-0.480434
H	-3.172955	1.577208	-1.394076	H	-3.448475	0.804246	-1.702428
H	7.433917	4.097914	-1.563353	H	7.943043	4.640927	-0.983933

p

E(scf) = -1417.83985041 a.u.

C	-1.265655	0.023488	-0.052064
C	-0.083299	-0.683654	-0.692699
C	-4.093024	0.565771	-0.674935
C	-2.960448	1.572013	-0.506677
O	-1.740669	0.963391	-0.900816
O	0.615684	-1.348922	0.333697
O	-1.743048	-0.208766	1.027721
O	-4.006665	-0.484808	-1.260387
C	-0.597824	-1.662151	-1.747462
H	0.250025	-2.133486	-2.254469
H	-1.204730	-2.435451	-1.266271
H	-1.218783	-1.146350	-2.484985
H	0.542870	0.079410	-1.171276
C	-3.166276	2.793768	-1.384880
H	-4.099738	3.294909	-1.115560
H	-2.336018	3.492404	-1.248834
H	-3.208179	2.504726	-2.439596
H	-2.909794	1.850099	0.550953
C	3.443104	0.779753	-0.313456
C	4.905969	2.250571	1.158092
C	4.180110	2.025396	-0.033660
C	4.145125	3.030488	-1.014127
H	3.577841	2.826673	-1.916873
C	4.805028	4.231280	-0.835854

C	5.520498	4.443844	0.348564
C	5.571742	3.471420	1.331663
H	4.768810	4.998228	-1.603129
H	6.044667	5.383424	0.503760
H	6.122298	3.623472	2.254632
O	2.799374	0.602230	-1.343838
O	3.520002	-0.156839	0.633389
O	5.000246	1.356819	2.151577
Bi	2.504218	-2.027137	0.278884
O	2.887597	-2.174586	-1.684137
H	2.846363	-1.276195	-2.061419
C	-6.358790	0.178133	-0.211767
C	-7.506323	0.859016	0.501735
H	-6.119050	-0.793073	0.234330
H	-6.579676	0.009026	-1.271635
C	-8.779818	0.022636	0.444487
H	-7.217070	1.040852	1.545013
H	-7.676963	1.842740	0.044658
C	-9.943073	0.686716	1.155450
H	-9.072283	-0.160440	-0.597646
H	-8.612870	-0.961412	0.901241
H	-9.678020	0.861874	2.211995
H	-10.138242	1.673331	0.701468
O	-5.209640	1.031405	-0.104912
O	-11.062168	-0.164298	1.038781
H	4.483951	0.569502	1.877360
H	-11.803483	0.252155	1.495850

Table S5. Cartesian coordinates (xyz format) of the optimized geometries for all the species involved in the energy profile of the **Scheme S4** calculated at the D3-PBE0/[6-31G(d), LANL2DZ] level.

R

E(scf) = -923.307013398 a.u.

C	-3.077636	-1.180731	0.952296
C	-2.643601	1.119371	-0.185968
C	-3.400879	0.244685	0.623988
C	-4.591732	0.728978	1.189641
H	-5.150704	0.032916	1.806777
C	-5.041990	2.019431	0.977167
C	-4.288818	2.872739	0.168693
C	-3.109813	2.426002	-0.402426
H	-5.967982	2.359662	1.430795
H	-4.621774	3.890743	-0.017570
H	-2.512942	3.075048	-1.036932
O	-3.814662	-1.844480	1.650507
O	-1.962006	-1.723353	0.458992
O	-1.482948	0.815149	-0.802555
Bi	-0.350672	-0.921974	-0.583569
O	0.586059	-0.556267	1.172671
H	-0.012403	-0.772145	1.904628
C	6.809609	0.759682	1.102262
C	5.648659	0.255631	0.267448
H	7.098829	1.769095	0.762274
H	6.494324	0.850334	2.155753
H	5.381090	-0.749636	0.617696
H	5.990407	0.146193	-0.770074
C	4.432722	1.173237	0.327775
C	3.263004	0.647427	-0.498752
H	4.112329	1.291535	1.372769
H	4.711491	2.176721	-0.026291
C	2.059390	1.561286	-0.430345
H	2.972885	-0.342734	-0.124378
H	3.564108	0.529663	-1.548023
H	2.315765	2.565832	-0.791644
H	1.688950	1.629144	0.598308
O	7.874705	-0.155252	0.960512
O	1.028000	1.031078	-1.279458

ET1

E(scf) = -923.277934045 a.u.

C	-2.363650	0.278703	1.557086
C	-2.094566	1.506360	-0.713800
C	-2.633810	1.382615	0.584340
C	-3.492813	2.390941	1.050144
H	-3.893669	2.271072	2.051320
C	-3.810559	3.496097	0.279425
C	-3.265609	3.613600	-1.000149
C	-2.418637	2.627134	-1.488912
H	-4.475248	4.261399	0.668912
H	-3.503236	4.473341	-1.621510
H	-1.997414	2.688652	-2.488027
O	-2.887231	0.267759	2.649728
O	-1.490669	-0.698806	1.252125
O	-1.292704	0.575648	-1.280578
Bi	-0.654602	-1.555502	-0.426212
O	0.554028	-2.417473	0.951864
H	0.323440	-2.066036	1.830114
C	6.229897	1.267421	0.124562
C	4.795401	1.418864	0.626433
H	6.847477	0.795219	0.915185
H	6.242835	0.586733	-0.758303
H	4.813101	2.093798	1.506819
H	4.208132	1.947668	-0.152325
C	4.159656	0.061829	0.972735
C	3.106492	-0.421586	-0.030870
H	4.948453	-0.701977	1.041346
H	3.704035	0.098436	1.969181
C	1.814751	0.367042	0.080632
H	2.862783	-1.478859	0.158366
H	3.487935	-0.345991	-1.058936
H	1.987202	1.438843	-0.099057
H	1.390120	0.263801	1.090899
O	6.706339	2.556038	-0.206541
O	0.872028	-0.063257	-0.901668

H	8.610610	0.156736	1.501799
H	0.244094	1.613280	-1.235709

INT1

E(scf) = -923.291465206 a.u.

C	-1.746773	0.818766	1.643021
C	-2.245042	1.577415	-0.792350
C	-2.393248	1.686717	0.597053
C	-3.223732	2.704116	1.086826
H	-3.307621	2.799807	2.164479
C	-3.914976	3.553342	0.235272
C	-3.773517	3.410559	-1.143682
C	-2.933152	2.428805	-1.653007
H	-4.561309	4.324203	0.644473
H	-4.308019	4.068529	-1.823319
H	-2.786448	2.304563	-2.721490
O	-1.609899	1.239805	2.771769
O	-1.399395	-0.429488	1.350814
O	-1.443229	0.621239	-1.383235
Bi	-0.877678	-1.626013	-0.273014
O	0.069833	-2.762502	1.102735
H	0.139453	-2.263506	1.932634
C	6.630734	1.280515	0.489094
C	5.401521	1.159024	-0.389925
H	6.376522	1.843411	1.403512
H	6.957400	0.275659	0.807661
H	5.679477	0.613191	-1.301008
H	5.101690	2.166149	-0.707678
C	4.240747	0.459952	0.308878
C	3.005197	0.336243	-0.577703
H	4.557318	-0.542176	0.633725
H	3.978939	1.009432	1.225096
C	1.854842	-0.361445	0.125347
H	3.249102	-0.222878	-1.490419
H	2.674773	1.333219	-0.899423
H	1.577457	0.192539	1.036404
H	2.157690	-1.369753	0.441078
O	7.636500	1.935779	-0.254500
O	0.746888	-0.438921	-0.758125
H	8.416357	2.018521	0.308384

H	7.627125	2.459247	-0.507895
H	-0.094701	0.627898	-1.139720

INT2

E(scf) = -1457.07824395 a.u.

C	-2.219567	-1.914396	2.410914
C	-1.007390	-2.830685	2.299370
C	-1.282037	-2.769858	-0.068621
C	-2.758357	-2.833721	0.271545
O	-3.038772	-1.919533	1.339584
O	-0.450341	-2.771869	0.969272
O	-2.467575	-1.249651	3.376874
O	-0.844030	-2.720495	-1.198067
C	0.090467	-2.472195	3.269948
H	0.887608	-3.218763	3.224564
H	0.508052	-1.493459	3.019572
H	-0.318179	-2.438097	4.281989
H	-1.353421	-3.861873	2.469020
C	-3.640525	-2.472257	-0.897858
H	-3.467383	-3.167061	-1.722799
H	-4.689536	-2.521687	-0.595832
H	-3.412416	-1.461392	-1.244602
H	-2.979935	-3.856054	0.617492
C	2.287321	2.012471	-0.862025
C	2.641074	1.021037	1.491411
C	2.876759	2.004782	0.520189
C	3.748276	3.053903	0.833627
H	3.889252	3.830300	0.087822
C	4.424544	3.095049	2.045345
C	4.214852	2.084648	2.981832
C	3.314800	1.061156	2.710478
H	5.111065	3.909085	2.258791
H	4.736081	2.103152	3.935203
H	3.104504	0.285115	3.440418
O	1.980881	3.059528	-1.395223
O	2.237263	0.853776	-1.506065
O	1.748656	-0.005666	1.301716
Bi	1.230750	-0.972846	-1.242911
O	0.512583	-0.660603	-3.103675
H	0.764034	0.244731	-3.351017

H	-0.476722	0.756792	-1.202587

ET2

E(scf) = -1457.05532141 a.u.

C	-0.126525	3.855369	1.090431
C	-0.835994	3.122033	2.218623
C	-1.782510	1.663600	0.553800
C	-1.910182	2.970242	-0.218393
O	-0.704087	3.743033	-0.117749
O	-1.309902	1.831726	1.821500
O	0.866432	4.519385	1.241220
O	-2.739102	0.814615	0.450556
C	0.053473	2.937779	3.425552
H	-0.500204	2.420467	4.214656
H	0.935583	2.348224	3.162814
H	0.388428	3.910084	3.792850
H	-1.713053	3.732153	2.492208
C	-2.274749	2.831385	-1.689572
H	-1.448403	3.219634	-2.301993
H	-2.456295	1.783900	-1.968055
H	-3.170600	3.419847	-1.926756
H	-2.703777	3.508301	0.318458

C	-4.994074	4.146525	-0.477133
C	-4.111234	3.126812	0.215010
H	-4.373118	4.978597	-0.850382
H	-5.475979	3.682898	-1.355456
H	-4.748690	2.323430	0.606766
H	-3.643441	3.605943	1.084773
C	-3.042176	2.549563	-0.705973
C	-2.144655	1.533656	-0.005069
H	-3.523671	2.074667	-1.574167
H	-2.423085	3.364313	-1.108550
C	-1.090312	0.957226	-0.933207
H	-2.749882	0.715360	0.406883
H	-1.642308	2.009912	0.847831
H	-0.468724	1.769154	-1.339381
H	-1.561936	0.452308	-1.788700
O	-5.954526	4.599807	0.453968
O	-0.291156	0.026310	-0.212190
H	-6.498115	5.267706	0.017814
H	0.866546	0.332807	0.985323

INT3

E(scf) = -1457.07972799 a.u.

C	-1.989707	-2.567623	2.476182
C	-0.815331	-1.597863	2.519618
C	-0.394902	-1.910428	0.151626
C	-0.859074	-3.335444	0.545026
O	-2.042895	-3.316557	1.356291
O	-0.693995	-0.998610	1.225403
O	-2.836551	-2.657641	3.323930
O	0.944500	-1.991330	-0.056154
C	-1.027318	-0.502582	3.538343
H	-0.151964	0.151982	3.580995
H	-1.908876	0.090498	3.280396
H	-1.190037	-0.943879	4.524149
H	0.105468	-2.156238	2.752476
C	-1.130788	-4.215250	-0.651164
H	-0.249335	-4.231184	-1.297553
H	-1.350841	-5.234301	-0.321462
H	-1.980869	-3.842286	-1.227866
H	-0.042193	-3.764627	1.137334

C	1.079951	-2.236546	-1.044223
C	0.606128	-2.369032	1.499042
C	1.267941	-2.799953	0.342225
C	2.192677	-3.843702	0.468720
H	2.731280	-4.144614	-0.424342
C	2.410237	-4.476265	1.685039
C	1.715239	-4.053968	2.816610
C	0.822897	-2.992521	2.724105
H	3.121364	-5.294391	1.751764
H	1.878062	-4.538929	3.775068
H	0.288296	-2.620817	3.592813
O	2.019244	-2.230382	-1.817734
O	-0.119309	-1.827994	-1.404333
O	-0.305627	-1.325839	1.489417
Bi	-1.923486	-1.067476	-0.516772
O	-2.280566	-0.420552	-2.405845
H	-1.508444	-0.651696	-2.946118
C	5.561447	1.577135	-1.549167
C	4.400735	1.420030	-0.586688
H	5.582792	0.719074	-2.241780
H	5.416947	2.485165	-2.159095
H	4.407547	2.269590	0.108554
H	4.570299	0.516439	0.013249
C	3.056245	1.331671	-1.301279
C	1.891439	1.143346	-0.333836
H	2.894810	2.241413	-1.894729
H	3.065335	0.486540	-2.003866
C	0.557629	1.104539	-1.060764
H	1.889047	1.948590	0.412709
H	2.035153	0.198455	0.208871
H	0.590559	0.365934	-1.868955
H	0.328453	2.078320	-1.499145
O	6.750557	1.655813	-0.790622
O	-0.512709	0.718279	-0.187083
H	7.489292	1.741511	-1.406004
H	0.100935	-0.478199	1.212916

ET3

E(scf) = 1457.08100521 a.u.

C	-0.704415	3.777878	-0.010198

C	0.619808	2.229993	-0.564859
C	2.035940	1.588062	1.484066
C	1.535000	2.539318	0.584825
C	1.889010	3.880430	0.773678
H	1.462652	4.609067	0.091177
C	2.753045	4.261796	1.790000
C	3.265985	3.296743	2.656174
C	2.900956	1.964030	2.508822
H	3.024811	5.306258	1.910108
H	3.942135	3.582955	3.457089
H	3.267347	1.195484	3.182597
O	-0.103518	3.089345	-1.021227
O	0.619455	1.001022	-1.067029
O	1.746440	0.244419	1.375743
Bi	2.019491	-0.555366	-1.121190
O	1.286056	-1.124941	-2.896854
H	0.321449	-1.211060	-2.820558
C	-2.385116	-1.062455	-0.967400
C	-2.620846	0.427436	-0.794949
H	-2.898573	-1.639339	-0.191220
H	-2.775718	-1.397141	-1.936027
C	-4.089412	0.787415	-0.999570
H	-2.298058	0.729655	0.207555
H	-1.990398	0.978566	-1.501558
C	-4.366354	2.268439	-0.762345
H	-4.392773	0.516302	-2.022282
H	-4.718471	0.185638	-0.325788
H	-4.116291	2.537276	0.272108
H	-3.723235	2.881872	-1.405693
O	-0.990625	-1.404956	-0.994468
C	-5.813691	2.640289	-1.016261
H	0.776219	0.063126	1.337845
H	-6.074784	2.414226	-2.064635
H	-6.890839	4.250414	-0.907407
O	-5.970978	4.014914	-0.733714
H	-6.470914	2.022959	-0.379269

INT4

E(scf) = -1457.07908328 a.u.

C	-0.780387	3.797258	-0.022232

C	0.131718	2.828700	-0.855575
C	-0.444699	0.965101	0.519350
C	-0.415958	2.068804	1.605891
O	-1.105522	3.245719	1.170748
O	-0.496862	1.546089	-0.758839
O	-1.051746	4.880946	-0.333487
O	0.607848	0.220047	0.664182
C	0.186221	3.225781	-2.309544
H	0.850703	2.541242	-2.842830
H	-0.813941	3.203664	-2.752074
H	0.575912	4.242619	-2.396777
H	1.156146	2.763284	-0.460703
C	-1.019659	1.635933	2.920337
H	-2.082187	1.406763	2.807496
H	-0.509725	0.739422	3.283755
H	-0.907201	2.432986	3.660249
H	0.641187	2.323547	1.752453
C	2.980007	0.219848	2.466581
C	2.201290	-2.204824	2.491513
C	2.804249	-1.111920	3.129723
C	3.301139	-1.279732	4.424623
H	3.735112	-0.417109	4.921816
C	3.264581	-2.521276	5.048283
C	2.704851	-3.609978	4.383238
C	2.159365	-3.448159	3.113319
H	3.673944	-2.640345	6.047148
H	2.673062	-4.585411	4.860867
H	1.682919	-4.272655	2.591641
O	2.792453	1.262882	3.056294
O	3.461797	0.148070	1.225496
O	1.610435	-2.083758	1.248034
Bi	2.346229	-0.005913	-0.512403
O	2.663022	1.889164	-1.089069
H	2.318412	2.512920	-0.428081
C	-7.654096	-0.869226	0.023690
C	-6.267102	-1.178892	0.551858
H	-7.954847	0.142681	0.345210
H	-7.636327	-0.866767	-1.079601
H	-5.993165	-2.192600	0.231428
H	-6.310118	-1.197889	1.648700
C	-5.217926	-0.177359	0.082531

C	0.055746	2.848080	-0.867609
C	-0.520671	0.984481	0.507316
C	-0.491930	2.088184	1.593857
O	-1.181494	3.265099	1.158714
O	-0.572834	1.565469	-0.770873
O	-1.127718	4.900326	-0.345521
O	0.607848	0.220047	0.664182
C	0.110249	3.245161	-2.321578
H	0.774731	2.560622	-2.854864
H	-0.889913	3.223044	-2.764108
H	0.499940	4.261999	-2.408811
H	1.080174	2.782664	-0.472737
C	-1.095631	1.655313	2.908303
H	-2.158159	1.426143	2.795462
H	-0.585697	0.758802	3.271721
H	-0.983173	2.452366	3.648215
H	0.565215	2.342927	1.740419
C	3.039223	0.185766	-1.057161
C	3.851269	-0.778317	1.155796
C	4.091962	-0.050261	-0.017793
C	5.378186	0.442528	-0.251484
H	5.544447	1.034211	-1.146984
C	6.420623	0.161444	0.623752
C	6.176850	-0.603569	1.762253
C	4.890740	-1.058897	2.036170
H	7.419561	0.534234	0.417262
H	6.985081	-0.830867	2.451970
H	4.668930	-1.623290	2.936838
O	2.913275	1.255124	-1.615254
O	2.343976	-0.904528	-1.381905
O	2.584182	-1.216592	1.491077
Bi	0.595691	-1.553366	-0.481347
O	-0.593321	-1.013269	-2.004096
H	-0.537907	-0.055814	-2.162031
C	-7.730068	-0.849846	0.011656
C	-6.343074	-1.159512	0.539824
H	-8.030819	0.162061	0.333176
H	-7.712299	-0.847387	-1.091635
H	-6.069137	-2.173220	0.219394
H	-6.386090	-1.178509	1.636666
C	-5.293898	-0.157979	0.070497

C	-3.821219	-0.502151	0.603082
H	-5.198732	-0.152506	-1.016663
H	-5.503977	0.833999	0.406803
C	-2.787822	0.497138	0.126227
H	-3.522436	-1.503304	0.264823
H	-3.823828	-0.526879	1.700903
H	-3.014934	1.505654	0.494276
H	-2.753318	0.530298	-0.967563
O	-8.537817	-1.850852	0.521713
O	-1.515972	0.073377	0.624765
H	-9.420869	-1.655874	0.183918
H	0.868530	-1.447644	1.319811

ET4

E(scf) = -1457.08036081 a.u.

C	2.404578	-2.404904	1.426088
C	1.503508	-2.679041	0.228415
C	0.964637	0.140200	0.639483
C	0.884270	-0.613738	1.959876
O	2.066563	-1.360224	2.225377
O	1.199414	-1.462539	-0.433264
O	3.388352	-3.041708	1.696244
O	-0.161359	0.525988	0.129780
C	2.170872	-3.650669	-0.734564
H	1.491364	-3.877317	-1.562609
H	3.086666	-3.211795	-1.140465
H	2.429373	-4.583900	-0.221200
H	0.579912	-3.133018	0.626546
C	0.656787	0.339305	3.118098
H	1.486247	1.036911	3.238664
H	-0.267516	0.901150	2.957814
H	0.563391	-0.240853	4.039932
H	0.023872	-1.279707	1.859024
C	-3.477489	0.128718	0.303625
C	-4.723088	2.169782	-0.566867
C	-4.517536	1.175918	0.416220
C	-5.328014	1.172690	1.561559
H	-5.146117	0.396044	2.297982
C	-6.317905	2.120615	1.746447
C	-6.510155	3.101994	0.767461

C	-3.897191	-0.482771	0.591048
H	-5.274704	-0.133126	-1.028697
H	-5.579949	0.853379	0.394769
C	-2.863794	0.516518	0.114193
H	-3.598408	-1.483924	0.252789
H	-3.899800	-0.507499	1.688869
H	-3.090906	1.525034	0.482242
H	-2.829290	0.549678	-0.979597
O	-8.613789	-1.831472	0.509679
O	-1.591944	0.092757	0.612731
H	-9.496841	-1.636494	0.171884
H	2.007957	-0.432817	1.608884

INT5

E(scf) = -1457.07908328 a.u.

C	-0.780387	3.797258	-0.022232
C	0.055746	2.848080	-0.867609
C	-0.520671	0.984481	0.507316
C	-0.491930	2.088184	1.593857
O	-1.181494	3.265099	1.158714
O	-0.572834	1.565469	-0.770873
O	-1.127718	4.900326	-0.345521
O	0.607848	0.220047	0.664182
C	0.110249	3.245161	-2.321578
H	0.774731	2.560622	-2.854864
H	-0.889913	3.223044	-2.764108
H	0.499940	4.261999	-2.408811
H	1.080174	2.782664	-0.472737
C	-1.095631	1.655313	2.908303
H	-2.158159	1.426143	2.795462
H	-0.585697	0.758802	3.271721
H	-0.983173	2.452366	3.648215
H	0.565215	2.342927	1.740419
C	3.039223	0.185766	-1.057161
C	3.851269	-0.778317	1.155796
C	4.091962	-0.050261	-0.017793
C	5.378186	0.442528	-0.251484
H	5.544447	1.034211	-1.146984
C	6.420623	0.161444	0.623752
C	6.176850	-0.603569	1.762253

C	-5.725948	3.129172	-0.372788	C	4.890740	-1.058897	2.036170
H	-6.936683	2.103129	2.638359	H	7.419561	0.534234	0.417262
H	-7.283891	3.854445	0.898387	H	6.985081	-0.830867	2.451970
H	-5.864131	3.883834	-1.140817	H	4.668930	-1.623290	2.936838
O	-3.299214	-0.699880	1.197445	O	2.913275	1.255124	-1.615254
O	-2.768508	0.141451	-0.817708	O	2.343976	-0.904528	-1.381905
O	-4.002340	2.250579	-1.693690	O	2.584182	-1.216592	1.491077
Bi	-0.927083	-1.031635	-1.139447	Bi	0.595691	-1.553366	-0.481347
O	-1.482036	-2.394498	0.215988	O	-0.593321	-1.013269	-2.004096
H	-2.233841	-1.992635	0.712841	H	-0.537907	-0.055814	-2.162031
C	7.441563	2.384759	-0.922306	C	-7.730068	-0.849846	0.011656
C	6.511304	2.872498	0.184730	C	-6.343074	-1.159512	0.539824
H	8.102150	1.584497	-0.517606	H	-8.030819	0.162061	0.333176
H	6.850108	1.939277	-1.750936	H	-7.712299	-0.847387	-1.091635
H	5.904489	3.706284	-0.209752	H	-6.069137	-2.173220	0.219394
H	7.138321	3.300329	0.988850	H	-6.386090	-1.178509	1.636666
C	5.604214	1.755530	0.732610	C	-5.293898	-0.157979	0.070497
C	4.160614	1.836910	0.213252	C	-3.897191	-0.482771	0.591048
H	6.031681	0.771900	0.477781	H	-5.274704	-0.133126	-1.028697
H	5.583029	1.797442	1.833683	H	-5.579949	0.853379	0.394769
C	3.324800	0.698780	0.748477	C	-2.863794	0.516518	0.114193
H	4.144085	1.822495	-0.887228	H	-3.598408	-1.483924	0.252789
H	3.707520	2.787806	0.525916	H	-3.899800	-0.507499	1.688869
H	3.360231	0.655492	1.843008	H	-3.090906	1.525034	0.482242
H	3.661951	-0.265809	0.357033	H	-2.829290	0.549678	-0.979597
O	8.201725	3.489442	-1.363897	O	-8.613789	-1.831472	0.509679
O	1.972763	0.915689	0.308598	O	-1.591944	0.092757	0.612731
H	8.812242	3.178929	-2.049481	H	-9.496841	-1.636494	0.171884
H	-3.361494	1.505307	-1.659492	H	2.007957	-0.432817	1.608884

p

E(scf) = -1457.08373899 a.u.

C	2.039566	-2.614841	1.326226
C	0.794960	-3.173342	0.635614
C	0.968859	0.116139	0.095114
C	0.891922	-0.481024	1.491349
O	2.007868	-1.310061	1.753986
O	0.497823	-2.351882	-0.465935
O	3.052078	-3.229257	1.527107
O	-0.068163	0.285526	-0.576962

C	1.041962	-4.606563	0.197686
H	0.132418	-5.001724	-0.265949
H	1.855745	-4.643793	-0.531435
H	1.308910	-5.241679	1.047157
H	-0.024849	-3.159481	1.374215
C	0.814666	0.619545	2.542997
H	1.665329	1.305756	2.509964
H	-0.113658	1.181851	2.406141
H	0.790066	0.146070	3.528311
H	-0.041150	-1.046814	1.546134
C	-3.006812	0.861021	0.553515
C	-4.089772	2.772739	-0.721404
C	-3.694713	2.171239	0.494617
C	-3.955553	2.837342	1.700996
H	-3.643696	2.346578	2.617859
C	-4.584695	4.069116	1.722433
C	-4.967133	4.657524	0.511538
C	-4.724736	4.021984	-0.694172
H	-4.780593	4.571189	2.664994
H	-5.462688	5.625261	0.511917
H	-5.017605	4.465916	-1.640562
O	-2.634546	0.383468	1.634061
O	-2.825727	0.250376	-0.594697
O	-3.886259	2.205472	-1.917315
Bi	-1.421718	-1.521457	-0.869850
O	-1.984998	-2.126539	0.956383
H	-2.390612	-1.339202	1.398836
C	7.978124	2.567634	-0.201585
C	6.560039	2.541750	-0.738360
H	7.962586	2.827436	0.870849
H	8.423281	1.561446	-0.285030
H	6.598381	2.295865	-1.807346
H	6.142999	3.554436	-0.664221
C	5.668300	1.548993	-0.001250
C	4.241953	1.532915	-0.545079
H	6.099785	0.540358	-0.074932
H	5.650291	1.796715	1.070170
C	3.380299	0.536742	0.197806
H	4.247510	1.275457	-1.611382
H	3.794543	2.531458	-0.461404
H	3.304531	0.776433	1.259684

H	3.756833	-0.484402	0.104141
O	8.704727	3.516523	-0.950872
O	2.066428	0.582100	-0.419395
H	9.610103	3.532504	-0.616231
H	-3.443146	1.344046	-1.733505

References

1. C. Adamo, V. Barone, *J. Chem. Phys.*, **1999**, 110,6158-6169.

2. S. Grimme, J. Antony, S. Ehrlich, H. Krieg, *J. Chem. Phys.*, **2010**, 132, 154104-1541019.

3. (a) T. H. Dunning Jr., P. J. Hay, Methods of Electronic Structure Theory, ed. H. F. Schaefer, Plenum Press, Nueva York, 3rd edn, **1977**, Vol. 2, pp. 1-27. (b) Y. Zhao, D.-G. Truhlar, *Theor. Chem. Acc.*, **2008**, 120, 215-241.
